# Supplementary figures and images for: Vangl2 suppresses NF-κB signaling and ameliorates sepsis by targeting p65 for NDP52-mediated autophagic degradation (part 1 of 2)
Source: eLife. 2024 Sep 13;12:RP87935. doi: 10.7554/eLife.87935 (PMC11398866; doi:10.7554/eLife.87935)

Figure 1

D

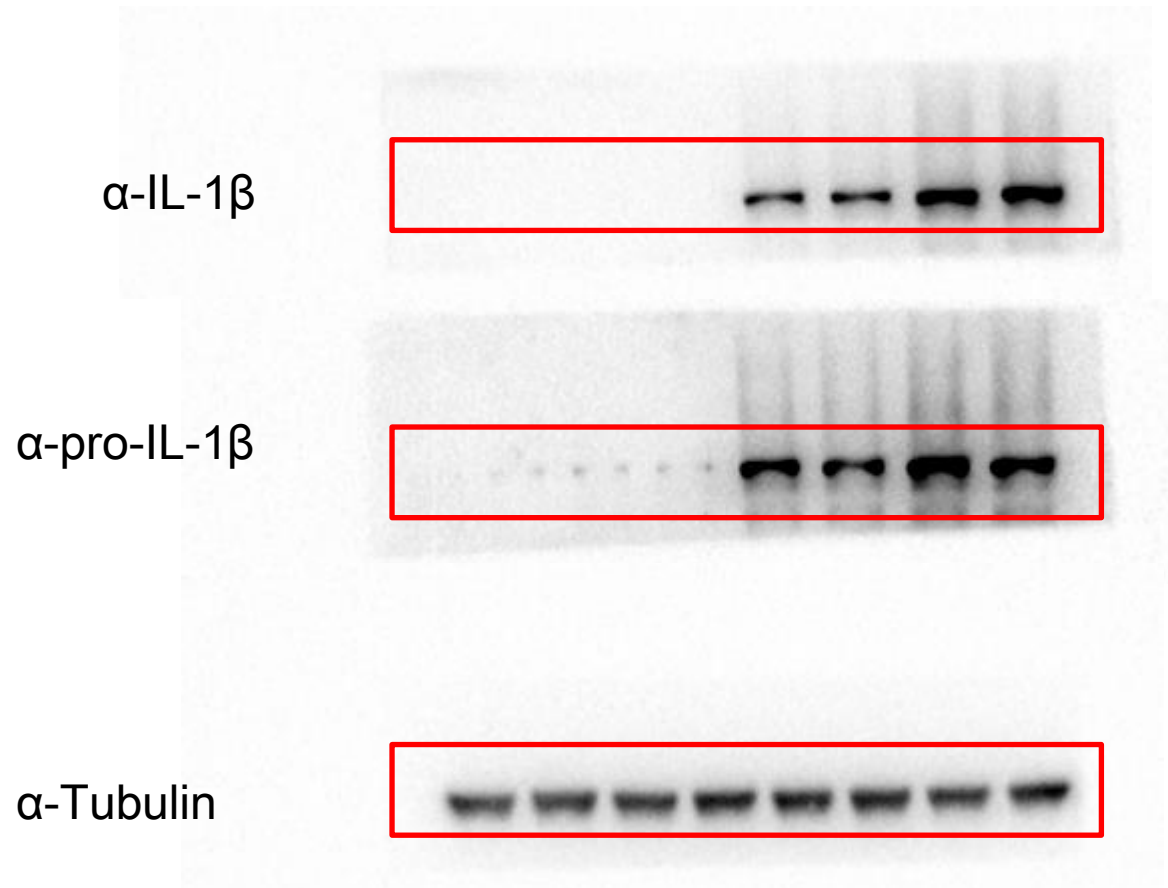

Figure 1-figure supplement 1

C-E

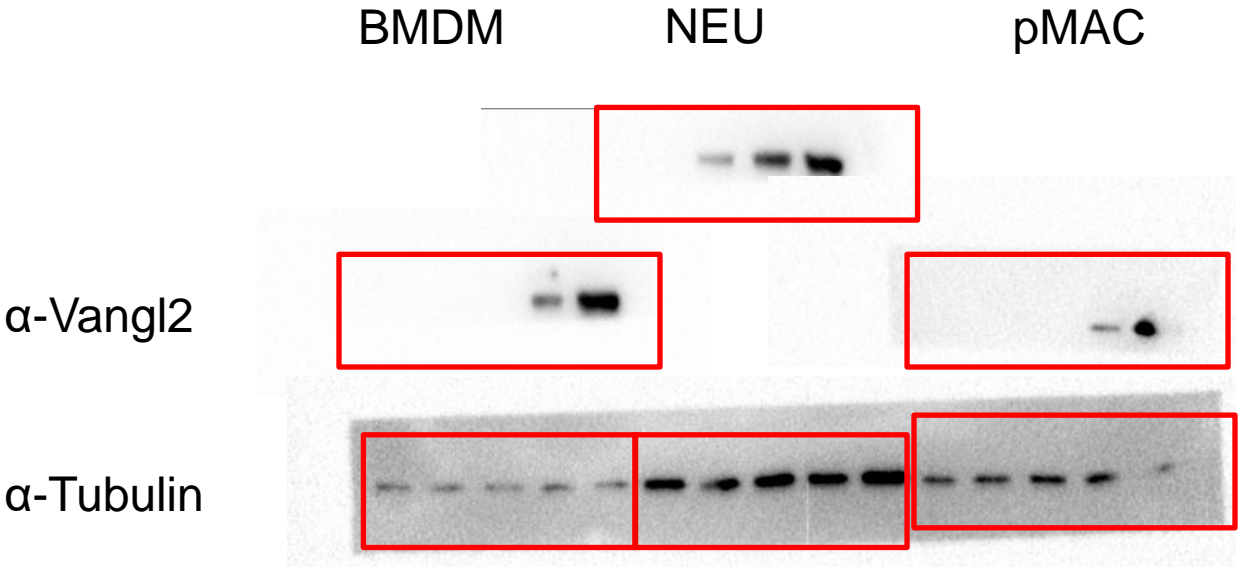

Supplement: Figure 1—source data 1. [file elife-87935-fig1-data1.pdf]

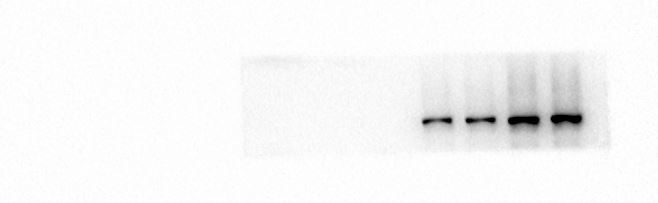

Supplement: Figure 1—source data 3. [file elife-87935-fig1-data3.zip › Figure 1-Source data 2/Fig1D-IL-1b.JPG]

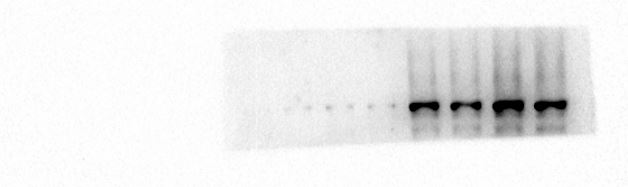

Supplement: Figure 1—source data 3. [file elife-87935-fig1-data3.zip › Figure 1-Source data 2/Fig1D-pro-IL-1b.JPG]

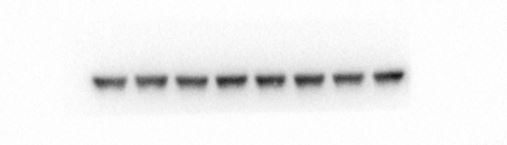

Supplement: Figure 1—source data 3. [file elife-87935-fig1-data3.zip › Figure 1-Source data 2/Fig1D-tubulin.JPG]

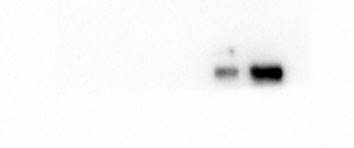

Supplement: Figure 1—source data 3. [file elife-87935-fig1-data3.zip › Figure 1-Source data 2/FigS1-C-Vangl2.JPG]

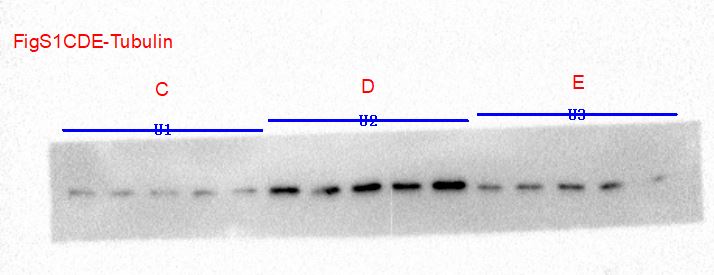

Supplement: Figure 1—source data 3. [file elife-87935-fig1-data3.zip › Figure 1-Source data 2/FigS1-CDE-tubulin.JPG]

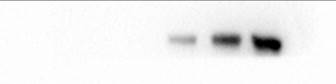

Supplement: Figure 1—source data 3. [file elife-87935-fig1-data3.zip › Figure 1-Source data 2/FigS1-D-Vangl2.JPG]

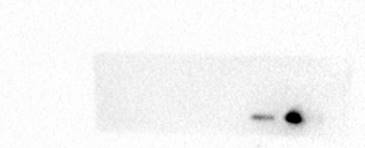

Supplement: Figure 1—source data 3. [file elife-87935-fig1-data3.zip › Figure 1-Source data 2/FigS1-E-Vangl2.JPG]

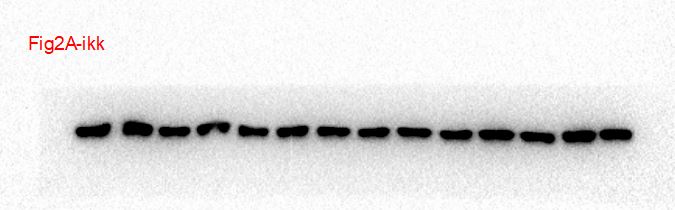

Supplement: Figure 2—source data 3. [file elife-87935-fig2-data3.zip › Figure 2-Source data 4/fig2-A/Fig2A-ikk.JPG]

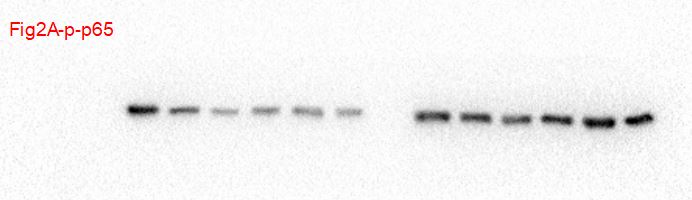

Supplement: Figure 2—source data 3. [file elife-87935-fig2-data3.zip › Figure 2-Source data 4/fig2-A/Fig2A-p-p65.JPG]

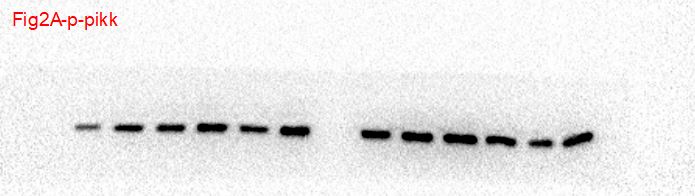

Supplement: Figure 2—source data 3. [file elife-87935-fig2-data3.zip › Figure 2-Source data 4/fig2-A/Fig2A-p-pikk.JPG]

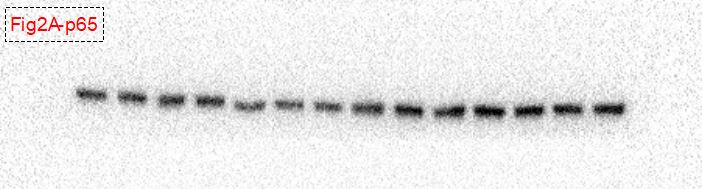

Supplement: Figure 2—source data 3. [file elife-87935-fig2-data3.zip › Figure 2-Source data 4/fig2-A/Fig2A-p65.JPG]

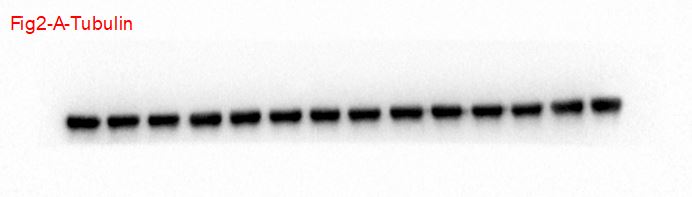

Supplement: Figure 2—source data 3. [file elife-87935-fig2-data3.zip › Figure 2-Source data 4/fig2-A/Fig2A-tubulin.JPG]

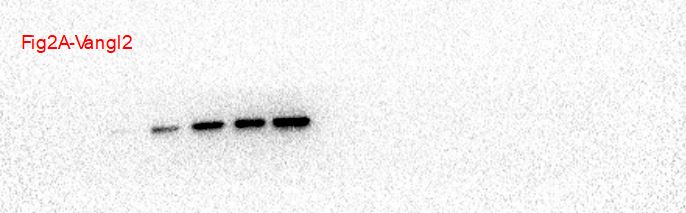

Supplement: Figure 2—source data 3. [file elife-87935-fig2-data3.zip › Figure 2-Source data 4/fig2-A/Fig2A-Vangl2.JPG]

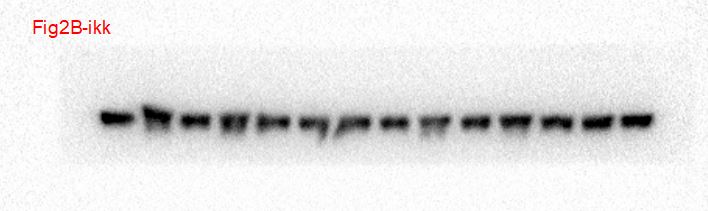

Supplement: Figure 2—source data 3. [file elife-87935-fig2-data3.zip › Figure 2-Source data 4/fig2-B/Fig2B-ikk.JPG]

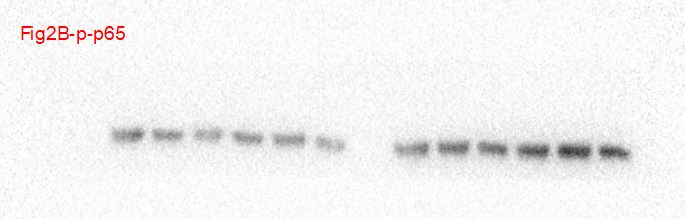

Supplement: Figure 2—source data 3. [file elife-87935-fig2-data3.zip › Figure 2-Source data 4/fig2-B/Fig2B-p-p65.JPG]

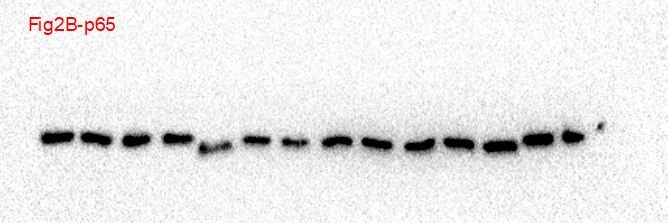

Supplement: Figure 2—source data 3. [file elife-87935-fig2-data3.zip › Figure 2-Source data 4/fig2-B/Fig2B-p65.JPG]

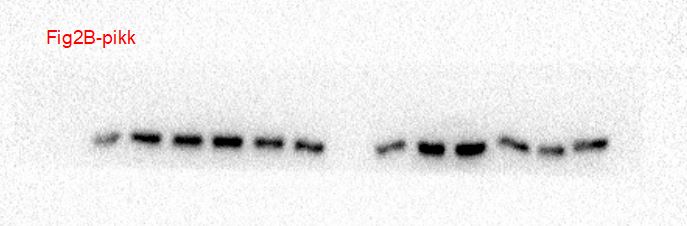

Supplement: Figure 2—source data 3. [file elife-87935-fig2-data3.zip › Figure 2-Source data 4/fig2-B/Fig2B-pikk.JPG]

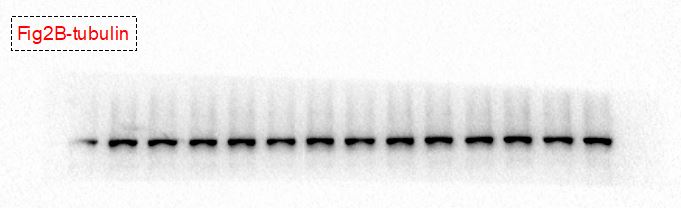

Supplement: Figure 2—source data 3. [file elife-87935-fig2-data3.zip › Figure 2-Source data 4/fig2-B/Fig2B-tubulin.JPG]

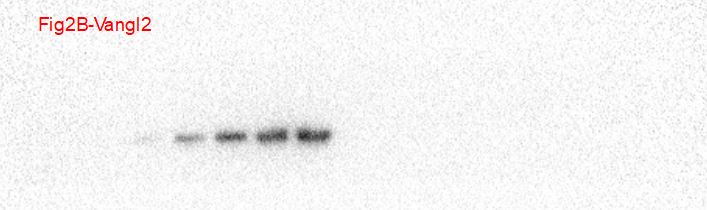

Supplement: Figure 2—source data 3. [file elife-87935-fig2-data3.zip › Figure 2-Source data 4/fig2-B/Fig2B-Vangl2.JPG]

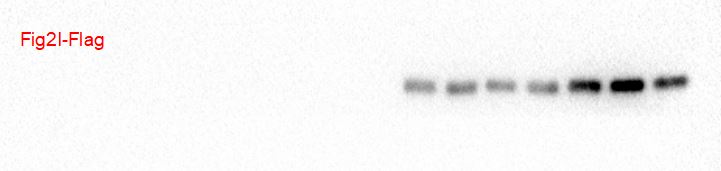

Supplement: Figure 2—source data 3. [file elife-87935-fig2-data3.zip › Figure 2-Source data 4/fig2-C/Fig2I-flag.JPG]

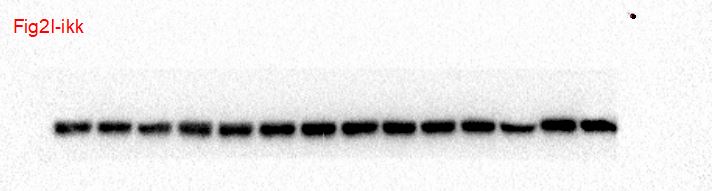

Supplement: Figure 2—source data 3. [file elife-87935-fig2-data3.zip › Figure 2-Source data 4/fig2-C/Fig2I-ikk.JPG]

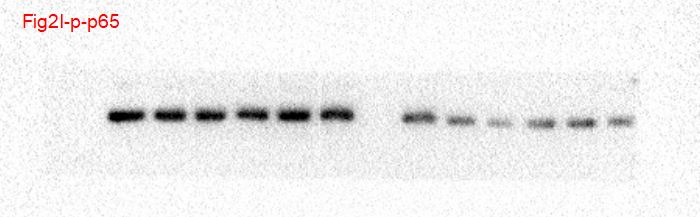

Supplement: Figure 2—source data 3. [file elife-87935-fig2-data3.zip › Figure 2-Source data 4/fig2-C/Fig2I-p-p65.JPG]

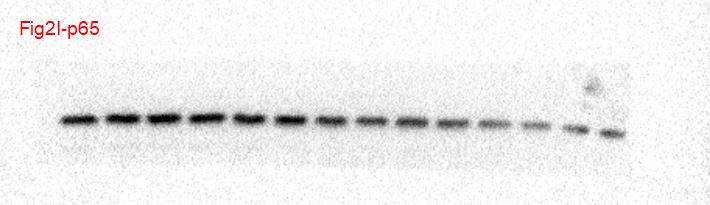

Supplement: Figure 2—source data 3. [file elife-87935-fig2-data3.zip › Figure 2-Source data 4/fig2-C/Fig2I-p65.JPG]

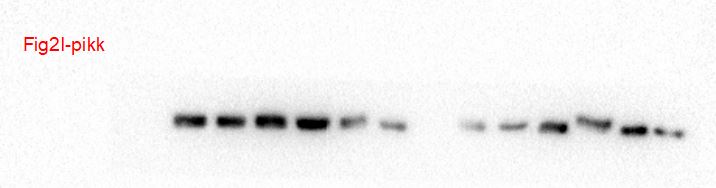

Supplement: Figure 2—source data 3. [file elife-87935-fig2-data3.zip › Figure 2-Source data 4/fig2-C/Fig2I-pikk.JPG]

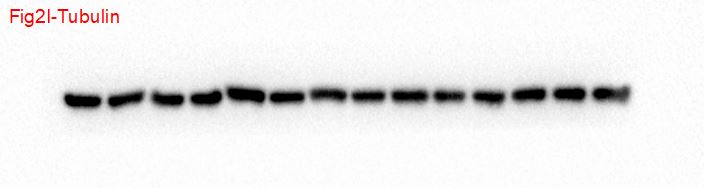

Supplement: Figure 2—source data 3. [file elife-87935-fig2-data3.zip › Figure 2-Source data 4/fig2-C/Fig2I-tubulin.JPG]

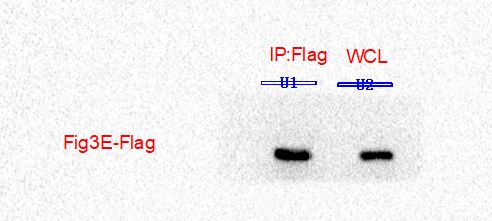

Supplement: Figure 3—source data 3. [file elife-87935-fig3-data3.zip › Figure 3-Source data 6/Fig3-E/Fig3E-Flag.JPG]

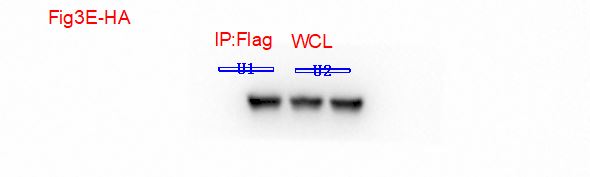

Supplement: Figure 3—source data 3. [file elife-87935-fig3-data3.zip › Figure 3-Source data 6/Fig3-E/Fig3E-HA.JPG]

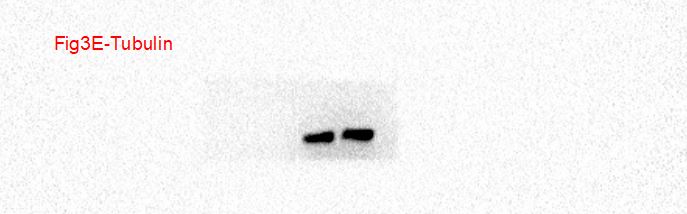

Supplement: Figure 3—source data 3. [file elife-87935-fig3-data3.zip › Figure 3-Source data 6/Fig3-E/Fig3E-Tubulin.JPG]

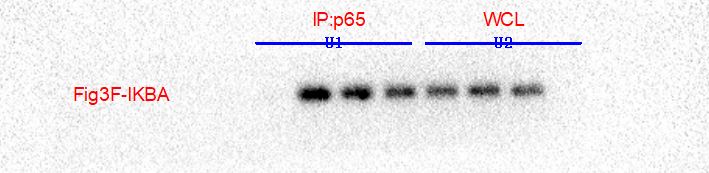

Supplement: Figure 3—source data 3. [file elife-87935-fig3-data3.zip › Figure 3-Source data 6/Fig3-F/Fig3F-IKBA.JPG]

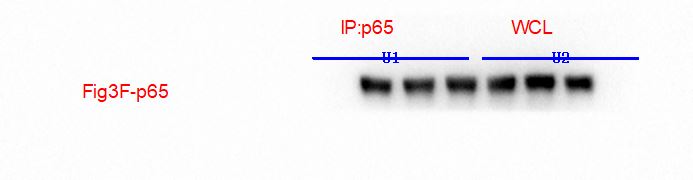

Supplement: Figure 3—source data 3. [file elife-87935-fig3-data3.zip › Figure 3-Source data 6/Fig3-F/Fig3F-p65.JPG]

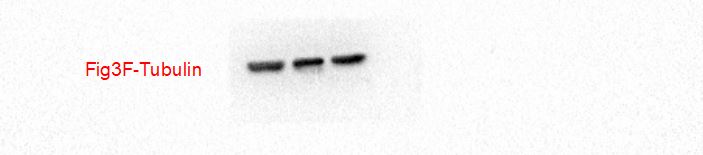

Supplement: Figure 3—source data 3. [file elife-87935-fig3-data3.zip › Figure 3-Source data 6/Fig3-F/Fig3F-Tubulin.JPG]

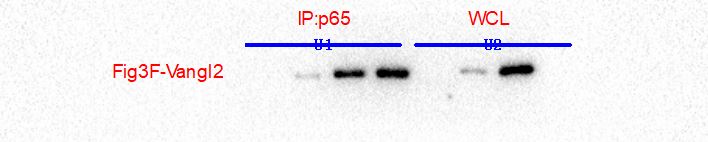

Supplement: Figure 3—source data 3. [file elife-87935-fig3-data3.zip › Figure 3-Source data 6/Fig3-F/Fig3F-Vangl2.JPG]

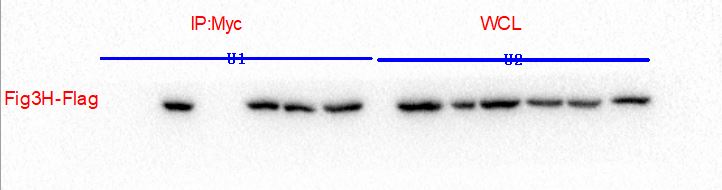

Supplement: Figure 3—source data 3. [file elife-87935-fig3-data3.zip › Figure 3-Source data 6/Fig3-H/Fig3H-Flag.JPG]

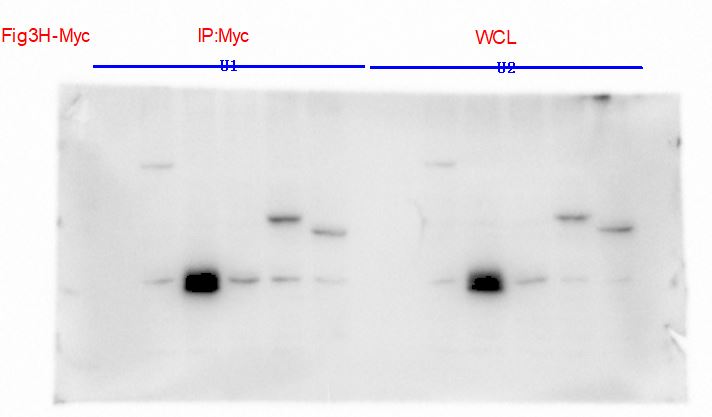

Supplement: Figure 3—source data 3. [file elife-87935-fig3-data3.zip › Figure 3-Source data 6/Fig3-H/Fig3H-myc.JPG]

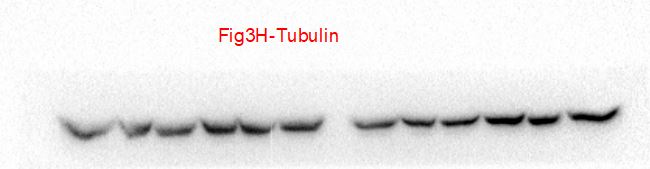

Supplement: Figure 3—source data 3. [file elife-87935-fig3-data3.zip › Figure 3-Source data 6/Fig3-H/Fig3H-tubulin.JPG]

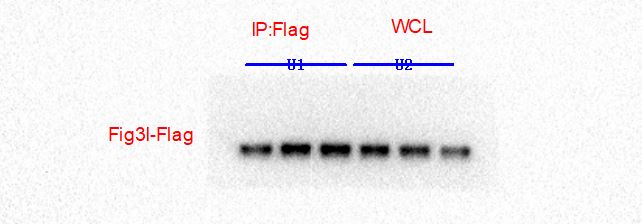

Supplement: Figure 3—source data 3. [file elife-87935-fig3-data3.zip › Figure 3-Source data 6/Fig3-I/Fig3I-Flag.JPG]

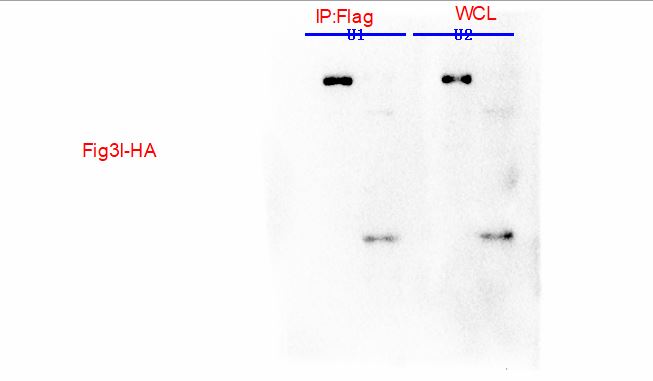

Supplement: Figure 3—source data 3. [file elife-87935-fig3-data3.zip › Figure 3-Source data 6/Fig3-I/Fig3I-HA.JPG]

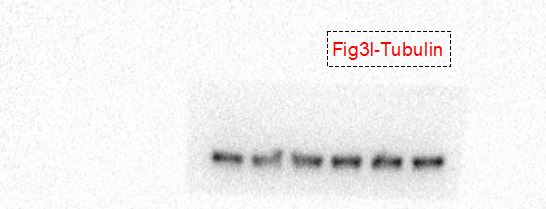

Supplement: Figure 3—source data 3. [file elife-87935-fig3-data3.zip › Figure 3-Source data 6/Fig3-I/Fig3I-Tubulin.JPG]

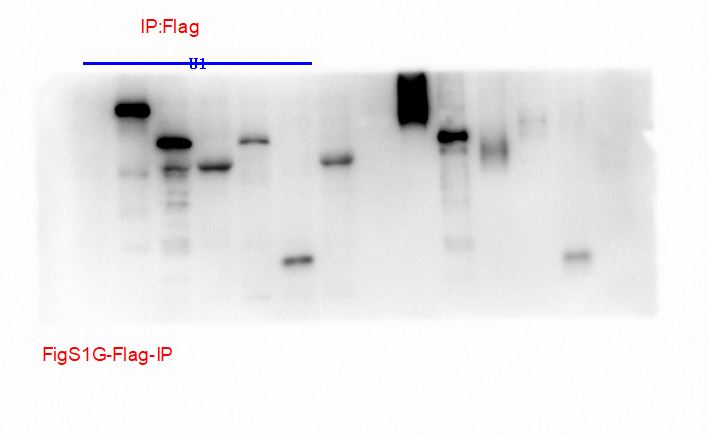

Supplement: Figure 3—source data 3. [file elife-87935-fig3-data3.zip › Figure 3-Source data 6/FigS3-G/FigS3G-Flag-IP.JPG]

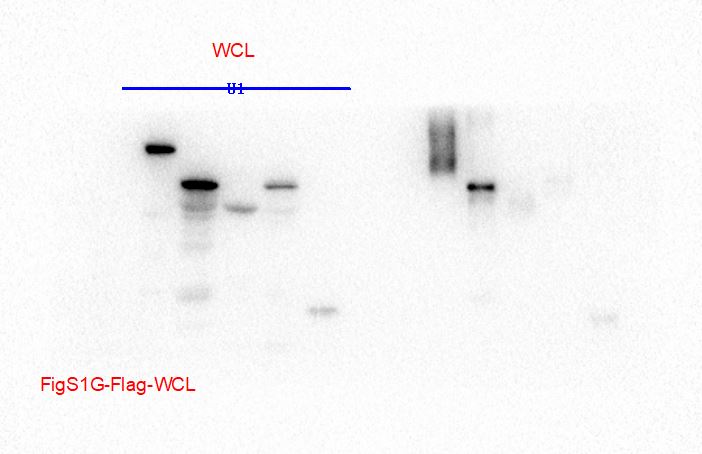

Supplement: Figure 3—source data 3. [file elife-87935-fig3-data3.zip › Figure 3-Source data 6/FigS3-G/FigS3G-Flag-WCL.JPG]

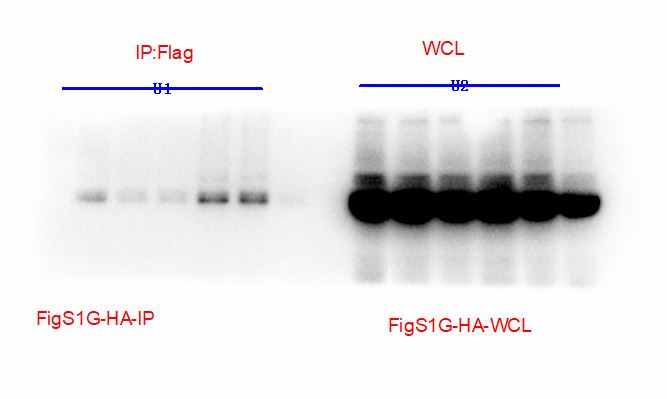

Supplement: Figure 3—source data 3. [file elife-87935-fig3-data3.zip › Figure 3-Source data 6/FigS3-G/FigS3G-HA-ip.JPG]

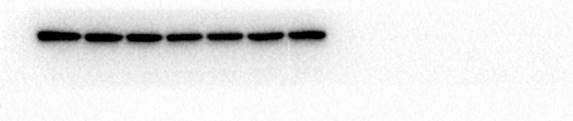

Supplement: Figure 3—source data 3. [file elife-87935-fig3-data3.zip › Figure 3-Source data 6/FigS3-G/FigS3G-HA-WCL.JPG]

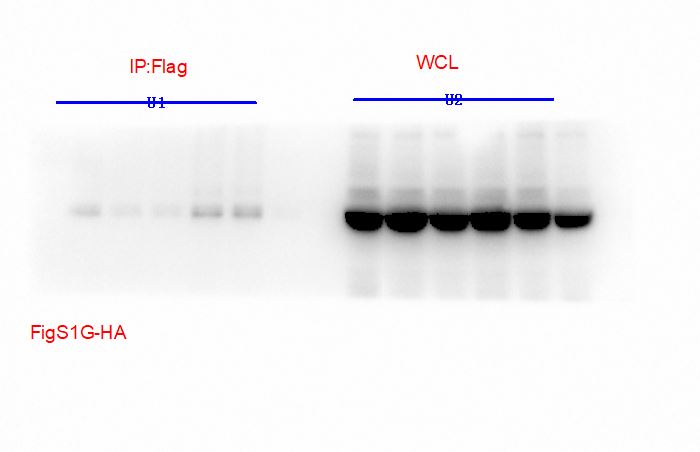

Supplement: Figure 3—source data 3. [file elife-87935-fig3-data3.zip › Figure 3-Source data 6/FigS3-G/FigS3G-Tubulin-WCL.JPG]

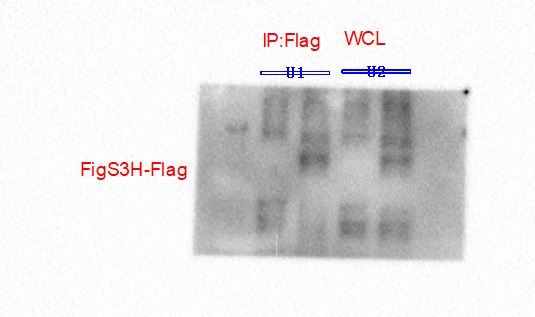

Supplement: Figure 3—source data 3. [file elife-87935-fig3-data3.zip › Figure 3-Source data 6/FigS3-H/FigS3H-Flag.JPG]

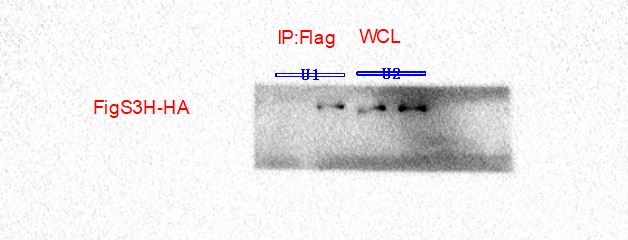

Supplement: Figure 3—source data 3. [file elife-87935-fig3-data3.zip › Figure 3-Source data 6/FigS3-H/FigS3H-HA.JPG]

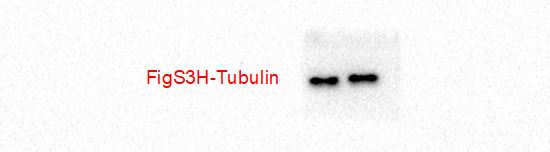

Supplement: Figure 3—source data 3. [file elife-87935-fig3-data3.zip › Figure 3-Source data 6/FigS3-H/FigS3H-Tubulin.JPG]

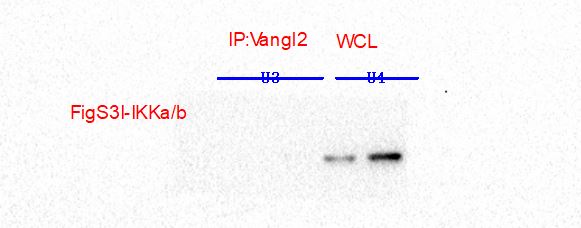

Supplement: Figure 3—source data 3. [file elife-87935-fig3-data3.zip › Figure 3-Source data 6/FigS3-I/FigS3I-IKKab.JPG]

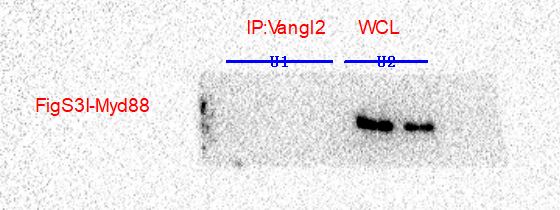

Supplement: Figure 3—source data 3. [file elife-87935-fig3-data3.zip › Figure 3-Source data 6/FigS3-I/FigS3I-Myd88.JPG]

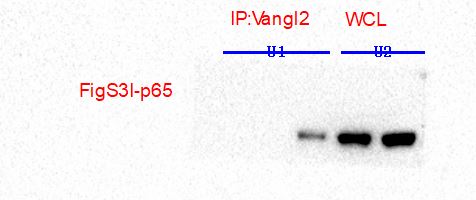

Supplement: Figure 3—source data 3. [file elife-87935-fig3-data3.zip › Figure 3-Source data 6/FigS3-I/FigS3I-p65.JPG]

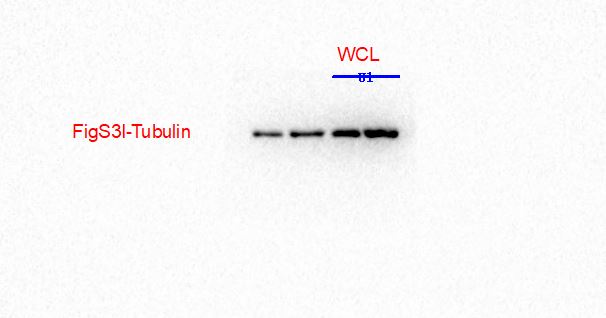

Supplement: Figure 3—source data 3. [file elife-87935-fig3-data3.zip › Figure 3-Source data 6/FigS3-I/FigS3I-Tubulin.JPG]

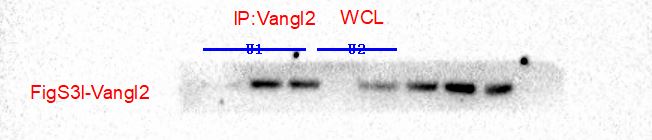

Supplement: Figure 3—source data 3. [file elife-87935-fig3-data3.zip › Figure 3-Source data 6/FigS3-I/FigS3I-Vangl2.JPG]

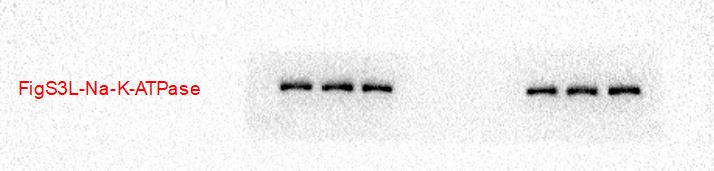

Supplement: Figure 3—source data 3. [file elife-87935-fig3-data3.zip › Figure 3-Source data 6/FigS3-L/FigS3L-Na-K-ATPase.JPG]

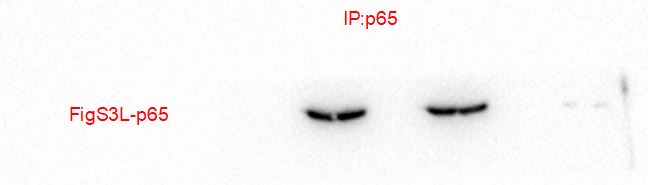

Supplement: Figure 3—source data 3. [file elife-87935-fig3-data3.zip › Figure 3-Source data 6/FigS3-L/FigS3L-p65-IP.JPG]

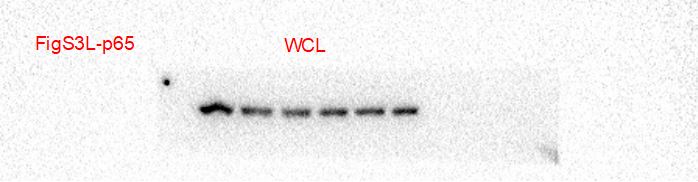

Supplement: Figure 3—source data 3. [file elife-87935-fig3-data3.zip › Figure 3-Source data 6/FigS3-L/FigS3L-p65-WCL.JPG]

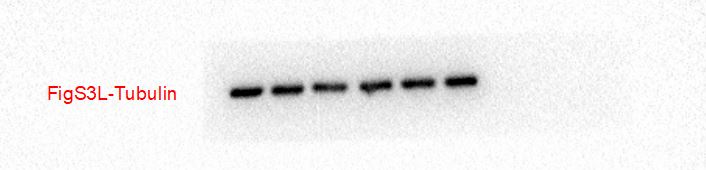

Supplement: Figure 3—source data 3. [file elife-87935-fig3-data3.zip › Figure 3-Source data 6/FigS3-L/FigS3L-Tubulin.JPG]

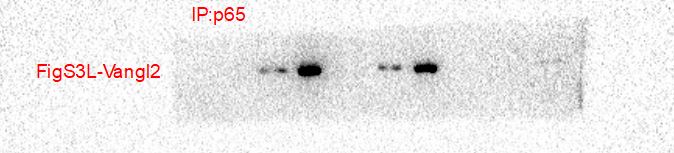

Supplement: Figure 3—source data 3. [file elife-87935-fig3-data3.zip › Figure 3-Source data 6/FigS3-L/FigS3L-Vangl2-IP.JPG]

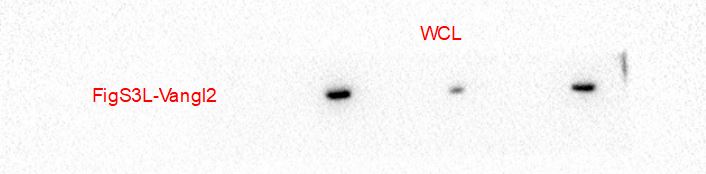

Supplement: Figure 3—source data 3. [file elife-87935-fig3-data3.zip › Figure 3-Source data 6/FigS3-L/FigS3L-Vangl2-WCL.JPG]

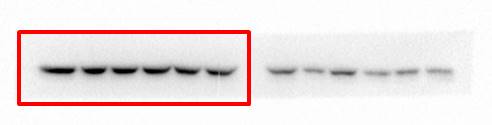

Supplement: Figure 3—source data 3. [file elife-87935-fig3-data3.zip › Figure 3-Source data 6/FigS3-M/FigS3M-Flag.jpg]

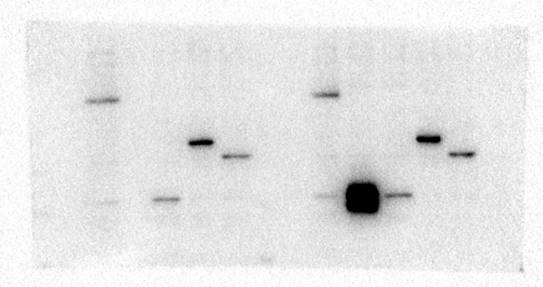

Supplement: Figure 3—source data 3. [file elife-87935-fig3-data3.zip › Figure 3-Source data 6/FigS3-M/FigS3M-Myc.jpg]

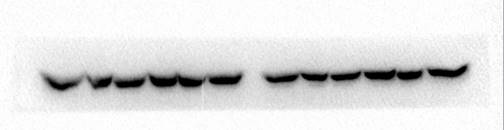

Supplement: Figure 3—source data 3. [file elife-87935-fig3-data3.zip › Figure 3-Source data 6/FigS3-M/FigS3M-Tubulin.jpg]

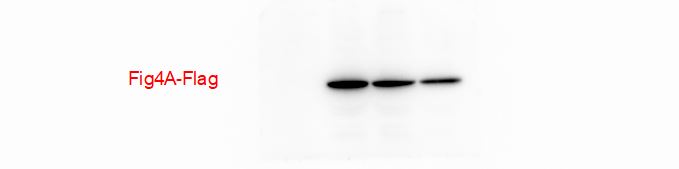

Supplement: Figure 4—source data 3. [file elife-87935-fig4-data3.zip › Figure 4-Source data 8/Fig4-A/Fig4A-Flag.JPG]

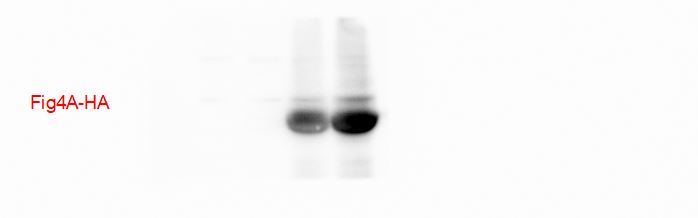

Supplement: Figure 4—source data 3. [file elife-87935-fig4-data3.zip › Figure 4-Source data 8/Fig4-A/Fig4A-HA.JPG]

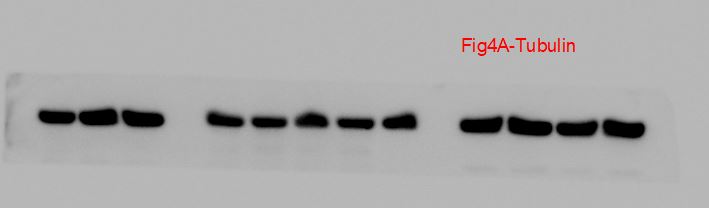

Supplement: Figure 4—source data 3. [file elife-87935-fig4-data3.zip › Figure 4-Source data 8/Fig4-A/Fig4A-Tubulin.JPG]

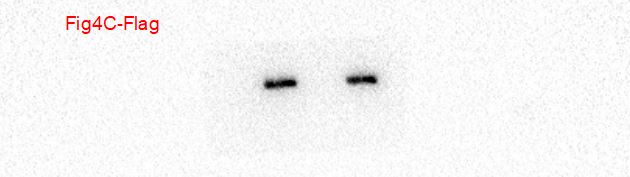

Supplement: Figure 4—source data 3. [file elife-87935-fig4-data3.zip › Figure 4-Source data 8/Fig4-C/Fig4C-Flag.JPG]

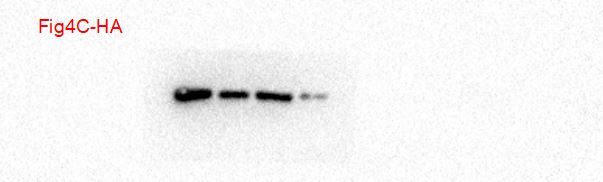

Supplement: Figure 4—source data 3. [file elife-87935-fig4-data3.zip › Figure 4-Source data 8/Fig4-C/Fig4C-HA.JPG]

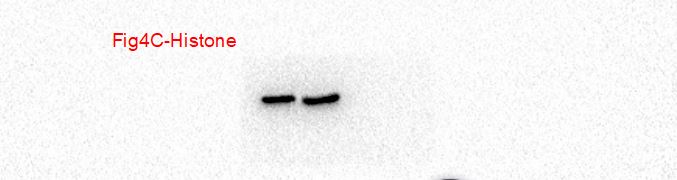

Supplement: Figure 4—source data 3. [file elife-87935-fig4-data3.zip › Figure 4-Source data 8/Fig4-C/Fig4C-Histone.JPG]

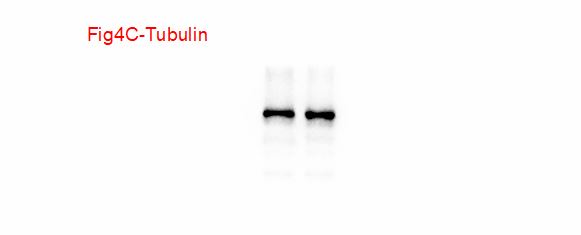

Supplement: Figure 4—source data 3. [file elife-87935-fig4-data3.zip › Figure 4-Source data 8/Fig4-C/Fig4C-Tubulin.JPG]

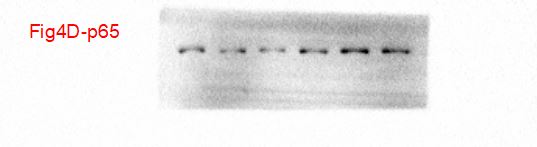

Supplement: Figure 4—source data 3. [file elife-87935-fig4-data3.zip › Figure 4-Source data 8/Fig4-D/Fig4D-p65.JPG]

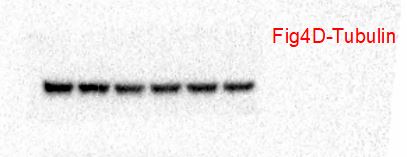

Supplement: Figure 4—source data 3. [file elife-87935-fig4-data3.zip › Figure 4-Source data 8/Fig4-D/Fig4D-Tubulin.JPG]

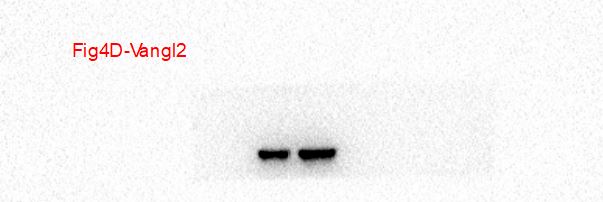

Supplement: Figure 4—source data 3. [file elife-87935-fig4-data3.zip › Figure 4-Source data 8/Fig4-D/Fig4D-Vangl2.JPG]

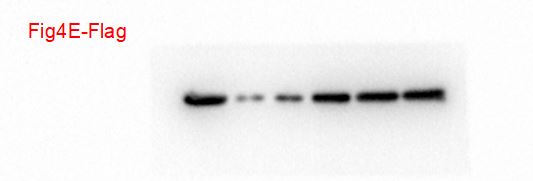

Supplement: Figure 4—source data 3. [file elife-87935-fig4-data3.zip › Figure 4-Source data 8/Fig4-E/Fig4E-Flag.JPG]

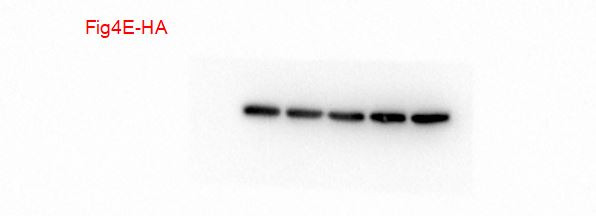

Supplement: Figure 4—source data 3. [file elife-87935-fig4-data3.zip › Figure 4-Source data 8/Fig4-E/Fig4E-HA.JPG]

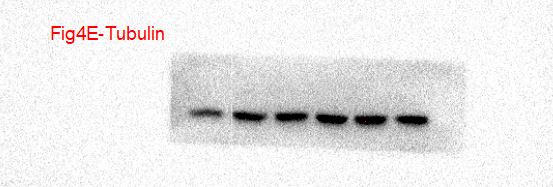

Supplement: Figure 4—source data 3. [file elife-87935-fig4-data3.zip › Figure 4-Source data 8/Fig4-E/Fig4E-Tubulin.JPG]

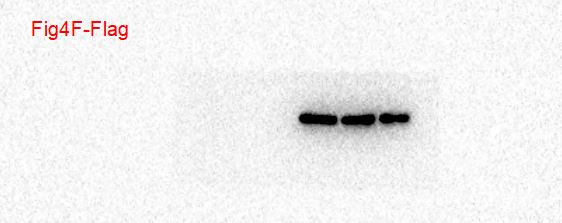

Supplement: Figure 4—source data 3. [file elife-87935-fig4-data3.zip › Figure 4-Source data 8/Fig4-F/Fig4F-Flag.JPG]

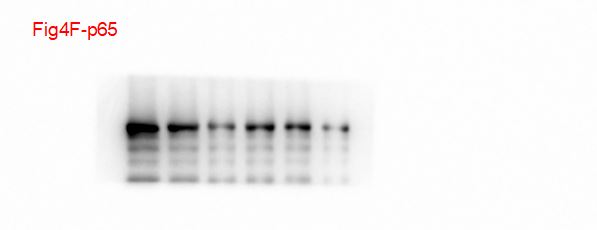

Supplement: Figure 4—source data 3. [file elife-87935-fig4-data3.zip › Figure 4-Source data 8/Fig4-F/Fig4F-p65.JPG]

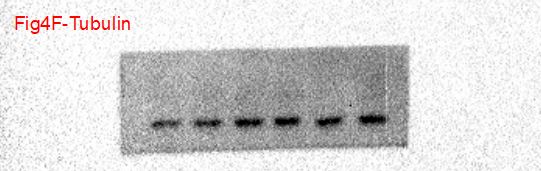

Supplement: Figure 4—source data 3. [file elife-87935-fig4-data3.zip › Figure 4-Source data 8/Fig4-F/Fig4F-Tubulin.JPG]

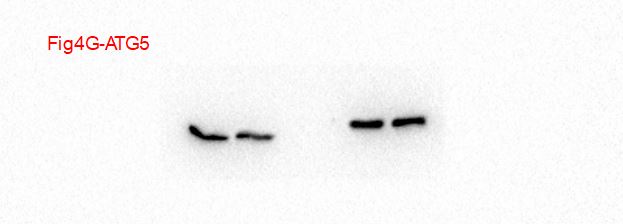

Supplement: Figure 4—source data 3. [file elife-87935-fig4-data3.zip › Figure 4-Source data 8/Fig4-G/Fig4G-ATG5.JPG]

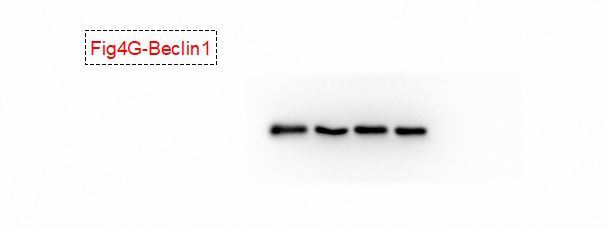

Supplement: Figure 4—source data 3. [file elife-87935-fig4-data3.zip › Figure 4-Source data 8/Fig4-G/Fig4G-Beclin1.JPG]

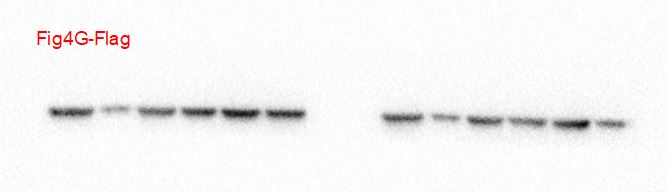

Supplement: Figure 4—source data 3. [file elife-87935-fig4-data3.zip › Figure 4-Source data 8/Fig4-G/Fig4G-Flag.JPG]

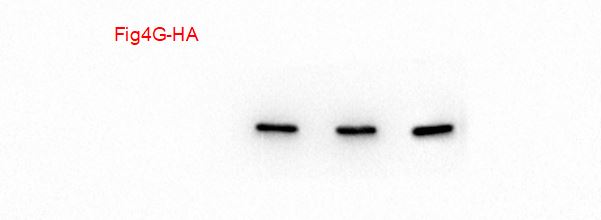

Supplement: Figure 4—source data 3. [file elife-87935-fig4-data3.zip › Figure 4-Source data 8/Fig4-G/Fig4G-ha.JPG]

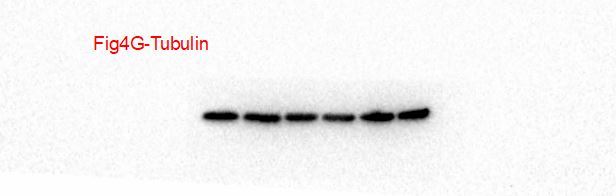

Supplement: Figure 4—source data 3. [file elife-87935-fig4-data3.zip › Figure 4-Source data 8/Fig4-G/Fig4G-Tubulin.JPG]

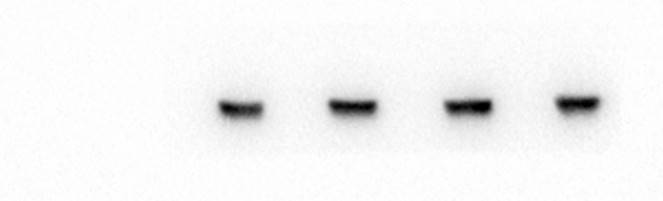

Supplement: Figure 4—source data 3. [file elife-87935-fig4-data3.zip › Figure 4-Source data 8/FigS4-AB/FigS4-AB-Flag.jpg]

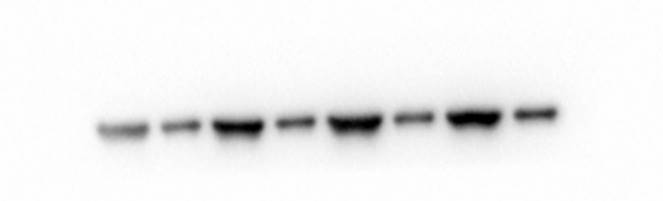

Supplement: Figure 4—source data 3. [file elife-87935-fig4-data3.zip › Figure 4-Source data 8/FigS4-AB/FigS4-AB-HA.jpg]

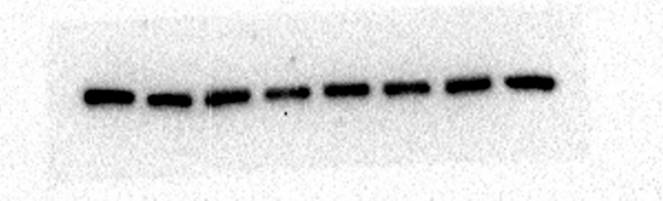

Supplement: Figure 4—source data 3. [file elife-87935-fig4-data3.zip › Figure 4-Source data 8/FigS4-AB/FigS4-AB-Tubulin.jpg]

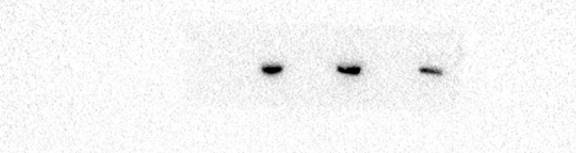

Supplement: Figure 4—source data 3. [file elife-87935-fig4-data3.zip › Figure 4-Source data 8/FigS4-C/FigS4C-Flag.jpg]

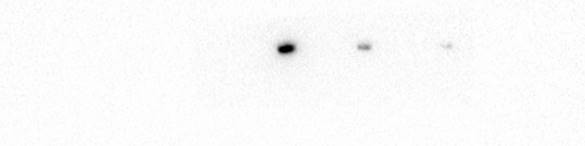

Supplement: Figure 4—source data 3. [file elife-87935-fig4-data3.zip › Figure 4-Source data 8/FigS4-C/FigS4C-HA.jpg]

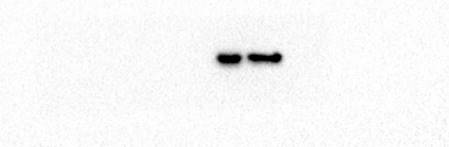

Supplement: Figure 4—source data 3. [file elife-87935-fig4-data3.zip › Figure 4-Source data 8/FigS4-C/FigS4C-Histone.jpg]

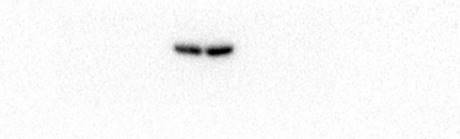

Supplement: Figure 4—source data 3. [file elife-87935-fig4-data3.zip › Figure 4-Source data 8/FigS4-C/FigS4C-Na+K+ATPase.jpg]

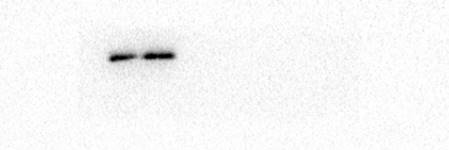

Supplement: Figure 4—source data 3. [file elife-87935-fig4-data3.zip › Figure 4-Source data 8/FigS4-C/FigS4C-Tubulin.jpg]

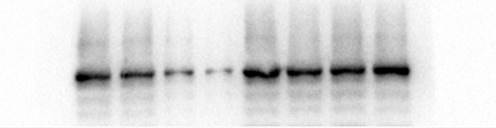

Supplement: Figure 4—source data 3. [file elife-87935-fig4-data3.zip › Figure 4-Source data 8/FigS4-D/FigS4D-p65.jpg]

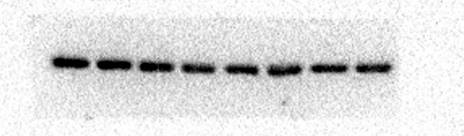

Supplement: Figure 4—source data 3. [file elife-87935-fig4-data3.zip › Figure 4-Source data 8/FigS4-D/FigS4D-Tubulin.jpg]

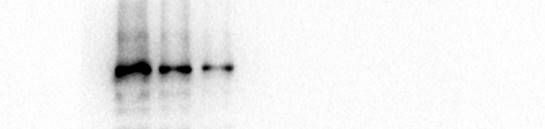

Supplement: Figure 4—source data 3. [file elife-87935-fig4-data3.zip › Figure 4-Source data 8/FigS4-D/FigS4D-Vangl2.jpg]

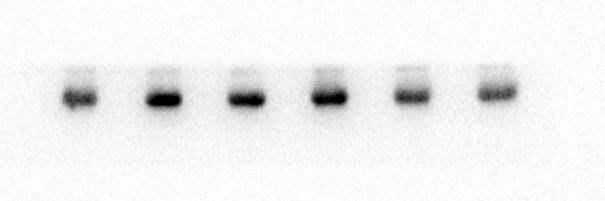

Supplement: Figure 4—source data 3. [file elife-87935-fig4-data3.zip › Figure 4-Source data 8/FigS4-E/FigS4E-Flag.jpg]

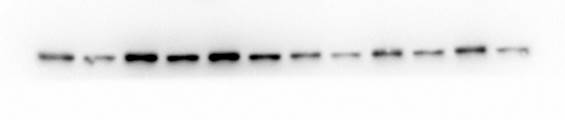

Supplement: Figure 4—source data 3. [file elife-87935-fig4-data3.zip › Figure 4-Source data 8/FigS4-E/FigS4E-HA.jpg]

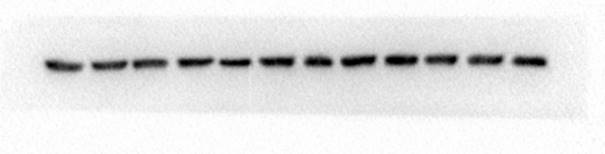

Supplement: Figure 4—source data 3. [file elife-87935-fig4-data3.zip › Figure 4-Source data 8/FigS4-E/FigS4E-Tubulin.jpg]

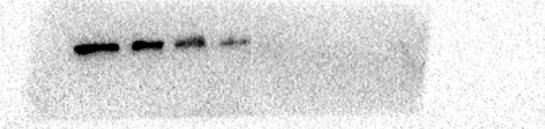

Supplement: Figure 4—source data 3. [file elife-87935-fig4-data3.zip › Figure 4-Source data 8/FigS4-F/FigS4F-ATG5.jpg]

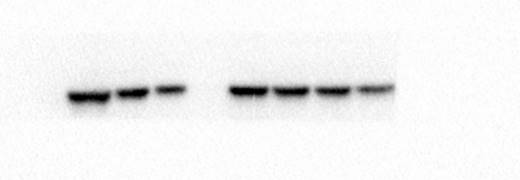

Supplement: Figure 4—source data 3. [file elife-87935-fig4-data3.zip › Figure 4-Source data 8/FigS4-F/FigS4F-p65.jpg]

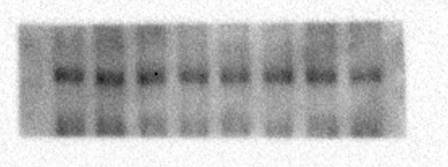

Supplement: Figure 4—source data 3. [file elife-87935-fig4-data3.zip › Figure 4-Source data 8/FigS4-F/FigS4F-Tubulin.jpg]

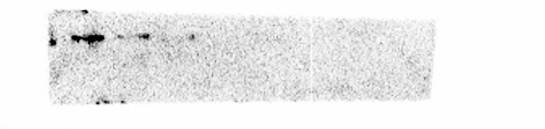

Supplement: Figure 4—source data 3. [file elife-87935-fig4-data3.zip › Figure 4-Source data 8/FigS4-G/FigS4G-Beclin1.jpg]
