# Supplementary material for: Vangl2 suppresses NF-κB signaling and ameliorates sepsis by targeting p65 for NDP52-mediated autophagic degradation
Source: eLife. 2024 Sep 13;12:RP87935. doi: 10.7554/eLife.87935 (PMC11398866; doi:10.7554/eLife.87935)

Figure 3

E

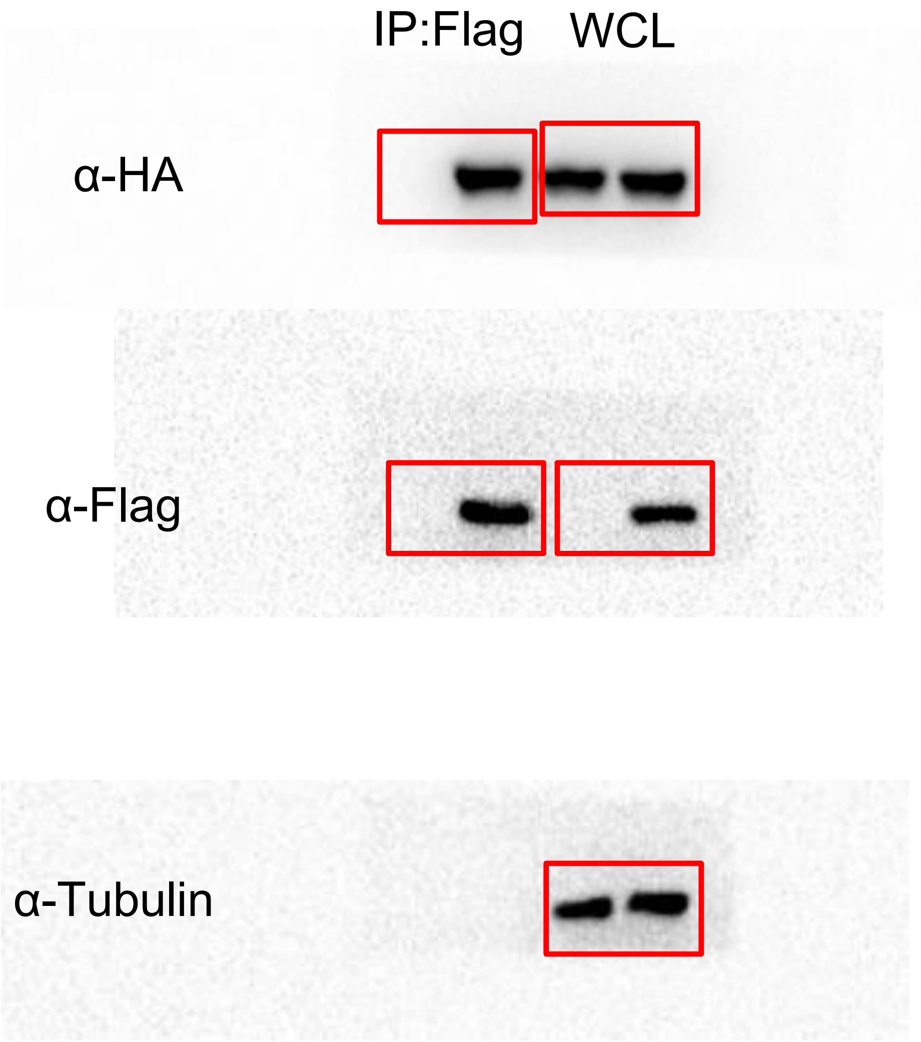

Figure 3

E

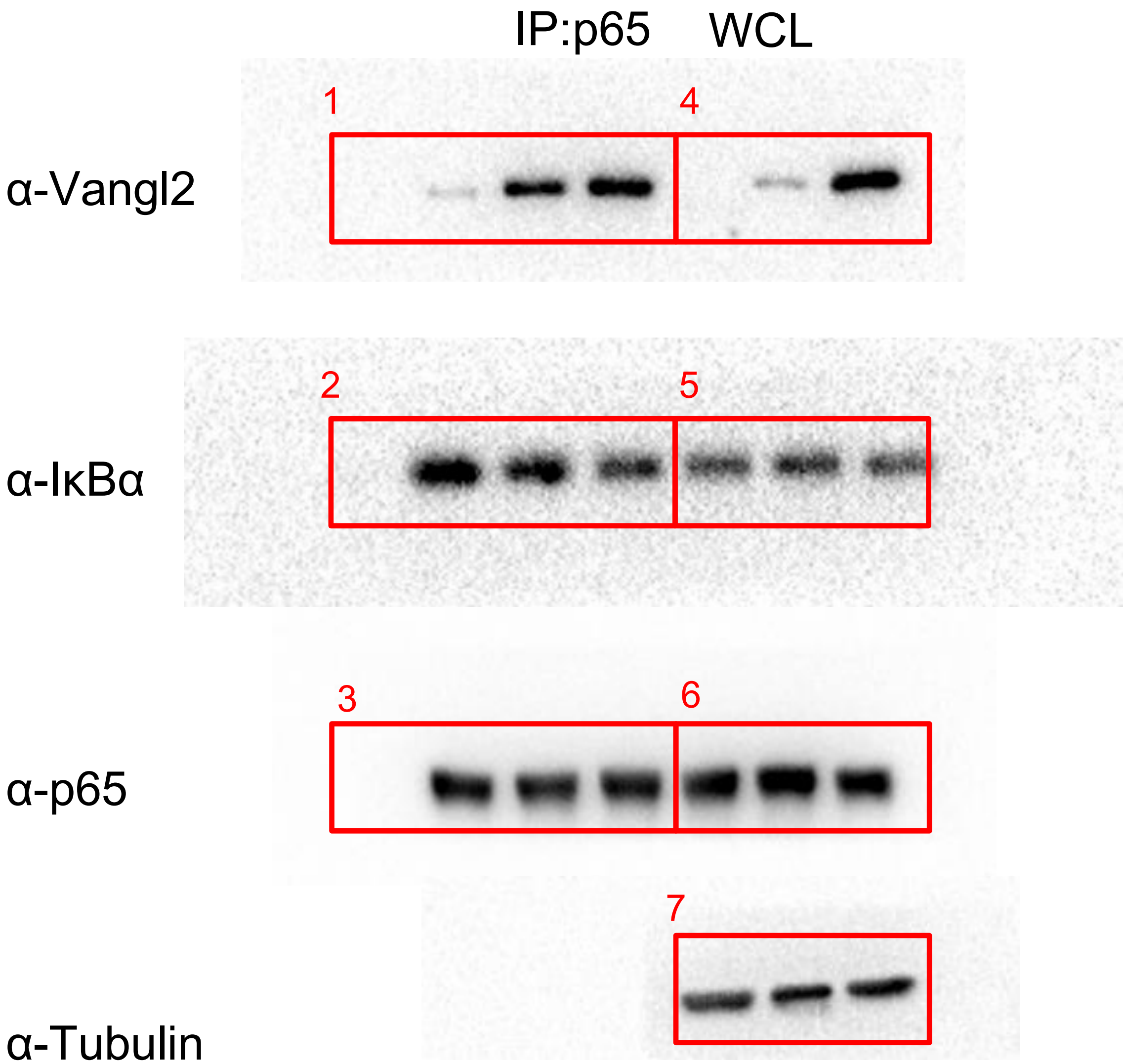

Figure 3

H

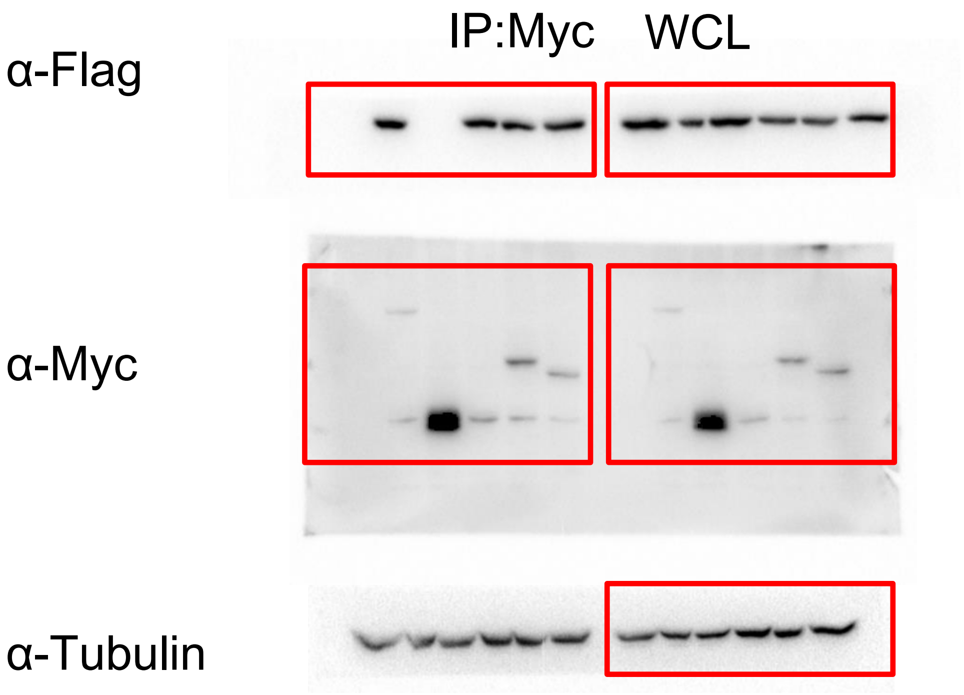

Figure 3

I

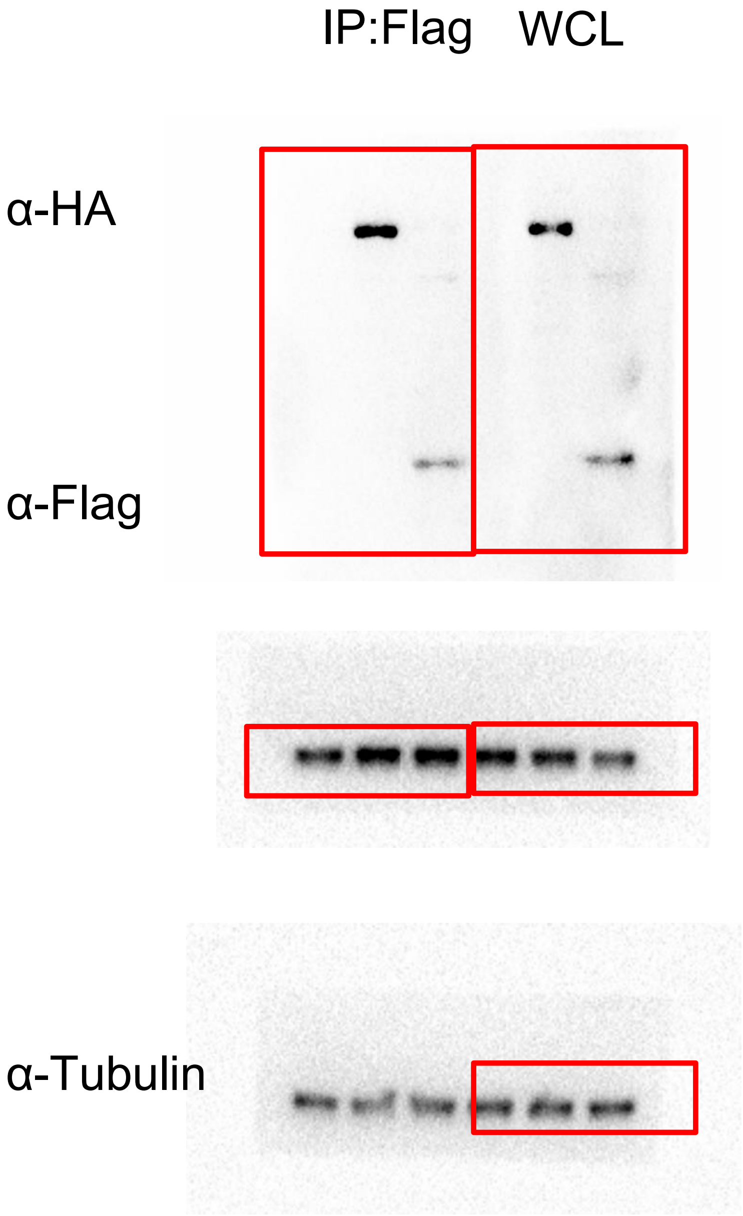

Figure 3-figure supplement 3

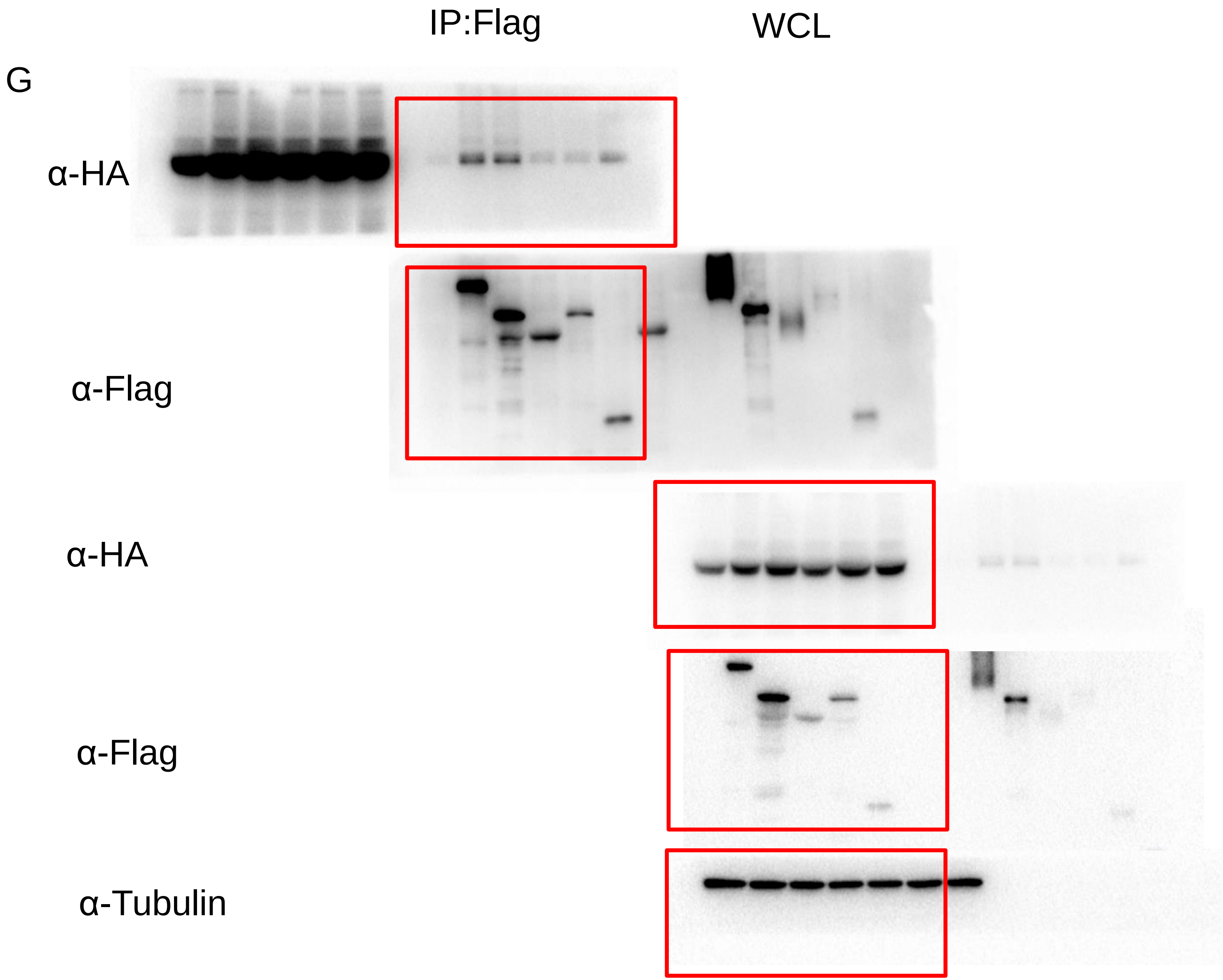

Figure 3-figure supplement 3

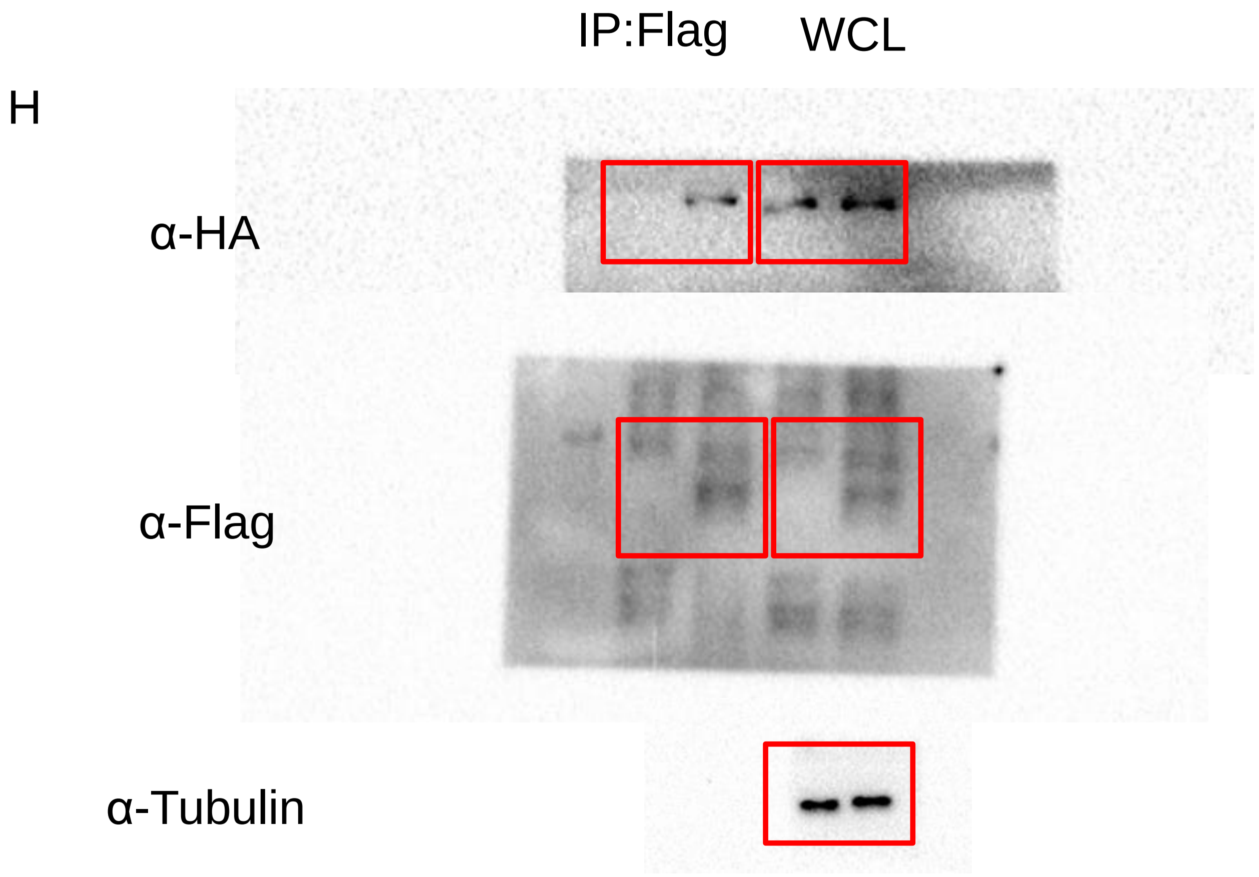

Figure 3-figure supplement 3

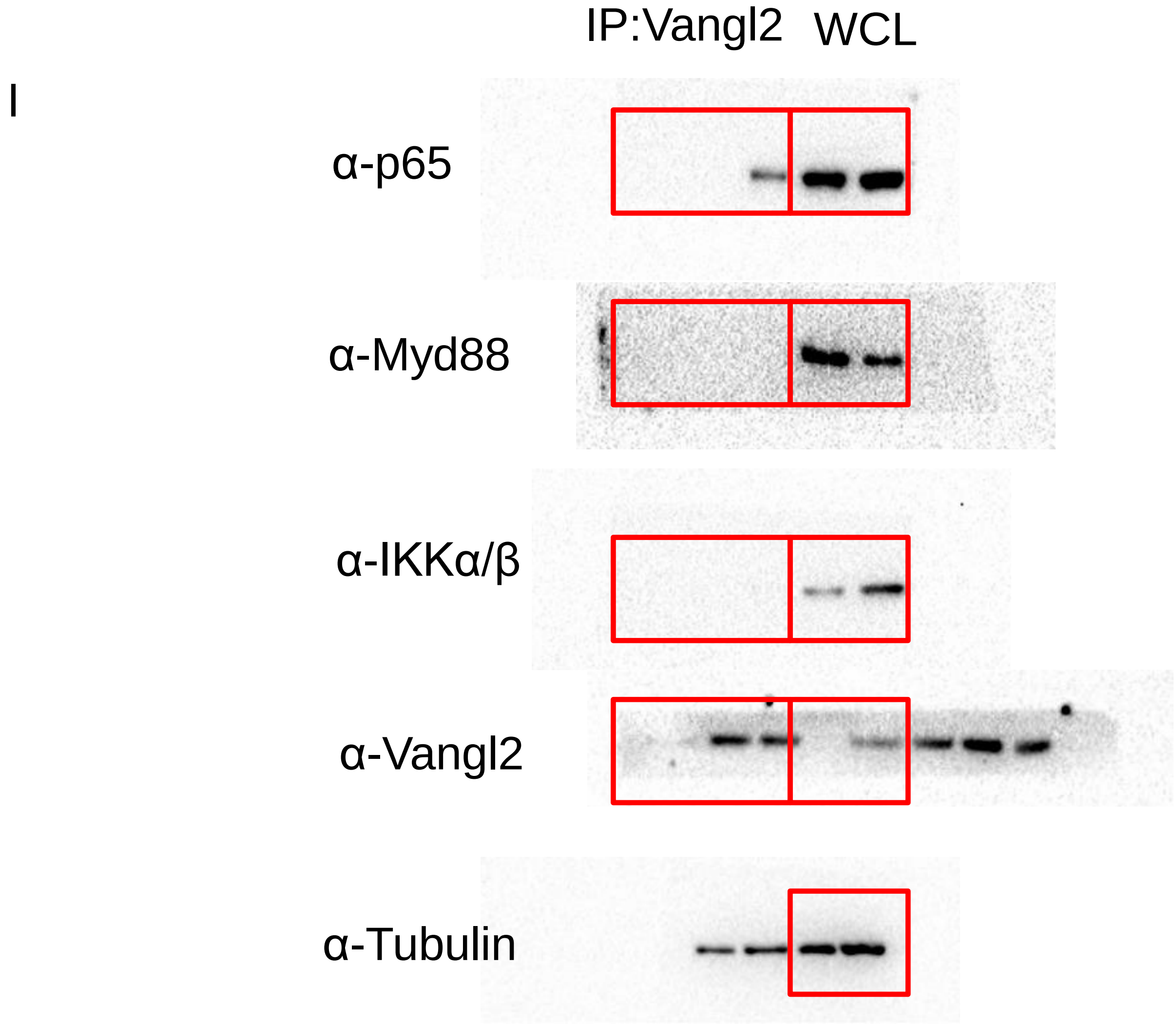

Figure 3-figure supplement 3

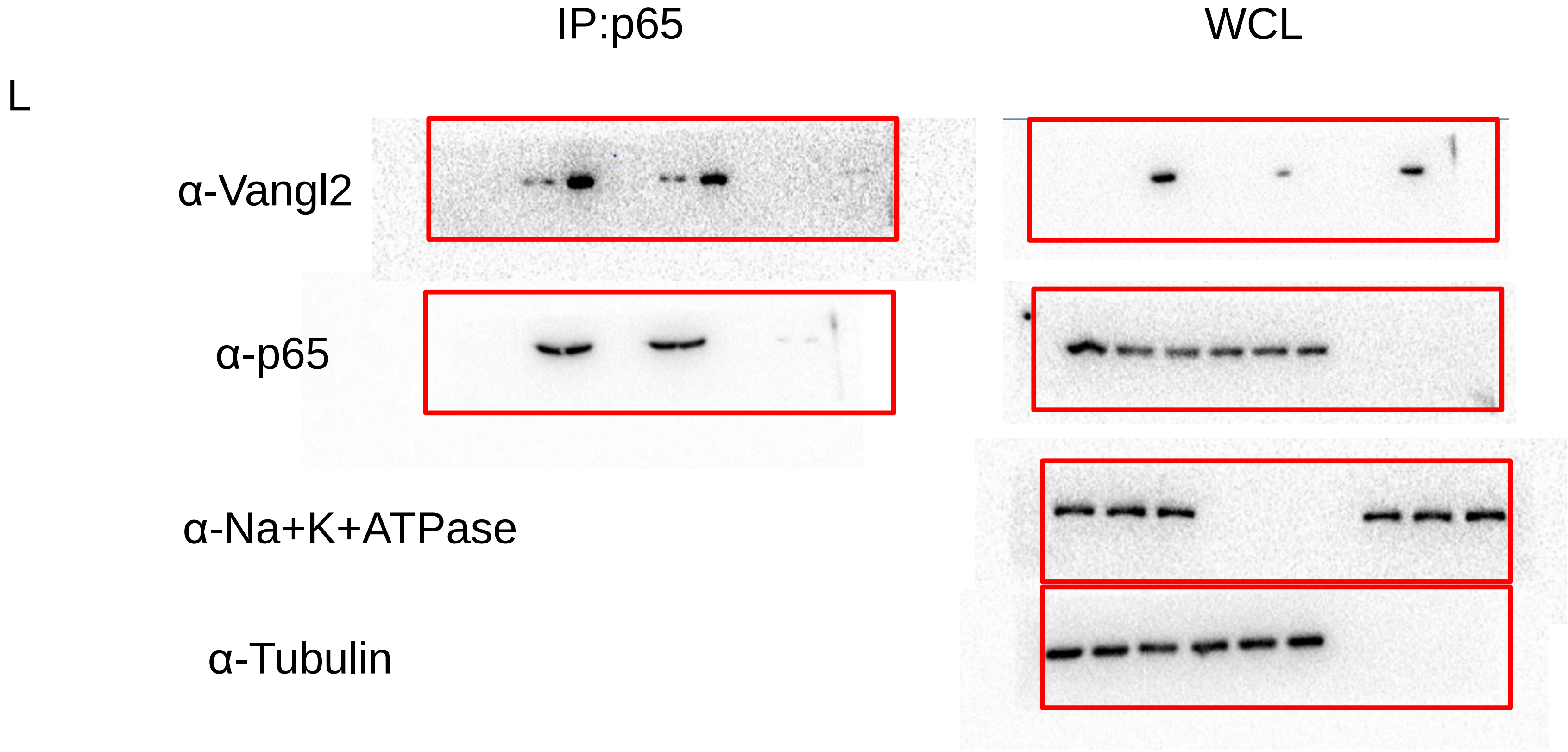

Figure 3-figure supplement 3

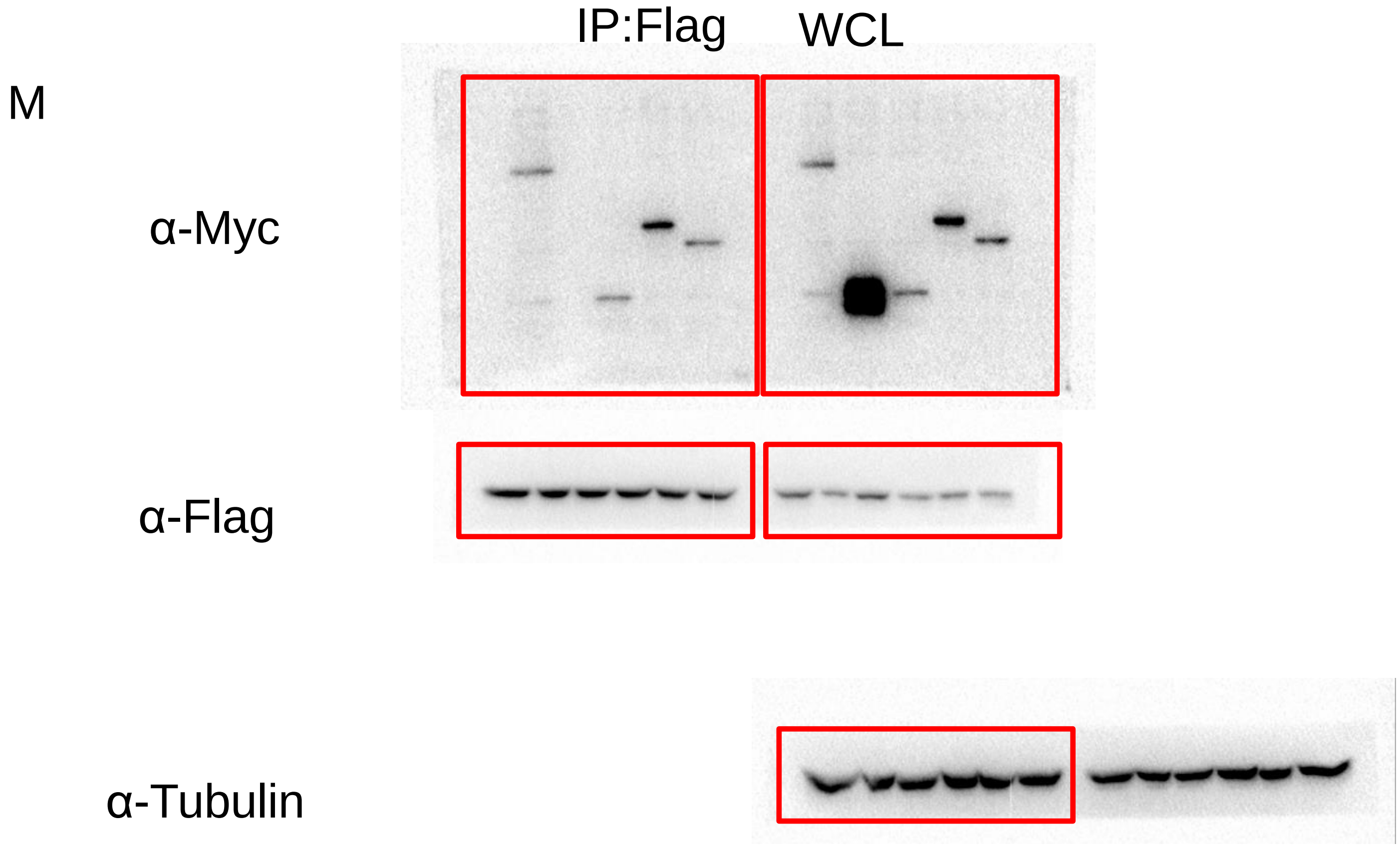

Supplement: Figure 3—source data 1. [file elife-87935-fig3-data1.pdf]
