# Supplementary material for: Vangl2 suppresses NF-κB signaling and ameliorates sepsis by targeting p65 for NDP52-mediated autophagic degradation
Source: eLife. 2024 Sep 13;12:RP87935. doi: 10.7554/eLife.87935 (PMC11398866; doi:10.7554/eLife.87935)

Figure 4

I

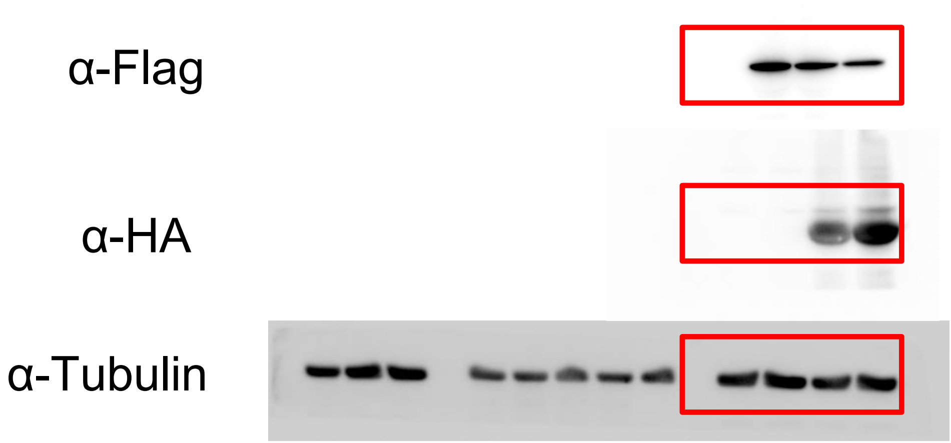

Figure 4

I

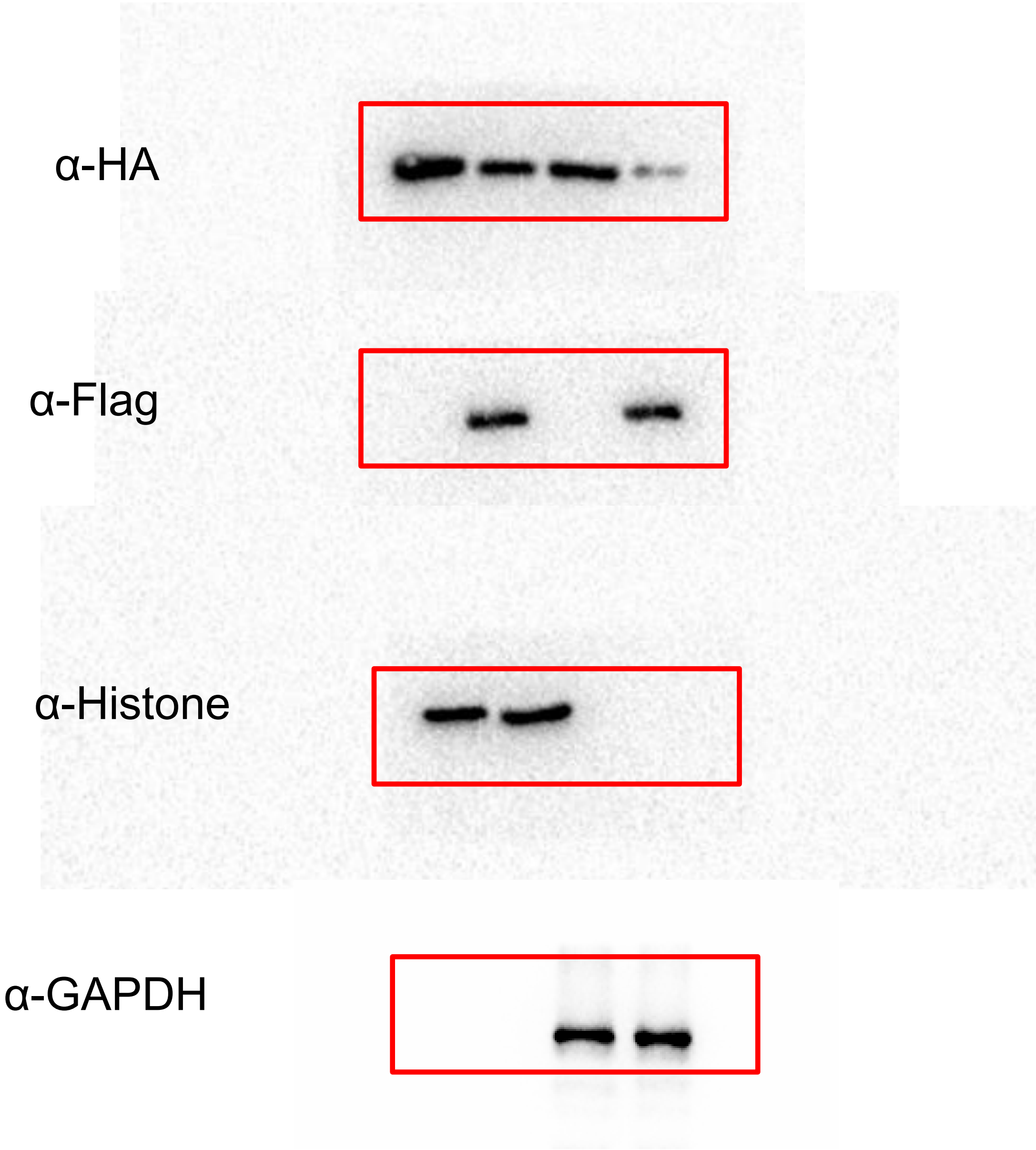

Figure 4

D

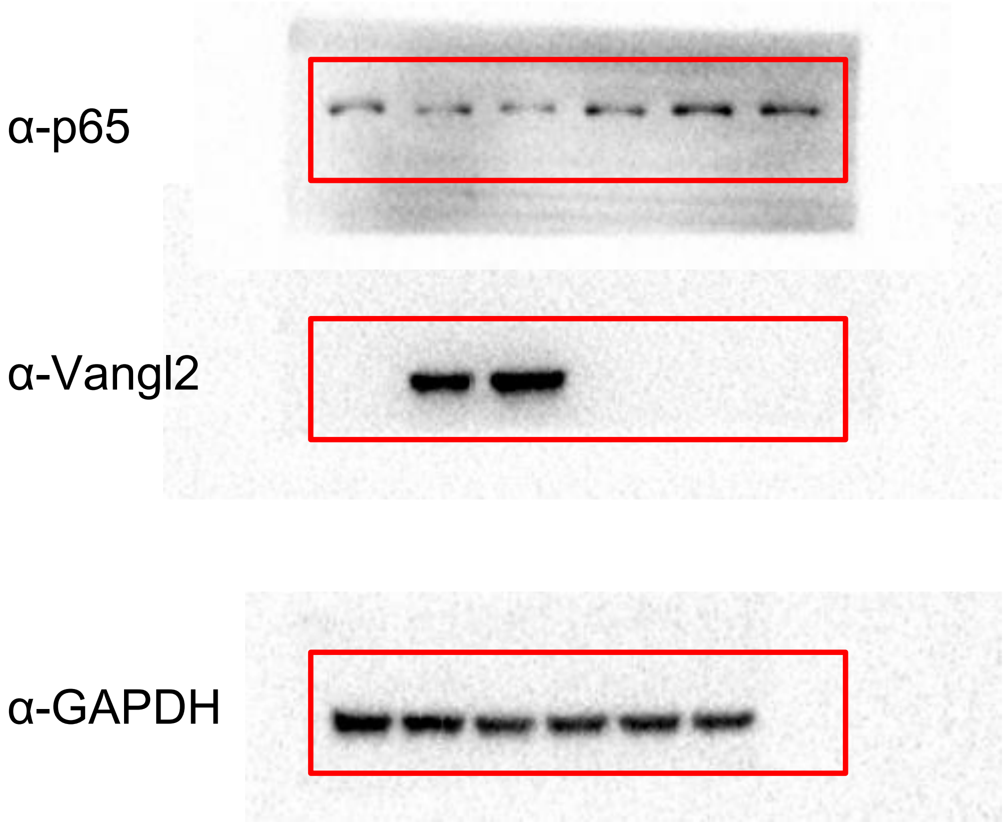

Figure 4

E

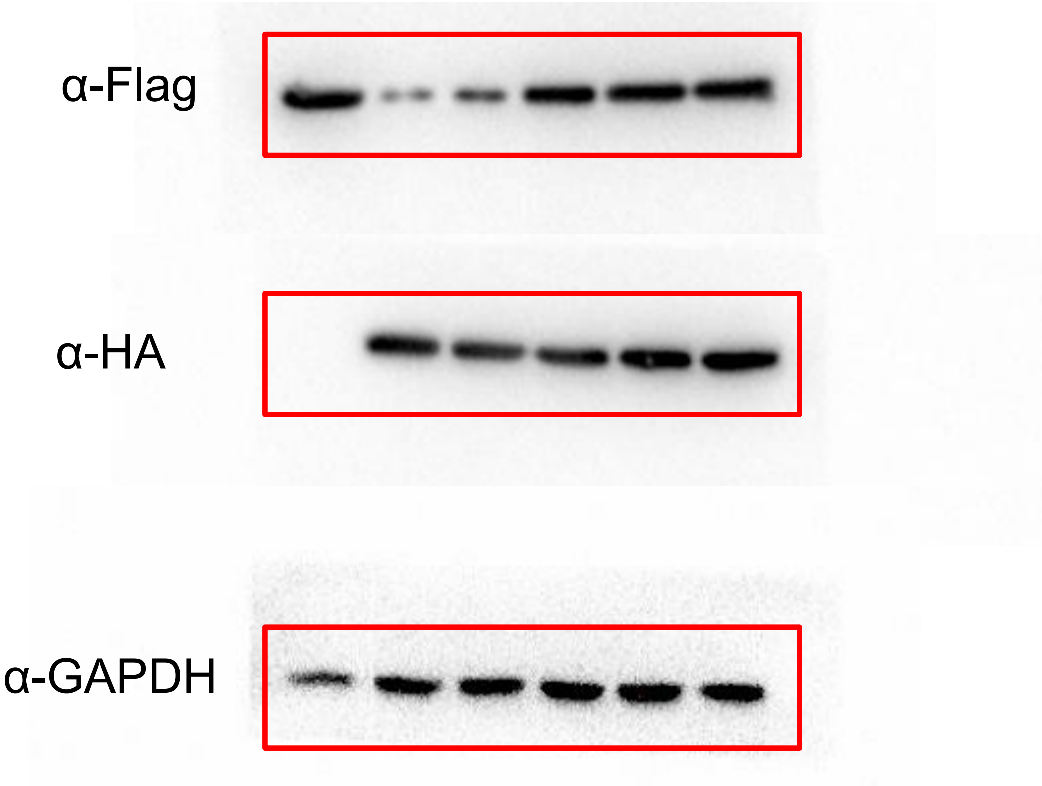

Figure 4

F

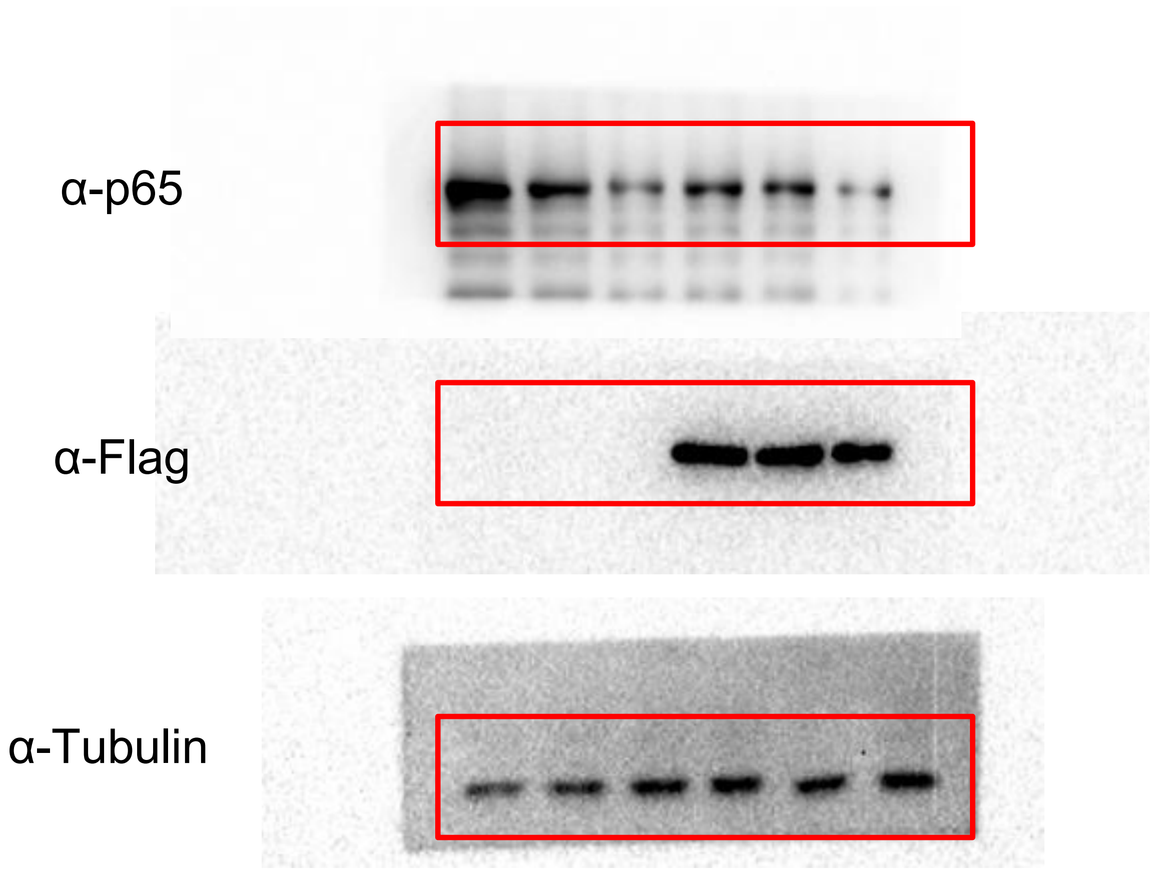

Figure 4

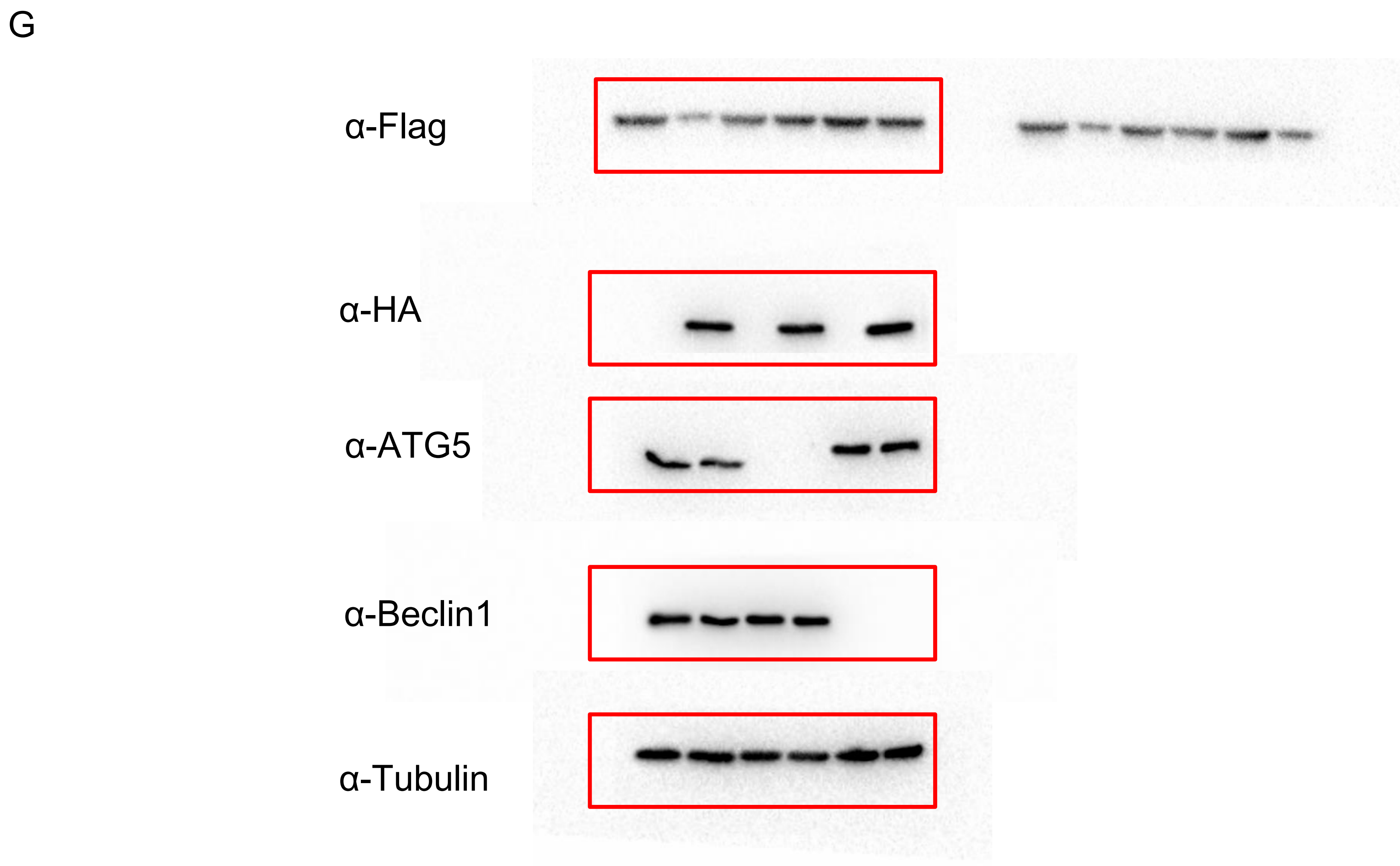

Figure 4-figure supplement 4

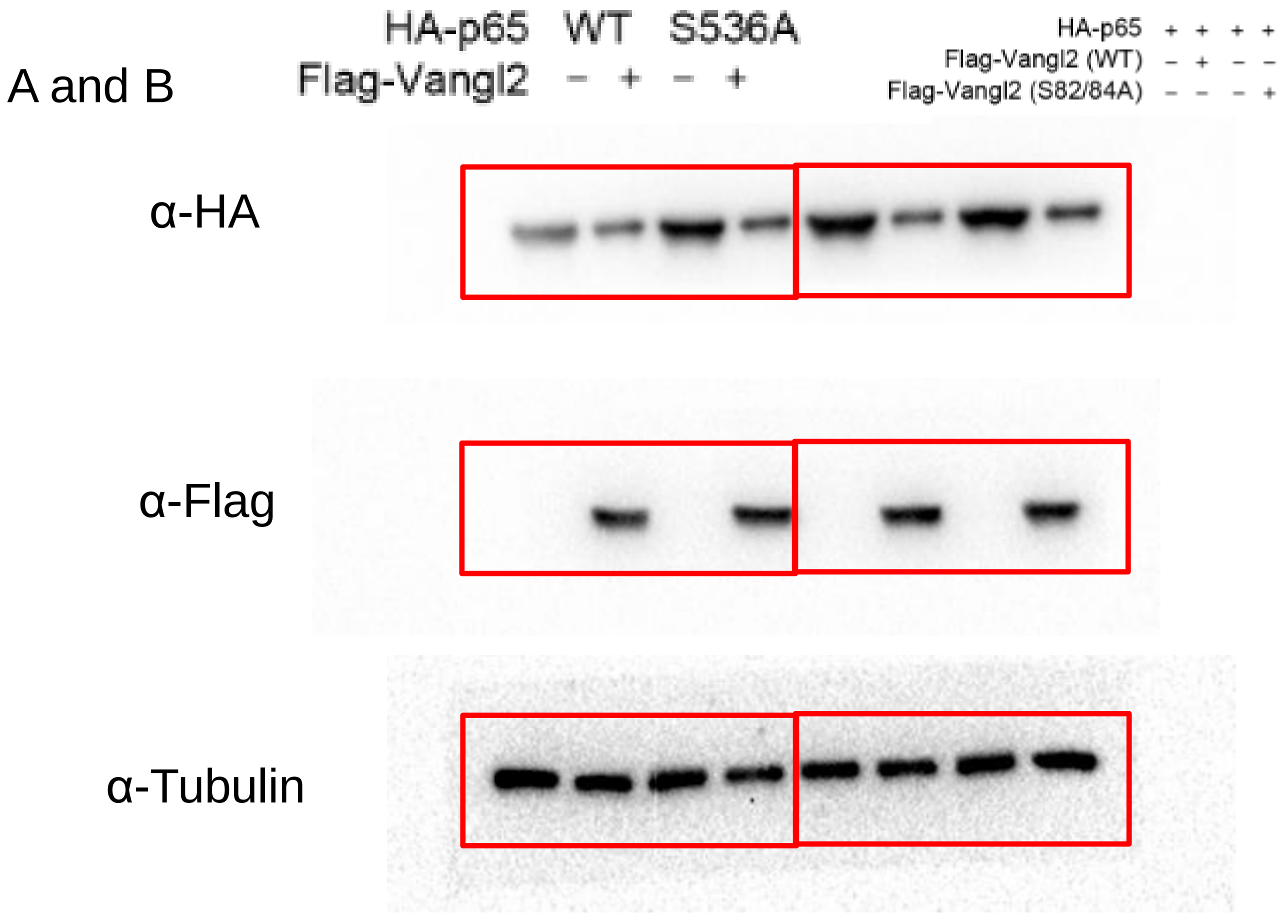

Figure 4-figure supplement 4

C

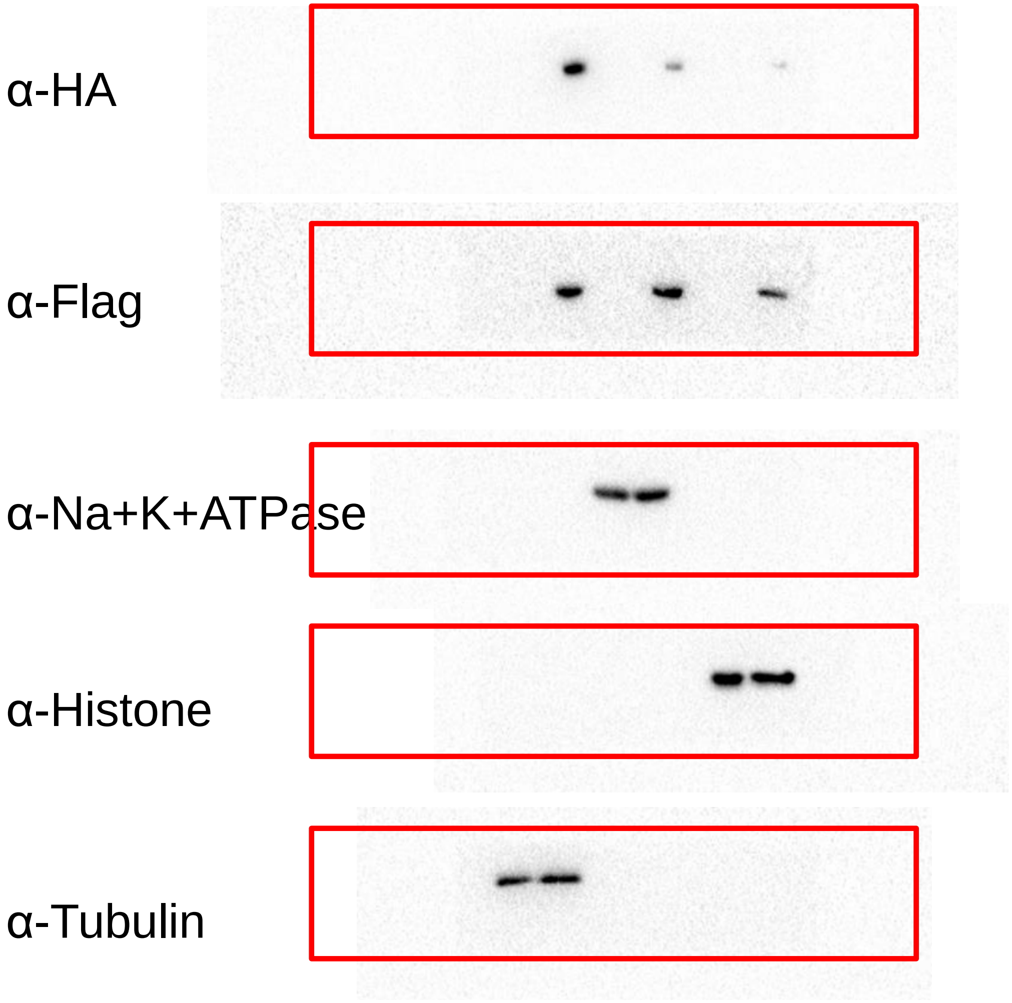

Figure 4-figure supplement 4

D

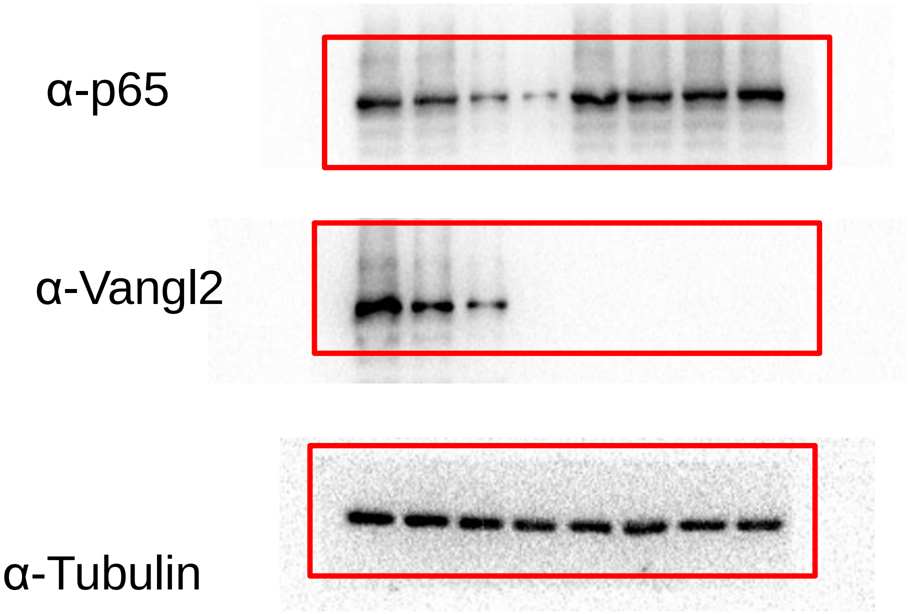

Figure 4-figure supplement 4

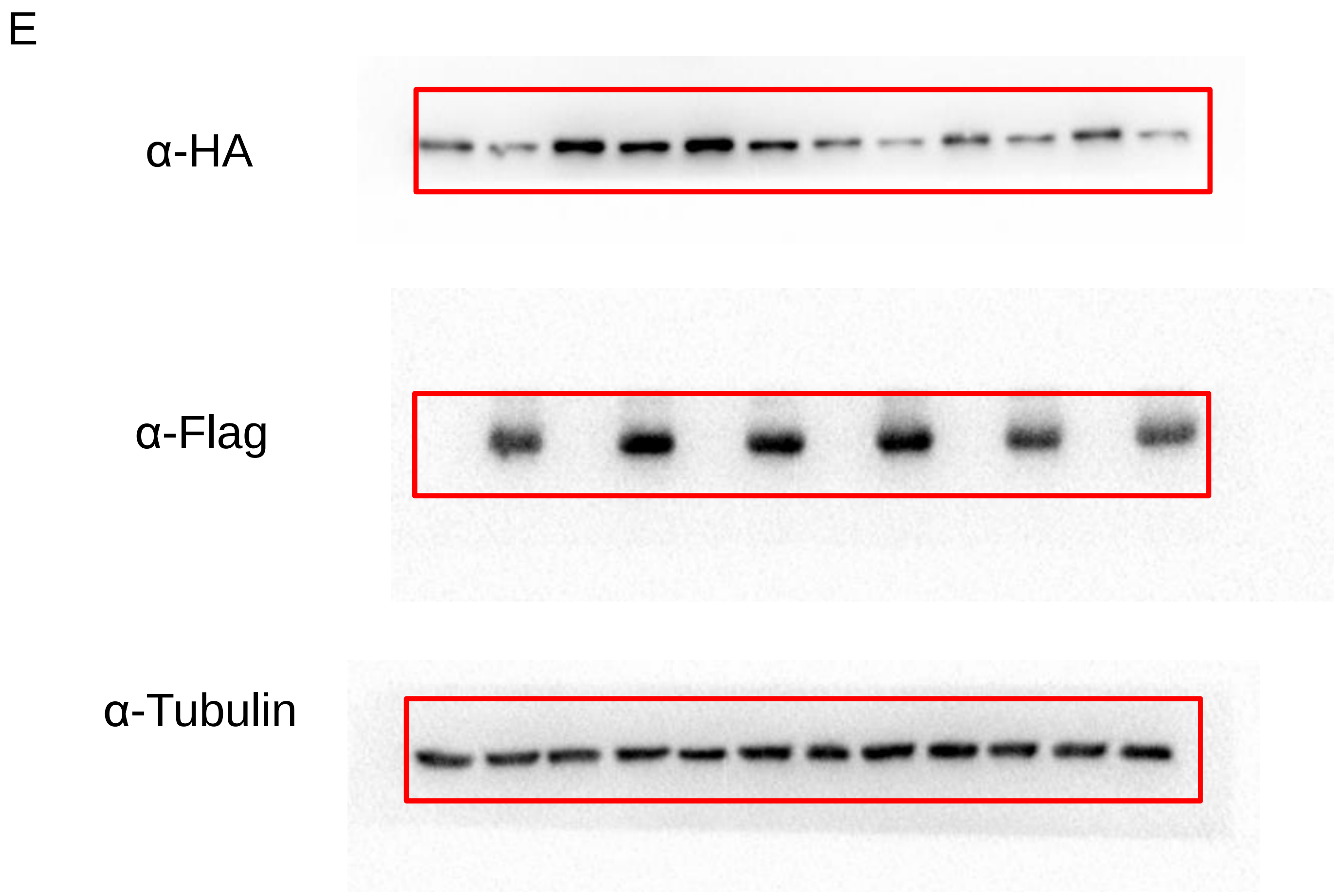

Figure 4-figure supplement 4

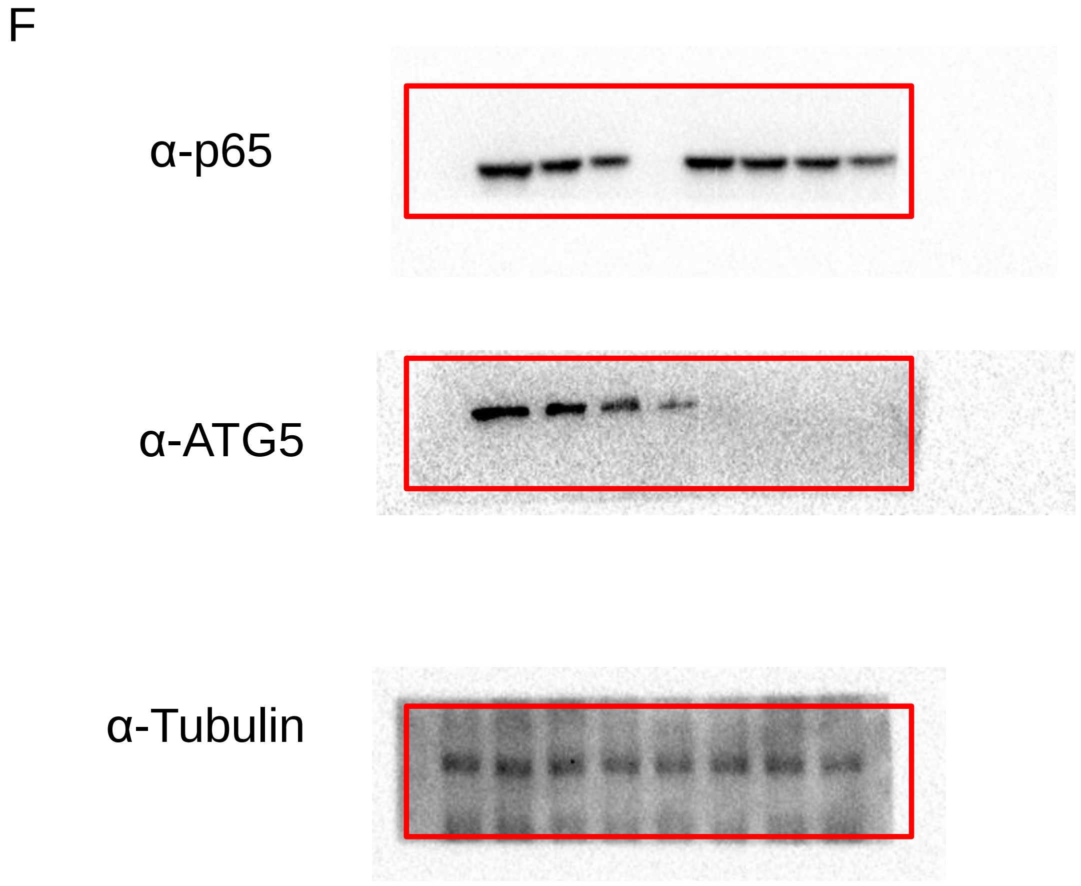

Figure 4-figure supplement 4

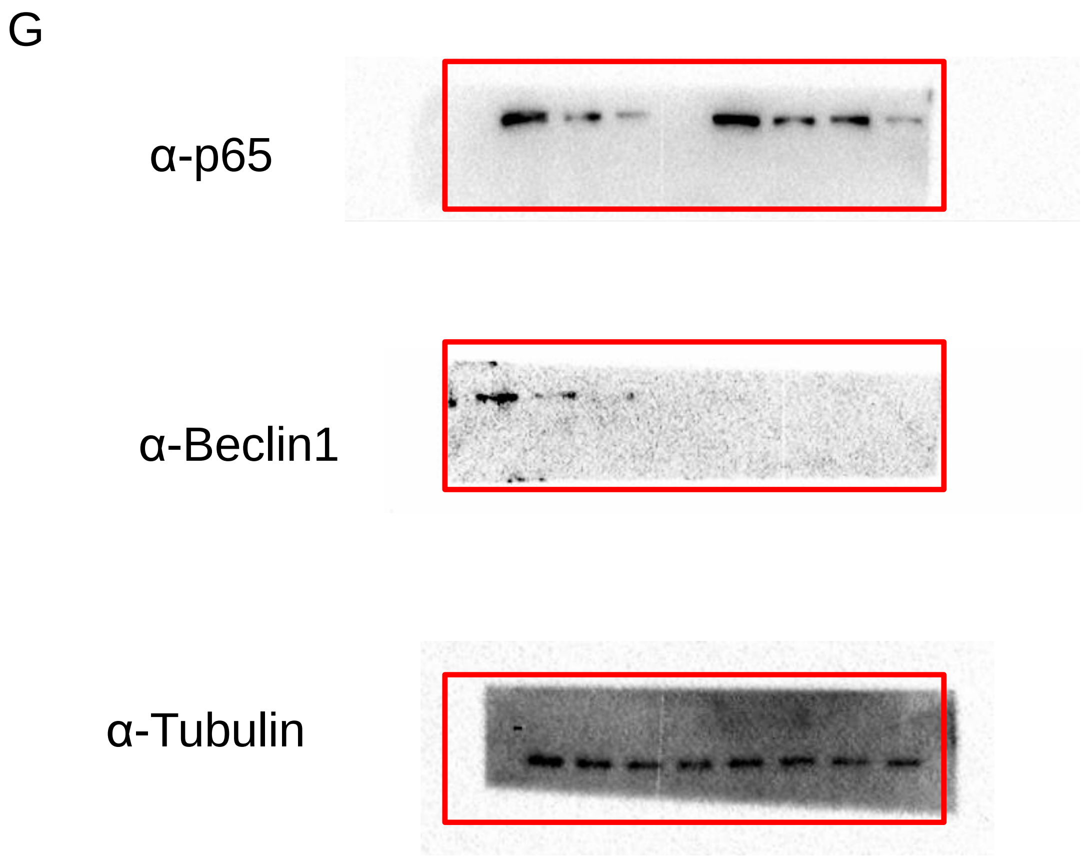

Supplement: Figure 4—source data 1. [file elife-87935-fig4-data1.pdf]
