# Supplementary figures and images for: Vangl2 suppresses NF-κB signaling and ameliorates sepsis by targeting p65 for NDP52-mediated autophagic degradation (part 2 of 2)
Source: eLife. 2024 Sep 13;12:RP87935. doi: 10.7554/eLife.87935 (PMC11398866; doi:10.7554/eLife.87935)

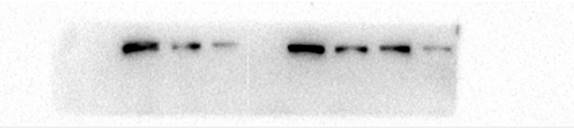

Supplement: Figure 4—source data 3. [file elife-87935-fig4-data3.zip › Figure 4-Source data 8/FigS4-G/FigS4G-p65.jpg]

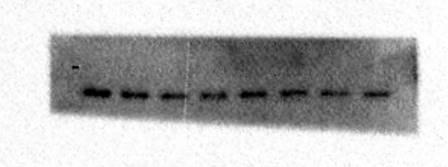

Supplement: Figure 4—source data 3. [file elife-87935-fig4-data3.zip › Figure 4-Source data 8/FigS4-G/FigS4G-Tubulin.jpg]

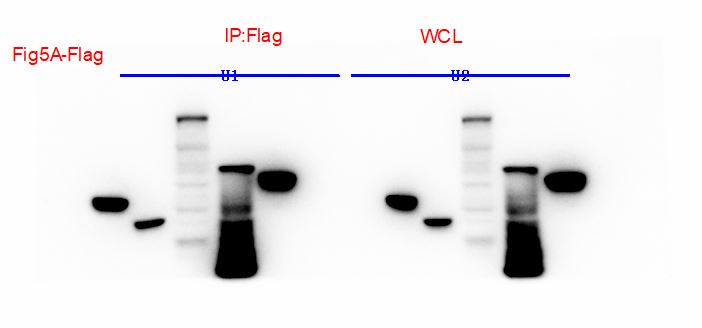

Supplement: Figure 5—source data 3. [file elife-87935-fig5-data3.zip › Figure 5-Source data 10/Fig5-A/Fig5A-Flag.JPG]

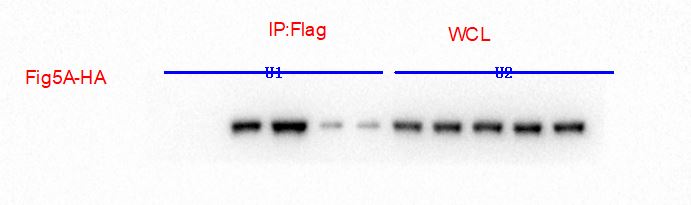

Supplement: Figure 5—source data 3. [file elife-87935-fig5-data3.zip › Figure 5-Source data 10/Fig5-A/Fig5A-HA.JPG]

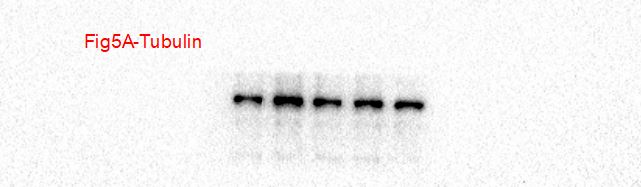

Supplement: Figure 5—source data 3. [file elife-87935-fig5-data3.zip › Figure 5-Source data 10/Fig5-A/Fig5A-Tubulin.JPG]

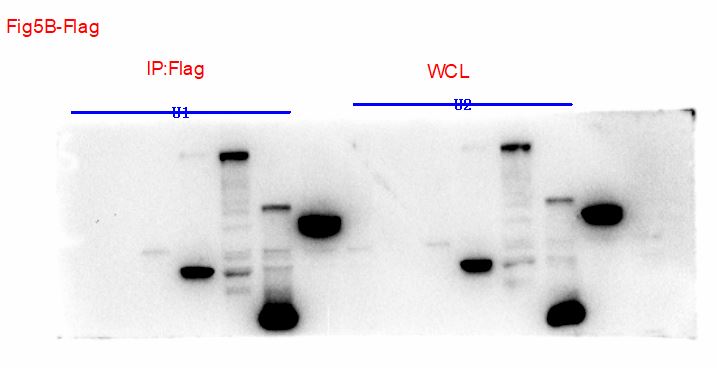

Supplement: Figure 5—source data 3. [file elife-87935-fig5-data3.zip › Figure 5-Source data 10/Fig5-B/Fig5B-Flag.JPG]

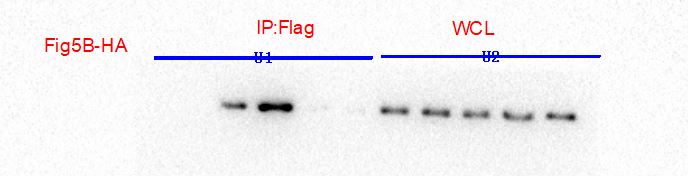

Supplement: Figure 5—source data 3. [file elife-87935-fig5-data3.zip › Figure 5-Source data 10/Fig5-B/Fig5B-HA.JPG]

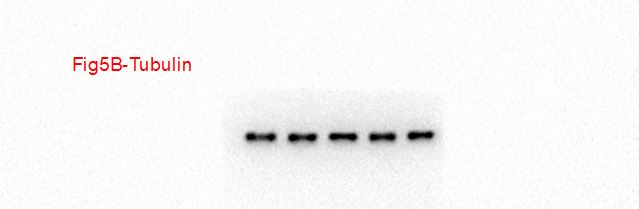

Supplement: Figure 5—source data 3. [file elife-87935-fig5-data3.zip › Figure 5-Source data 10/Fig5-B/Fig5B-Tubulin.JPG]

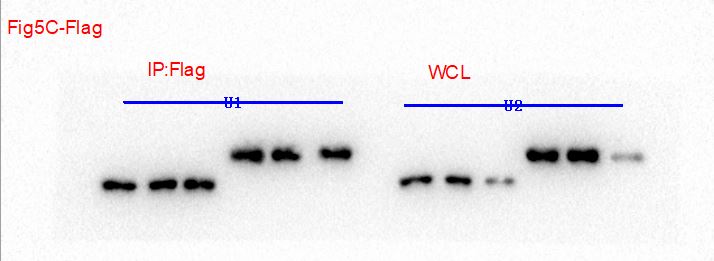

Supplement: Figure 5—source data 3. [file elife-87935-fig5-data3.zip › Figure 5-Source data 10/Fig5-C/Fig5C-Flag.JPG]

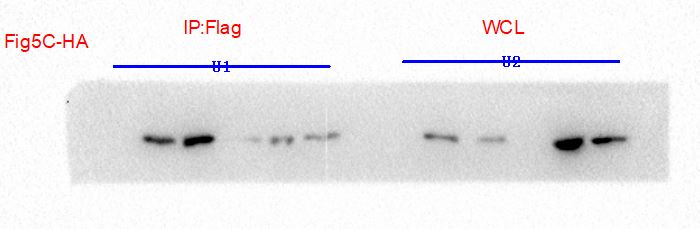

Supplement: Figure 5—source data 3. [file elife-87935-fig5-data3.zip › Figure 5-Source data 10/Fig5-C/Fig5C-HA.JPG]

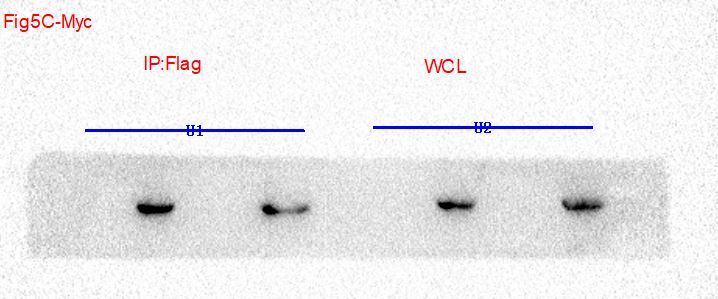

Supplement: Figure 5—source data 3. [file elife-87935-fig5-data3.zip › Figure 5-Source data 10/Fig5-C/Fig5C-Myc.JPG]

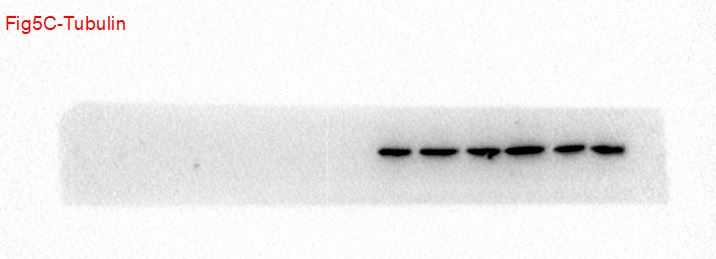

Supplement: Figure 5—source data 3. [file elife-87935-fig5-data3.zip › Figure 5-Source data 10/Fig5-C/Fig5C-Tubulin.JPG]

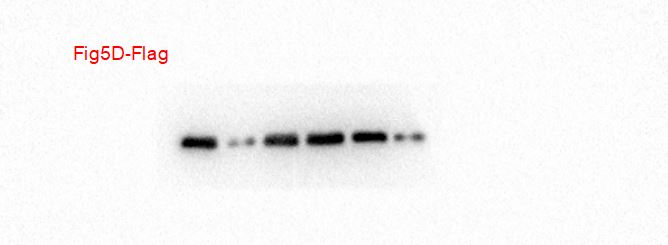

Supplement: Figure 5—source data 3. [file elife-87935-fig5-data3.zip › Figure 5-Source data 10/Fig5-D/Fig5D-Flag.JPG]

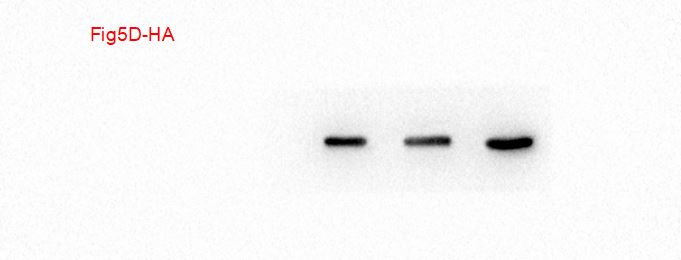

Supplement: Figure 5—source data 3. [file elife-87935-fig5-data3.zip › Figure 5-Source data 10/Fig5-D/Fig5D-HA.JPG]

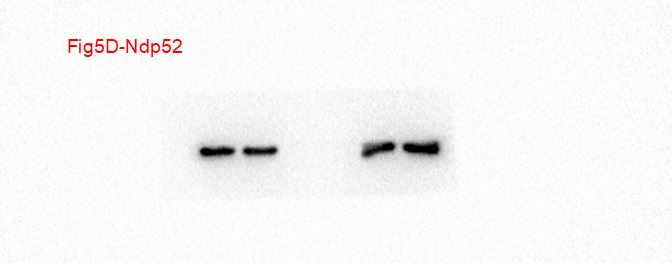

Supplement: Figure 5—source data 3. [file elife-87935-fig5-data3.zip › Figure 5-Source data 10/Fig5-D/Fig5D-NDP52.JPG]

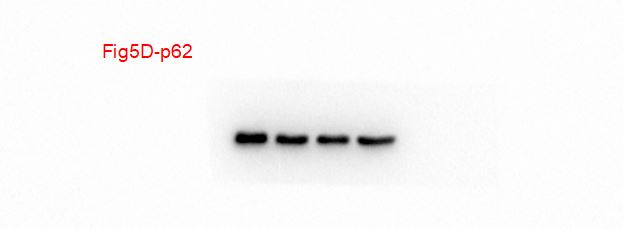

Supplement: Figure 5—source data 3. [file elife-87935-fig5-data3.zip › Figure 5-Source data 10/Fig5-D/Fig5D-p62.JPG]

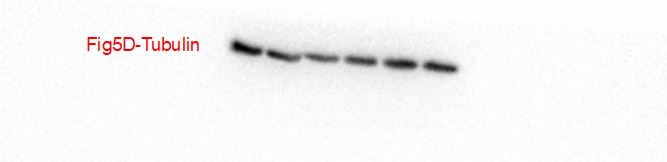

Supplement: Figure 5—source data 3. [file elife-87935-fig5-data3.zip › Figure 5-Source data 10/Fig5-D/Fig5D-Tubulin.JPG]

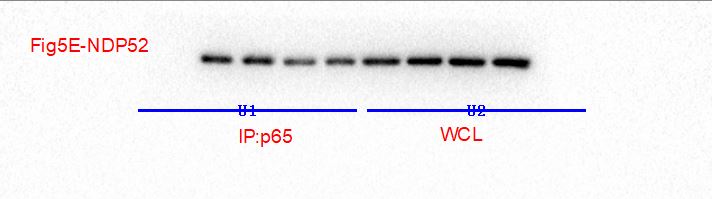

Supplement: Figure 5—source data 3. [file elife-87935-fig5-data3.zip › Figure 5-Source data 10/Fig5-E/Fig5E-NDP52.JPG]

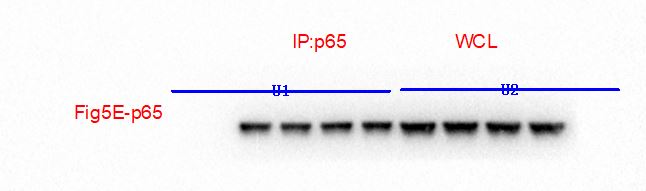

Supplement: Figure 5—source data 3. [file elife-87935-fig5-data3.zip › Figure 5-Source data 10/Fig5-E/Fig5E-p65.JPG]

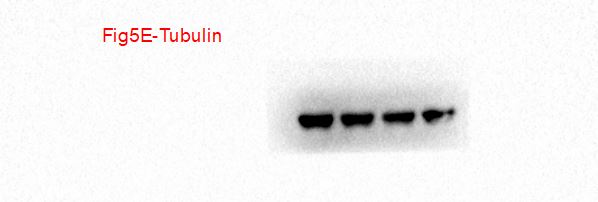

Supplement: Figure 5—source data 3. [file elife-87935-fig5-data3.zip › Figure 5-Source data 10/Fig5-E/Fig5E-Tubulin.JPG]

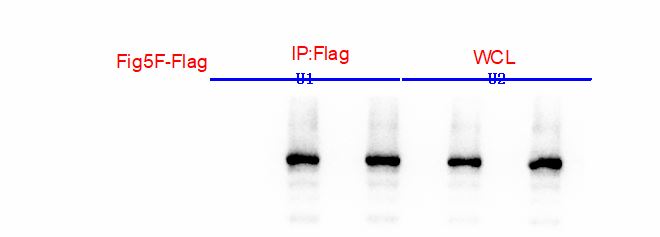

Supplement: Figure 5—source data 3. [file elife-87935-fig5-data3.zip › Figure 5-Source data 10/Fig5-F/Fig5F-Flag.JPG]

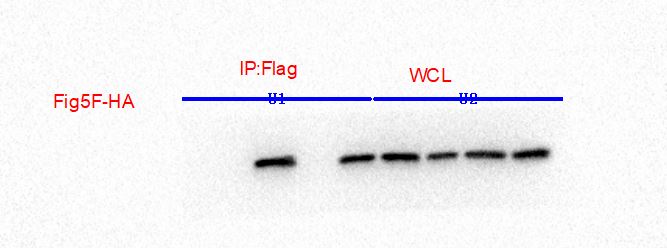

Supplement: Figure 5—source data 3. [file elife-87935-fig5-data3.zip › Figure 5-Source data 10/Fig5-F/Fig5F-HA.JPG]

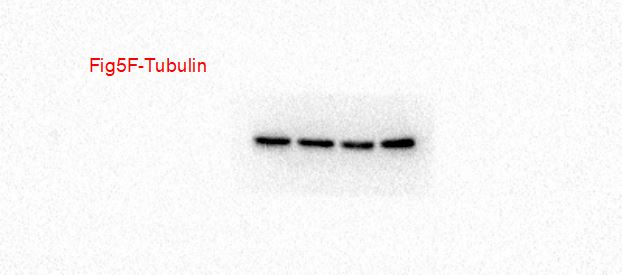

Supplement: Figure 5—source data 3. [file elife-87935-fig5-data3.zip › Figure 5-Source data 10/Fig5-F/Fig5F-Tubulin.JPG]

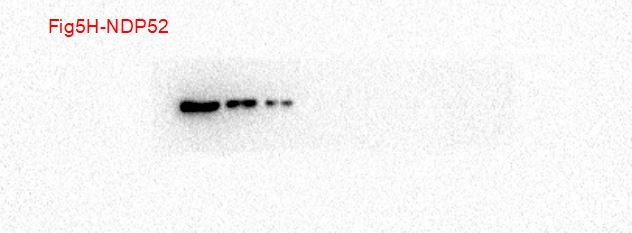

Supplement: Figure 5—source data 3. [file elife-87935-fig5-data3.zip › Figure 5-Source data 10/Fig5-H/Fig5H-NDP52.JPG]

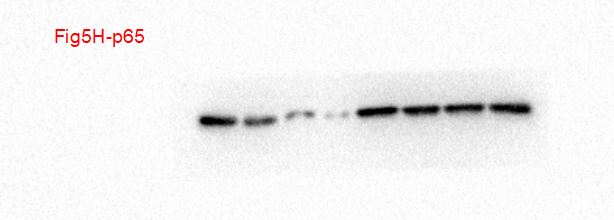

Supplement: Figure 5—source data 3. [file elife-87935-fig5-data3.zip › Figure 5-Source data 10/Fig5-H/Fig5H-p65.JPG]

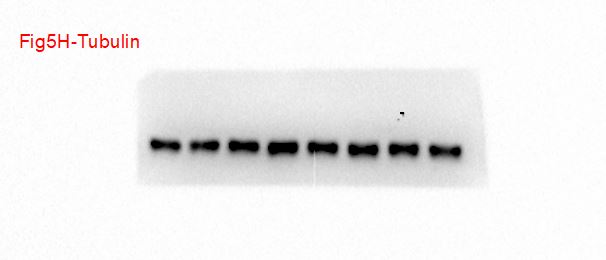

Supplement: Figure 5—source data 3. [file elife-87935-fig5-data3.zip › Figure 5-Source data 10/Fig5-H/Fig5H-Tubulin.JPG]

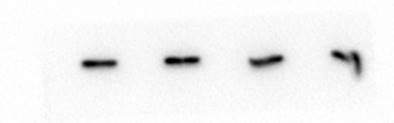

Supplement: Figure 5—source data 3. [file elife-87935-fig5-data3.zip › Figure 5-Source data 10/FigS5-A/FigS5A-Flag-ip-WCL.jpg]

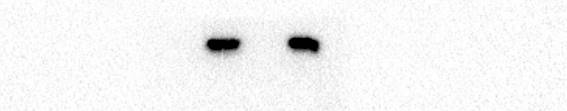

Supplement: Figure 5—source data 3. [file elife-87935-fig5-data3.zip › Figure 5-Source data 10/FigS5-A/FigS5A-IP-HA.jpg]

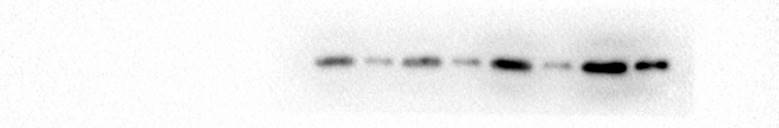

Supplement: Figure 5—source data 3. [file elife-87935-fig5-data3.zip › Figure 5-Source data 10/FigS5-A/FigS5A-WCL-HA.jpg]

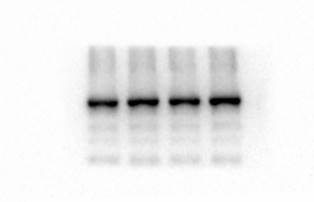

Supplement: Figure 5—source data 3. [file elife-87935-fig5-data3.zip › Figure 5-Source data 10/FigS5-A/FigS5A-WCL-Tubulin.jpg]

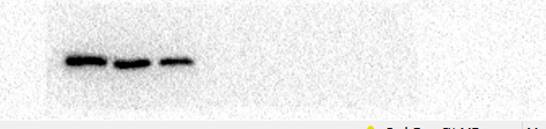

Supplement: Figure 5—source data 3. [file elife-87935-fig5-data3.zip › Figure 5-Source data 10/FigS5-C/FigS5C-p62.jpg]

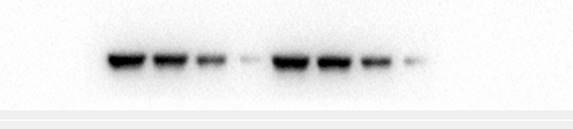

Supplement: Figure 5—source data 3. [file elife-87935-fig5-data3.zip › Figure 5-Source data 10/FigS5-C/FigS5C-p65.jpg]

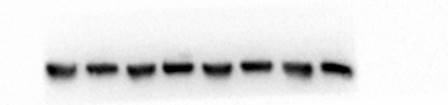

Supplement: Figure 5—source data 3. [file elife-87935-fig5-data3.zip › Figure 5-Source data 10/FigS5-C/FigS5C-tubulin.jpg]

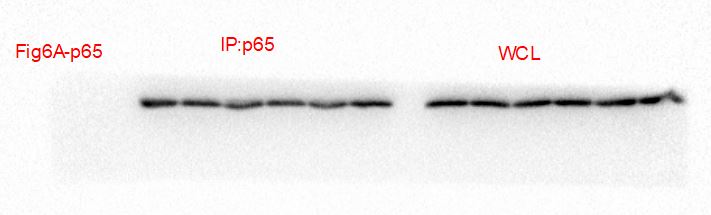

Supplement: Figure 6—source data 2. [file elife-87935-fig6-data2.zip › Figure 6-Source data 12/Fig6-A/Fig6A-p65.JPG]

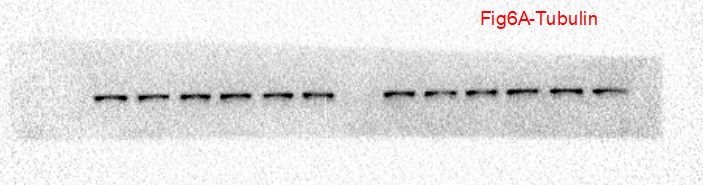

Supplement: Figure 6—source data 2. [file elife-87935-fig6-data2.zip › Figure 6-Source data 12/Fig6-A/Fig6A-Tubulin.JPG]

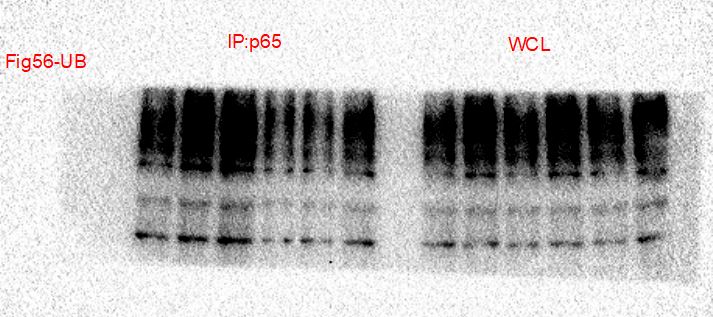

Supplement: Figure 6—source data 2. [file elife-87935-fig6-data2.zip › Figure 6-Source data 12/Fig6-A/Fig6A-Ub.JPG]

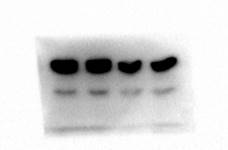

Supplement: Figure 6—source data 2. [file elife-87935-fig6-data2.zip › Figure 6-Source data 12/Fig6-B/Fig6B-IP-Flag.jpg]

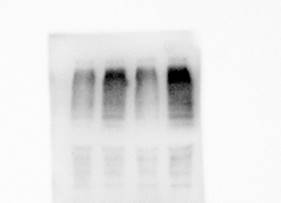

Supplement: Figure 6—source data 2. [file elife-87935-fig6-data2.zip › Figure 6-Source data 12/Fig6-B/Fig6B-IP-HA.jpg]

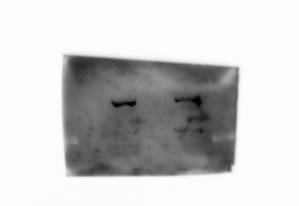

Supplement: Figure 6—source data 2. [file elife-87935-fig6-data2.zip › Figure 6-Source data 12/Fig6-B/Fig6B-IP-Myc.jpg]

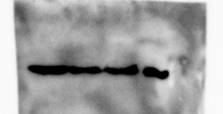

Supplement: Figure 6—source data 2. [file elife-87935-fig6-data2.zip › Figure 6-Source data 12/Fig6-B/Fig6B-WCL-Flag.jpg]

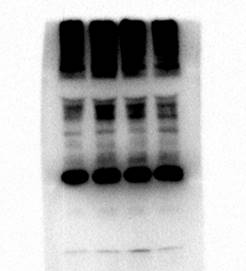

Supplement: Figure 6—source data 2. [file elife-87935-fig6-data2.zip › Figure 6-Source data 12/Fig6-B/Fig6B-WCL-HA.jpg]

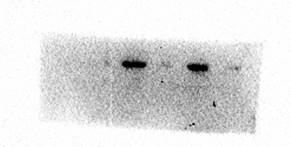

Supplement: Figure 6—source data 2. [file elife-87935-fig6-data2.zip › Figure 6-Source data 12/Fig6-B/Fig6B-WCL-Myc.jpg]

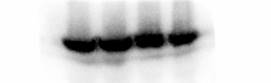

Supplement: Figure 6—source data 2. [file elife-87935-fig6-data2.zip › Figure 6-Source data 12/Fig6-B/Fig6B-WCL-Tubulin.jpg]

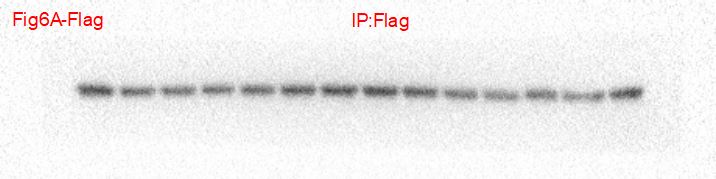

Supplement: Figure 6—source data 2. [file elife-87935-fig6-data2.zip › Figure 6-Source data 12/Fig6-C/Fig6C-Flag-IP.JPG]

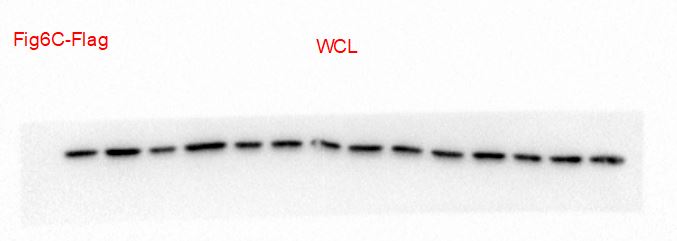

Supplement: Figure 6—source data 2. [file elife-87935-fig6-data2.zip › Figure 6-Source data 12/Fig6-C/Fig6C-Flag-WCL.JPG]

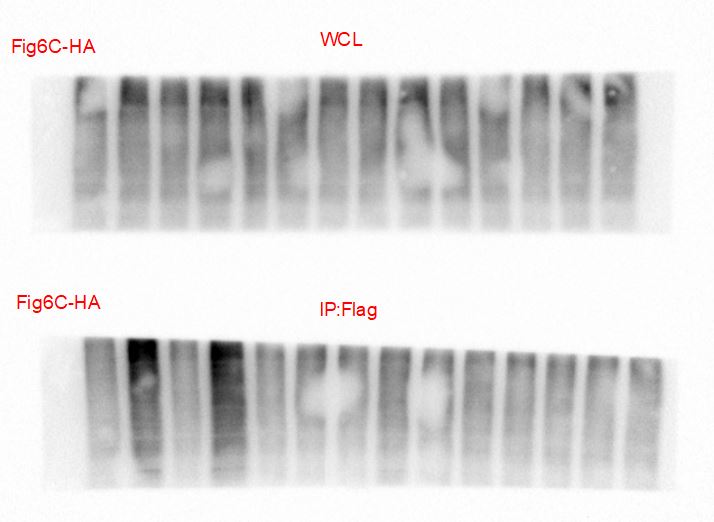

Supplement: Figure 6—source data 2. [file elife-87935-fig6-data2.zip › Figure 6-Source data 12/Fig6-C/Fig6C-HA.JPG]

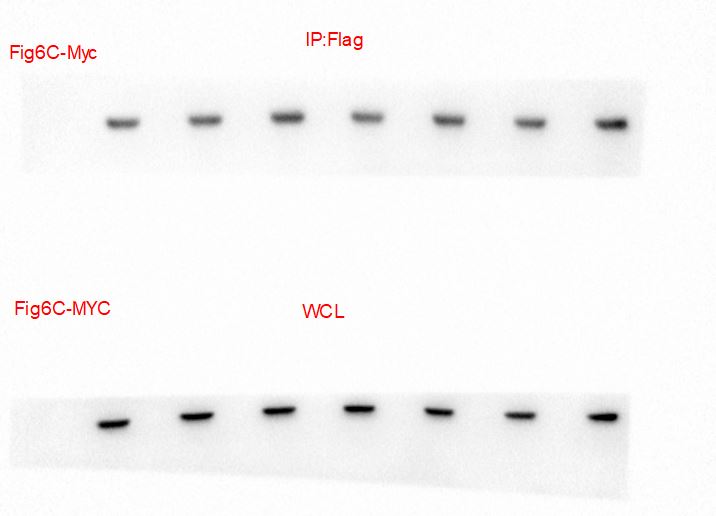

Supplement: Figure 6—source data 2. [file elife-87935-fig6-data2.zip › Figure 6-Source data 12/Fig6-C/Fig6C-MYC.JPG]

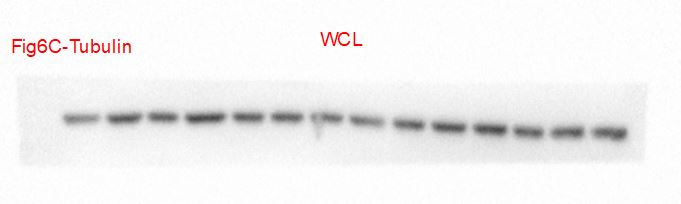

Supplement: Figure 6—source data 2. [file elife-87935-fig6-data2.zip › Figure 6-Source data 12/Fig6-C/Fig6C-Tubulin.JPG]

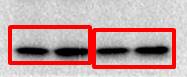

Supplement: Figure 6—source data 2. [file elife-87935-fig6-data2.zip › Figure 6-Source data 12/Fig6-D/Fig6B-IP-WCL-Flag.jpg]

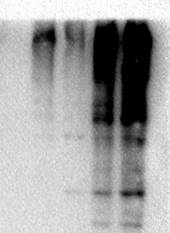

Supplement: Figure 6—source data 2. [file elife-87935-fig6-data2.zip › Figure 6-Source data 12/Fig6-D/Fig6B-IP-WCL-HA.jpg]

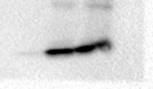

Supplement: Figure 6—source data 2. [file elife-87935-fig6-data2.zip › Figure 6-Source data 12/Fig6-D/Fig6B-WCL-Tubulin.jpg]

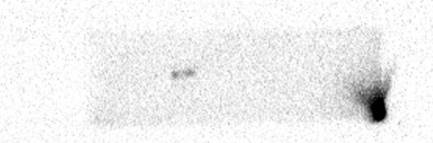

Supplement: Figure 6—source data 2. [file elife-87935-fig6-data2.zip › Figure 6-Source data 12/Fig6-D/Fig6B-WCL-Vangl2.jpg]

Figure 7

A

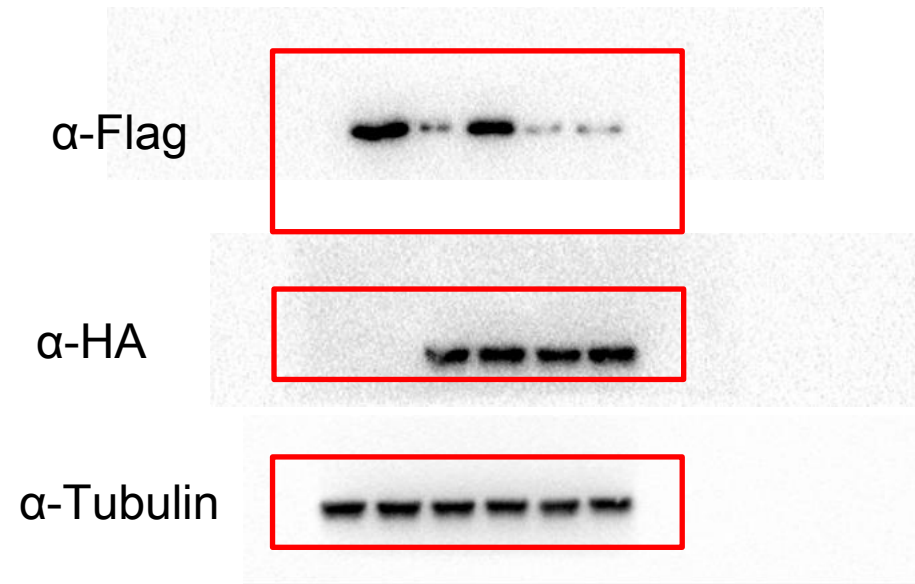

Figure 7

D

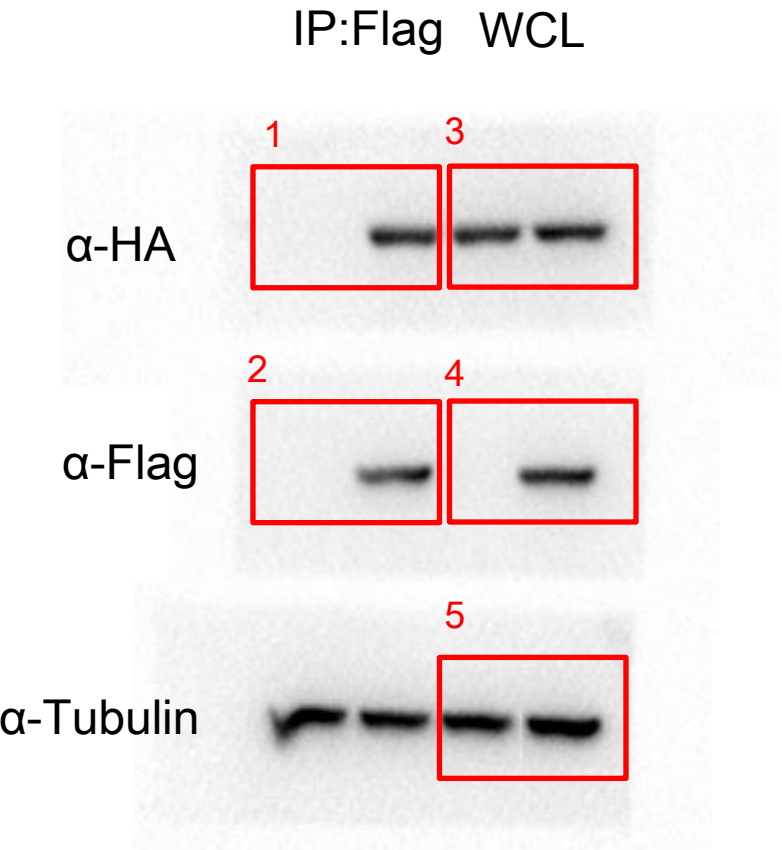

Figure 7

E

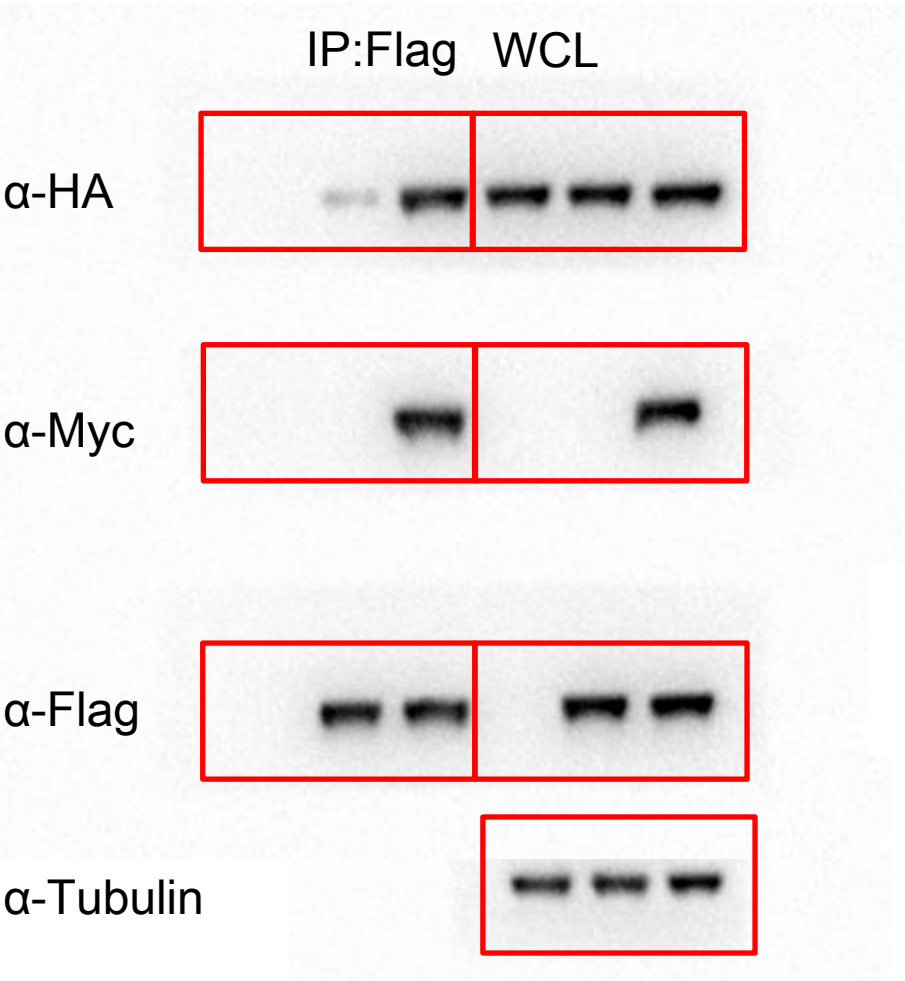

Figure 7

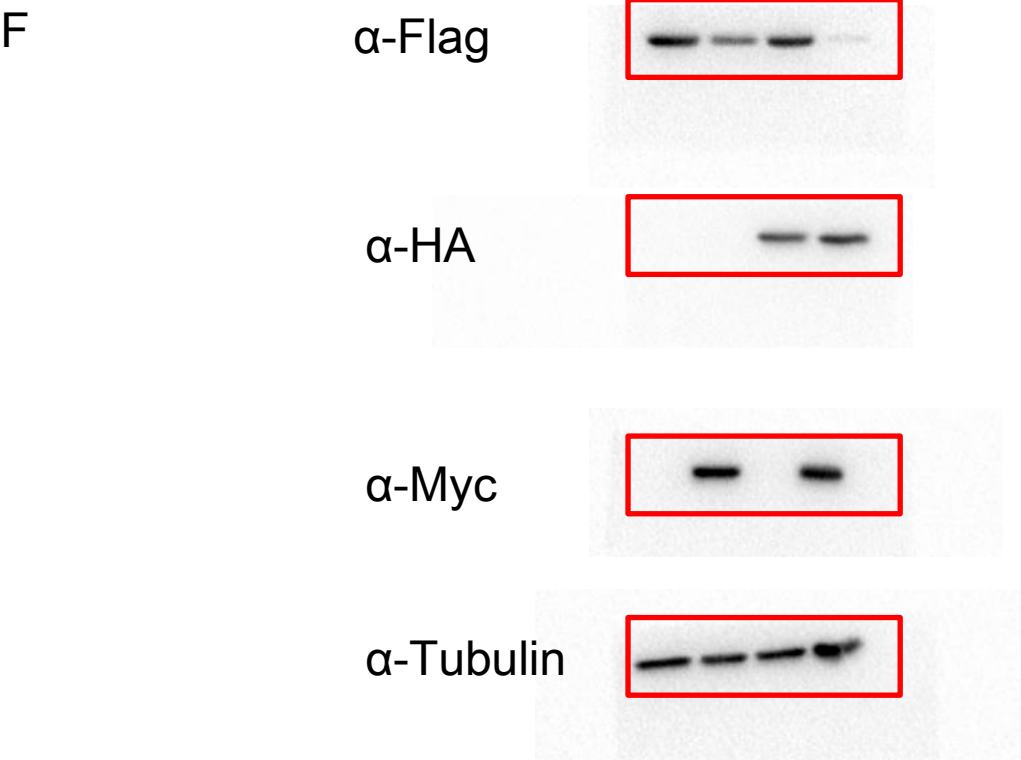

Figure 7

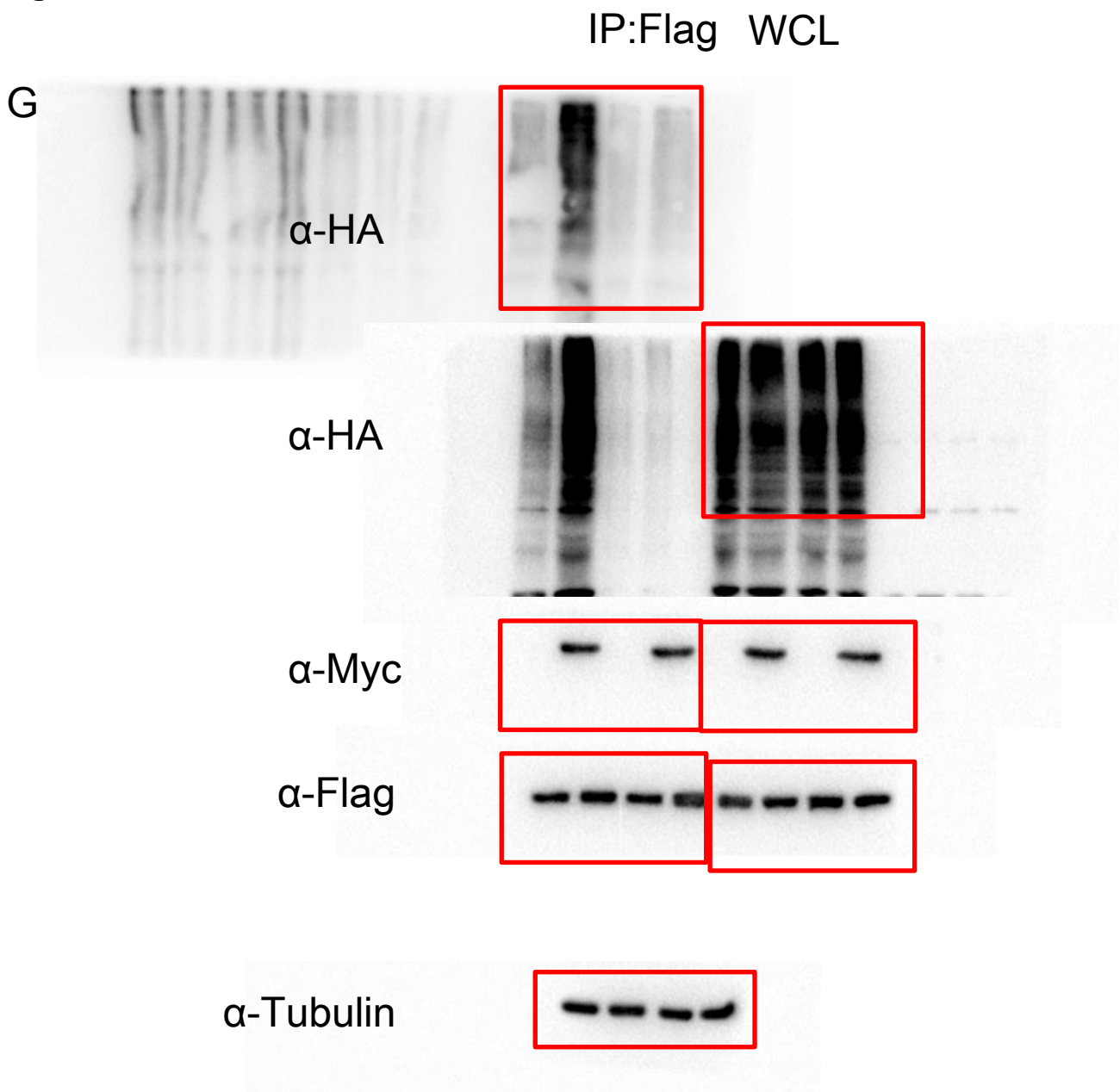

Figure 7

H

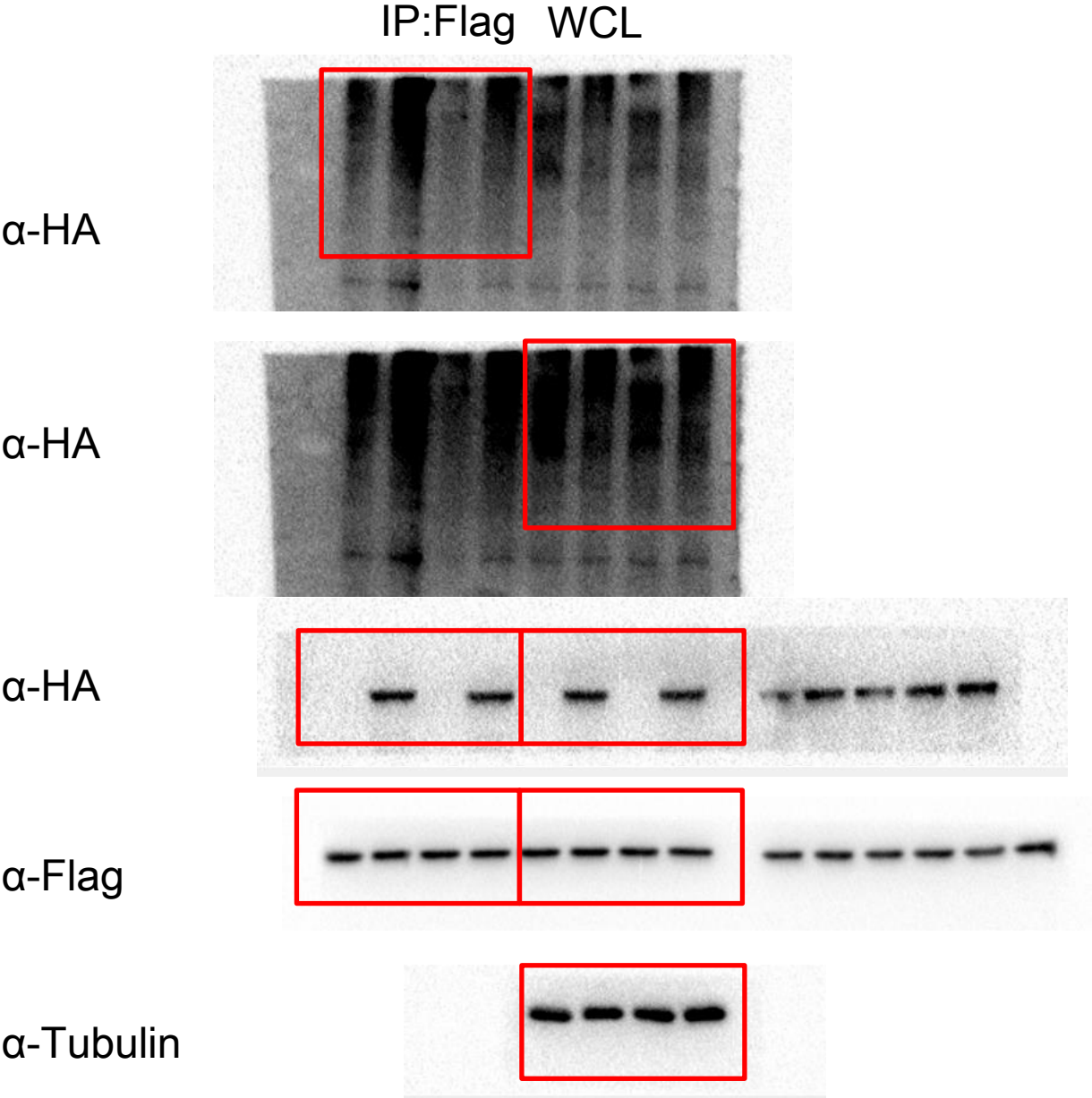

Supplement: Figure 7—source data 1. [file elife-87935-fig7-data1.pdf]

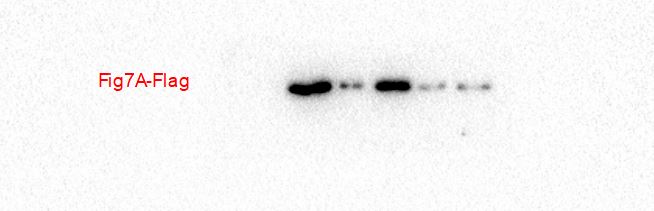

Supplement: Figure 7—source data 2. [file elife-87935-fig7-data2.zip › Figure 7-Source data 14/Fig7-A/Fig7A-Flag.JPG]

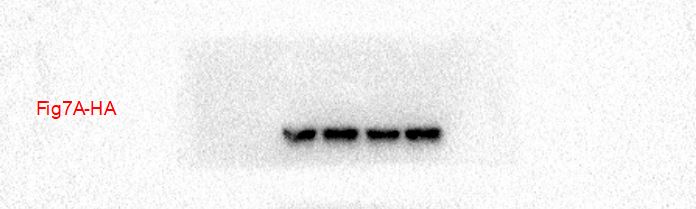

Supplement: Figure 7—source data 2. [file elife-87935-fig7-data2.zip › Figure 7-Source data 14/Fig7-A/Fig7A-HA.JPG]

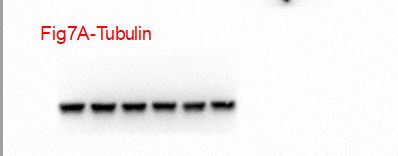

Supplement: Figure 7—source data 2. [file elife-87935-fig7-data2.zip › Figure 7-Source data 14/Fig7-A/Fig7A-Tubulin.JPG]

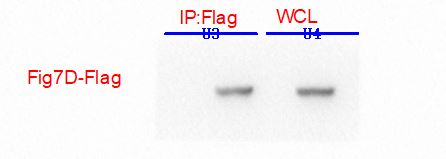

Supplement: Figure 7—source data 2. [file elife-87935-fig7-data2.zip › Figure 7-Source data 14/Fig7-D/Fig7D-Flag.JPG]

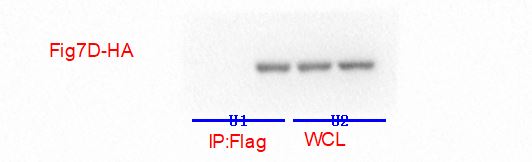

Supplement: Figure 7—source data 2. [file elife-87935-fig7-data2.zip › Figure 7-Source data 14/Fig7-D/Fig7D-HA.JPG]

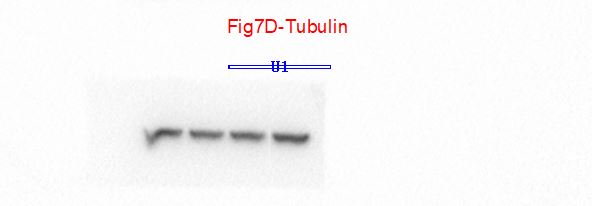

Supplement: Figure 7—source data 2. [file elife-87935-fig7-data2.zip › Figure 7-Source data 14/Fig7-D/Fig7D-Tubulin.JPG]

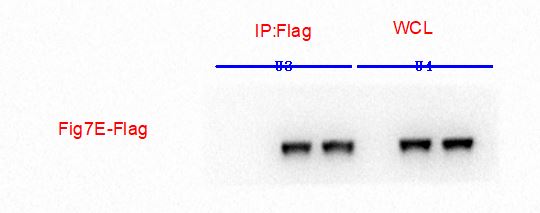

Supplement: Figure 7—source data 2. [file elife-87935-fig7-data2.zip › Figure 7-Source data 14/Fig7-E/Fig7E-Flag.JPG]

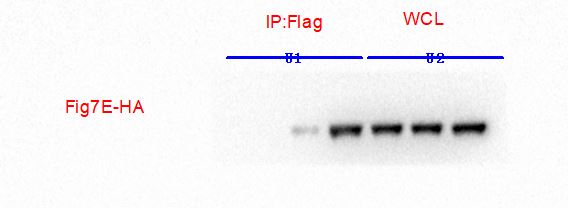

Supplement: Figure 7—source data 2. [file elife-87935-fig7-data2.zip › Figure 7-Source data 14/Fig7-E/Fig7E-HA.JPG]

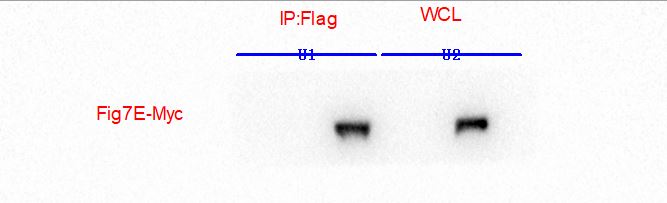

Supplement: Figure 7—source data 2. [file elife-87935-fig7-data2.zip › Figure 7-Source data 14/Fig7-E/Fig7E-MYC.JPG]

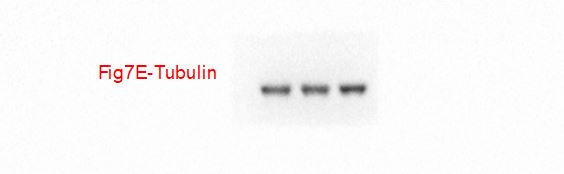

Supplement: Figure 7—source data 2. [file elife-87935-fig7-data2.zip › Figure 7-Source data 14/Fig7-E/Fig7E-Tubulin.JPG]

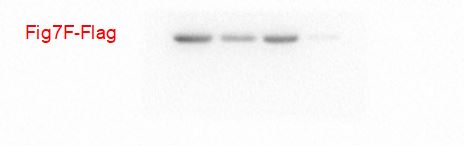

Supplement: Figure 7—source data 2. [file elife-87935-fig7-data2.zip › Figure 7-Source data 14/Fig7-F/Fig7F-Flag.JPG]

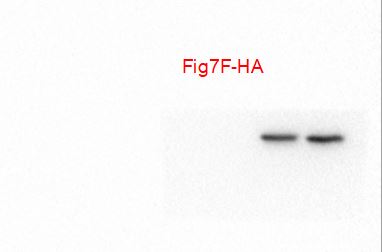

Supplement: Figure 7—source data 2. [file elife-87935-fig7-data2.zip › Figure 7-Source data 14/Fig7-F/Fig7F-HA.JPG]

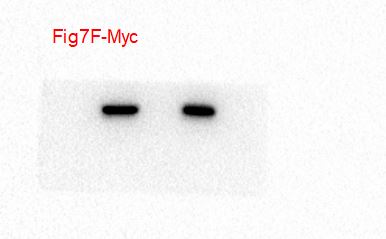

Supplement: Figure 7—source data 2. [file elife-87935-fig7-data2.zip › Figure 7-Source data 14/Fig7-F/Fig7F-myc.JPG]

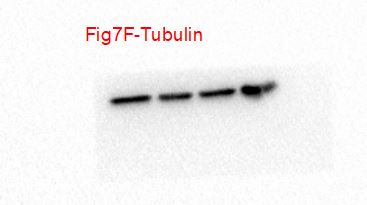

Supplement: Figure 7—source data 2. [file elife-87935-fig7-data2.zip › Figure 7-Source data 14/Fig7-F/Fig7F-Tubulin.JPG]

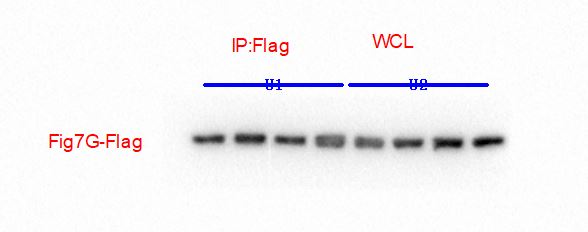

Supplement: Figure 7—source data 2. [file elife-87935-fig7-data2.zip › Figure 7-Source data 14/Fig7-G/Fig7G-Flag.JPG]

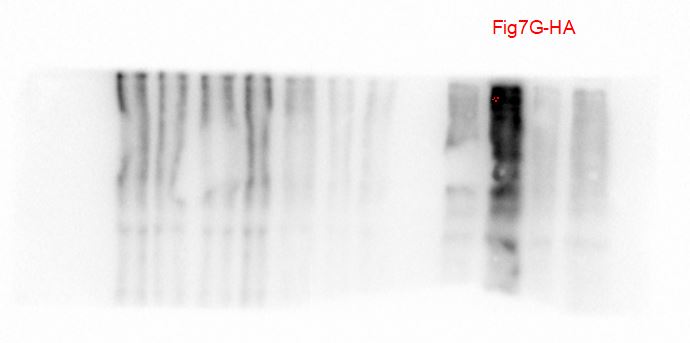

Supplement: Figure 7—source data 2. [file elife-87935-fig7-data2.zip › Figure 7-Source data 14/Fig7-G/Fig7G-HA-IP.JPG]

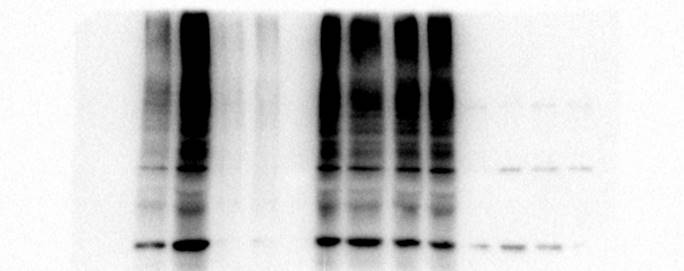

Supplement: Figure 7—source data 2. [file elife-87935-fig7-data2.zip › Figure 7-Source data 14/Fig7-G/Fig7G-HA-WCL.JPG]

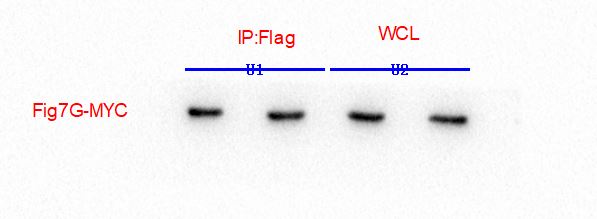

Supplement: Figure 7—source data 2. [file elife-87935-fig7-data2.zip › Figure 7-Source data 14/Fig7-G/Fig7G-MYC.JPG]

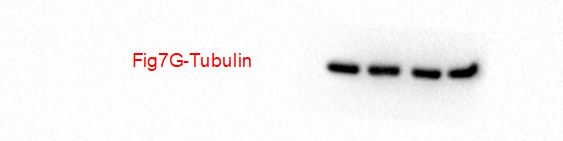

Supplement: Figure 7—source data 2. [file elife-87935-fig7-data2.zip › Figure 7-Source data 14/Fig7-G/Fig7G-Tubulin.JPG]

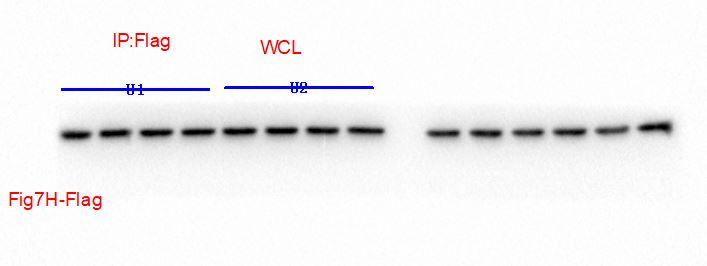

Supplement: Figure 7—source data 2. [file elife-87935-fig7-data2.zip › Figure 7-Source data 14/Fig7-H/Fig7H-Flag.JPG]

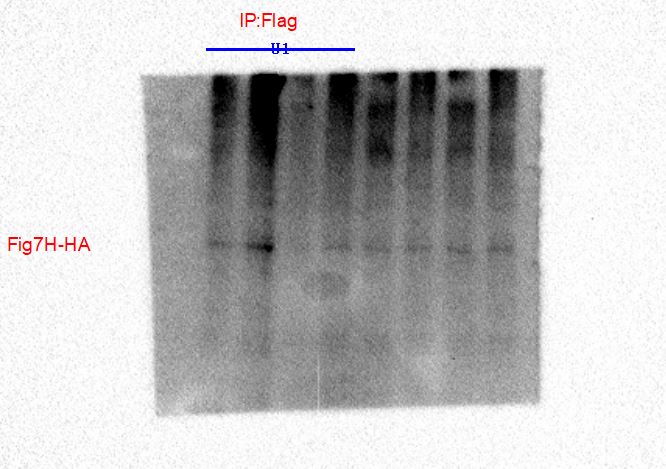

Supplement: Figure 7—source data 2. [file elife-87935-fig7-data2.zip › Figure 7-Source data 14/Fig7-H/Fig7H-HA-ip.JPG]

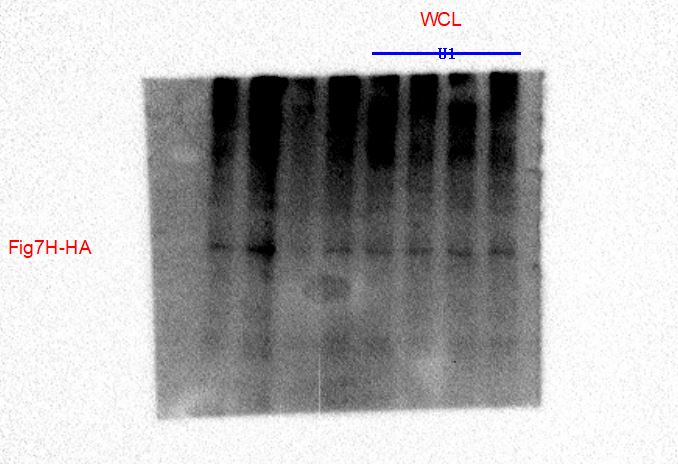

Supplement: Figure 7—source data 2. [file elife-87935-fig7-data2.zip › Figure 7-Source data 14/Fig7-H/Fig7H-HA-WCL.JPG]

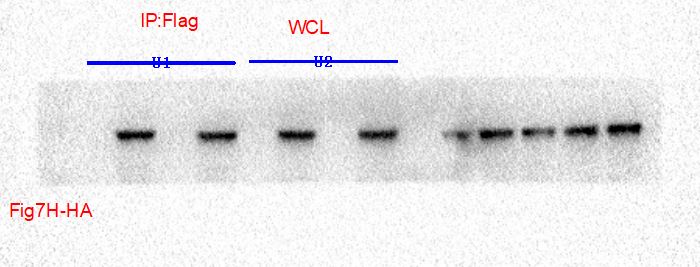

Supplement: Figure 7—source data 2. [file elife-87935-fig7-data2.zip › Figure 7-Source data 14/Fig7-H/Fig7H-HA.JPG]

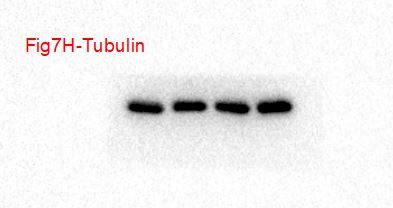

Supplement: Figure 7—source data 2. [file elife-87935-fig7-data2.zip › Figure 7-Source data 14/Fig7-H/Fig7H-tubulin.JPG]
