# Supplementary material for: Vangl2 suppresses NF-κB signaling and ameliorates sepsis by targeting p65 for NDP52-mediated autophagic degradation
Source: eLife. 2024 Sep 13;12:RP87935. doi: 10.7554/eLife.87935 (PMC11398866; doi:10.7554/eLife.87935)

Figure 5

A

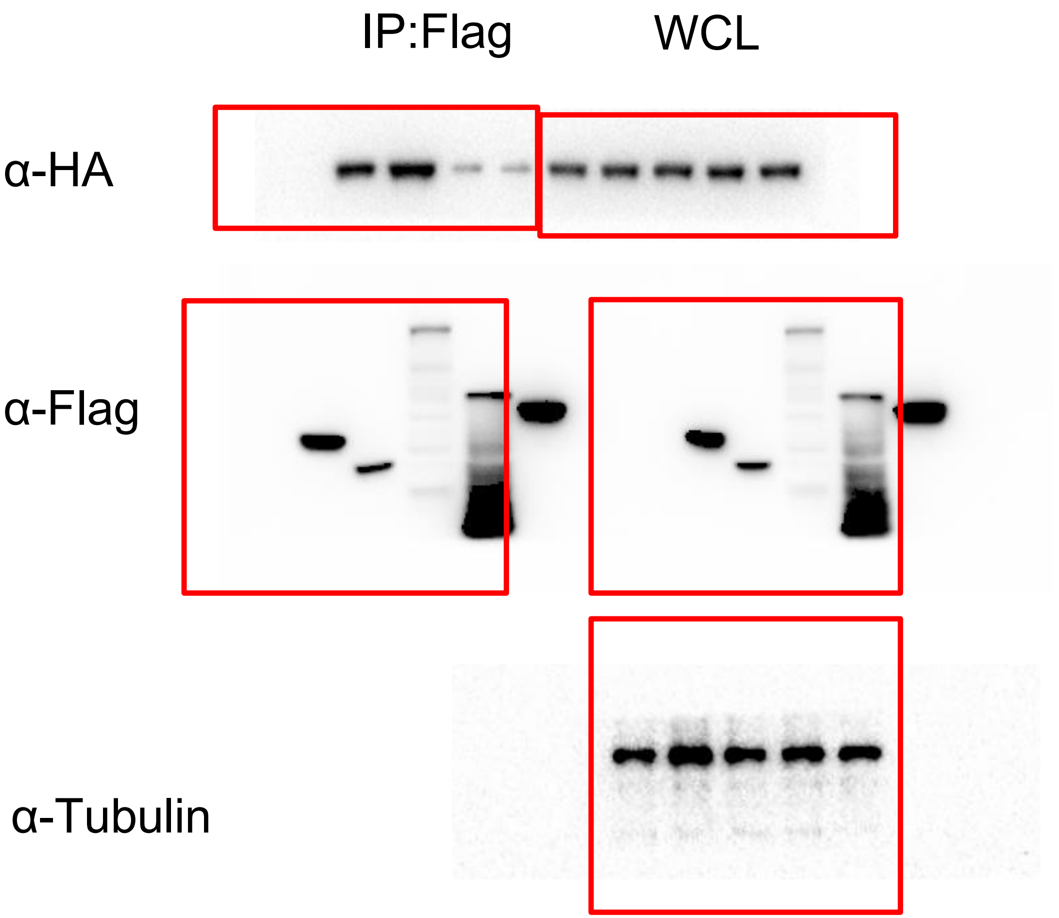

Figure 5

B

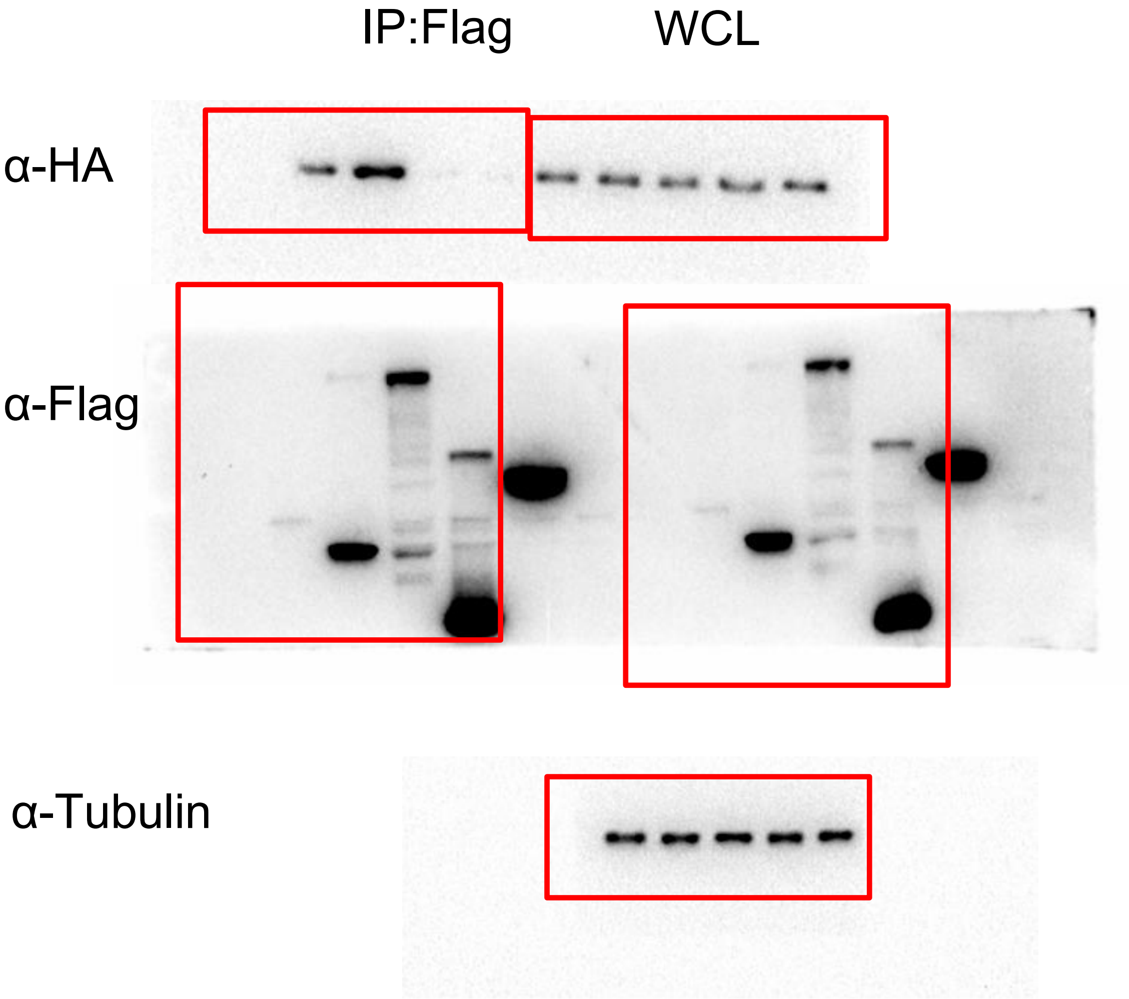

Figure 5

C

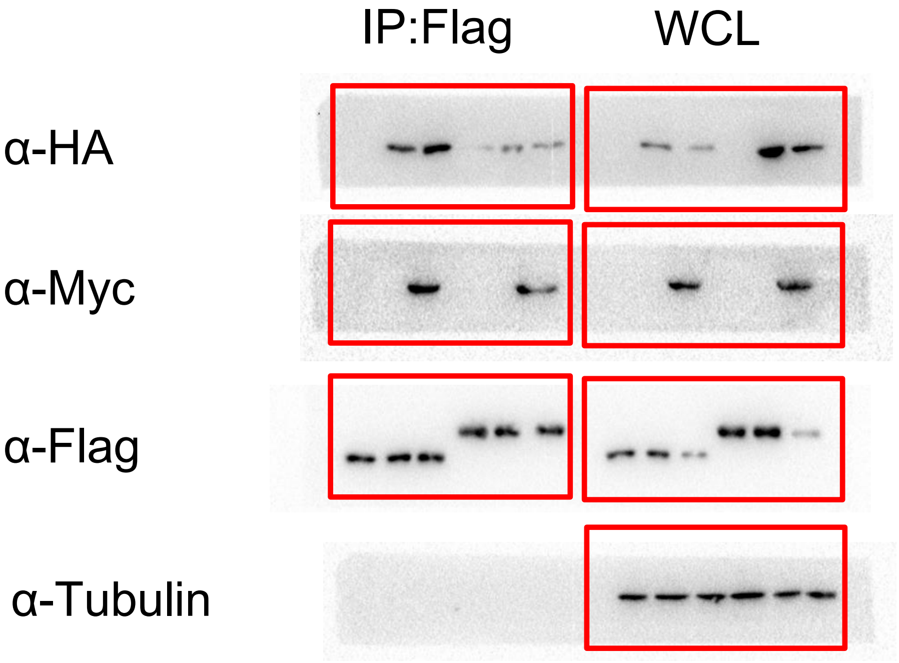

Figure 5

D

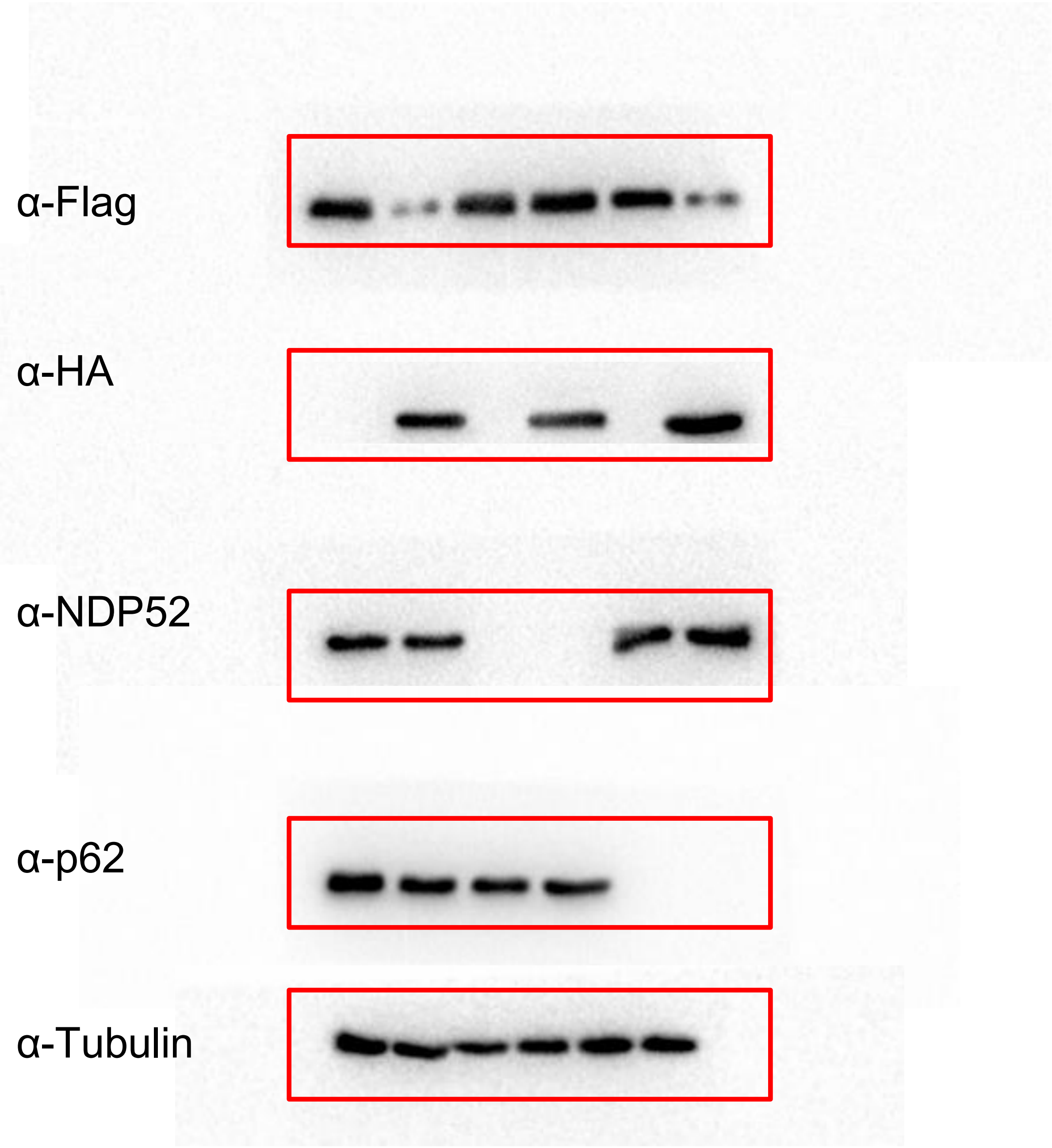

Figure 5

E

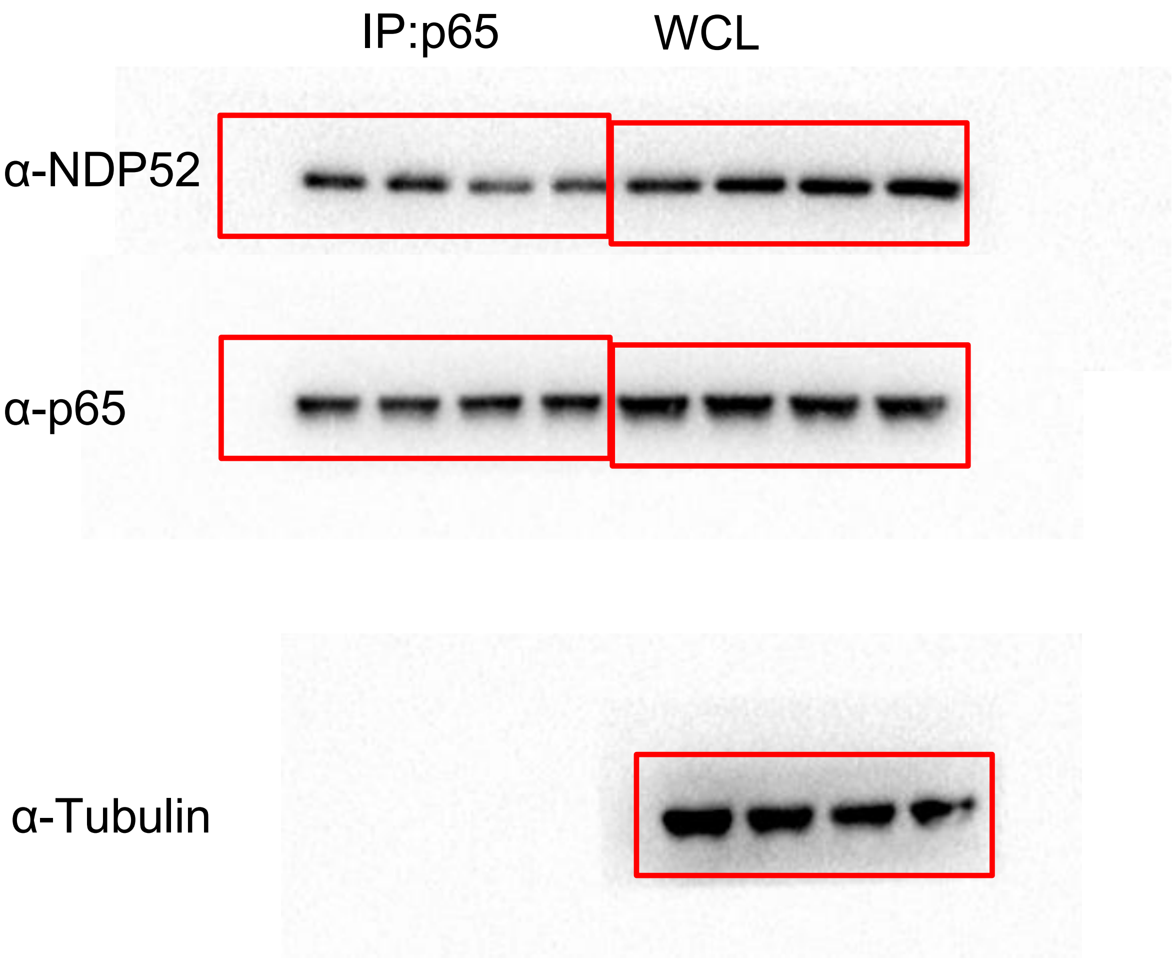

Figure 5

F

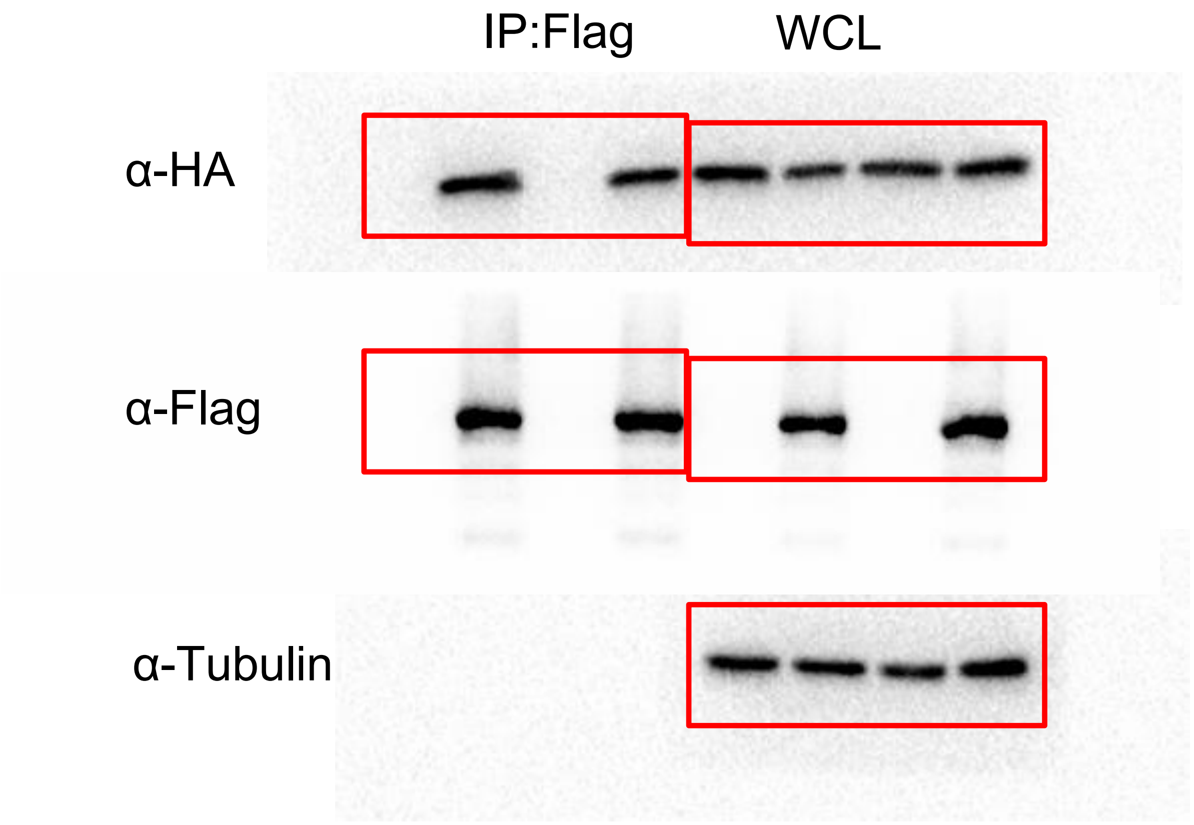

Figure 5

H

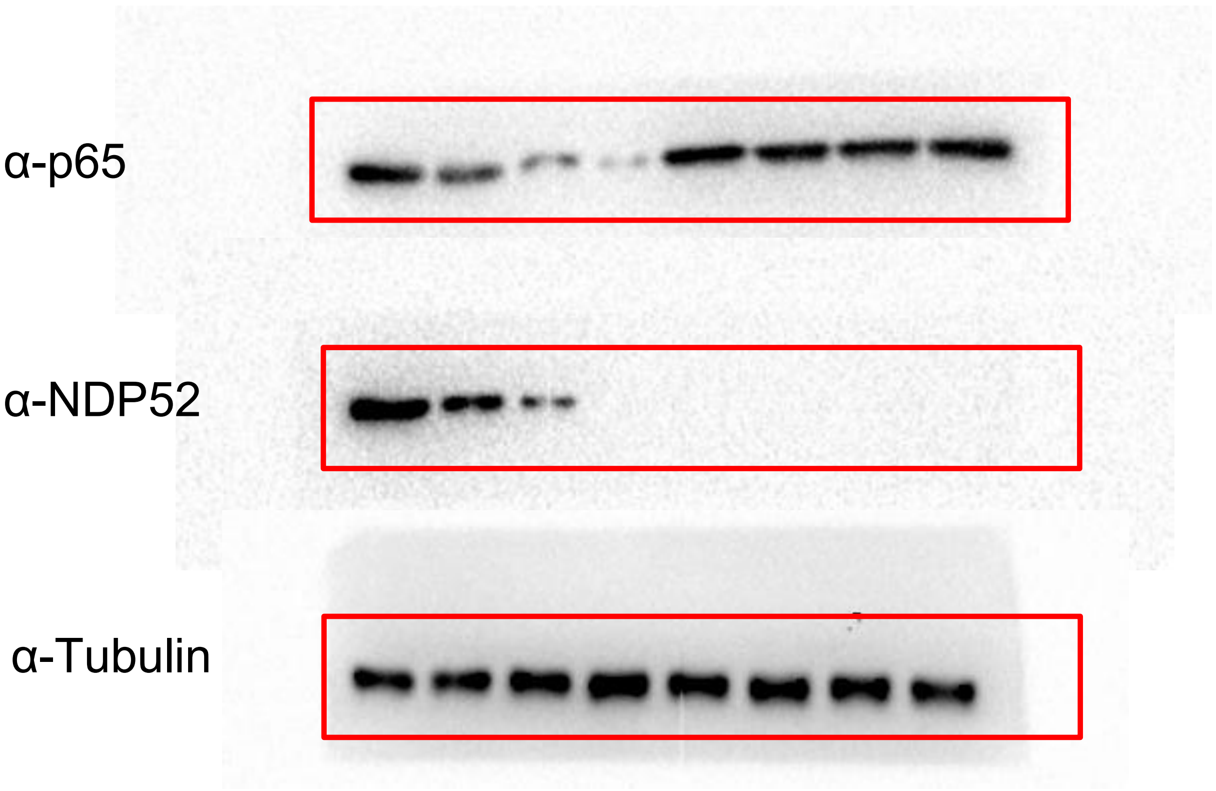

Figure 5-figure supplement 5

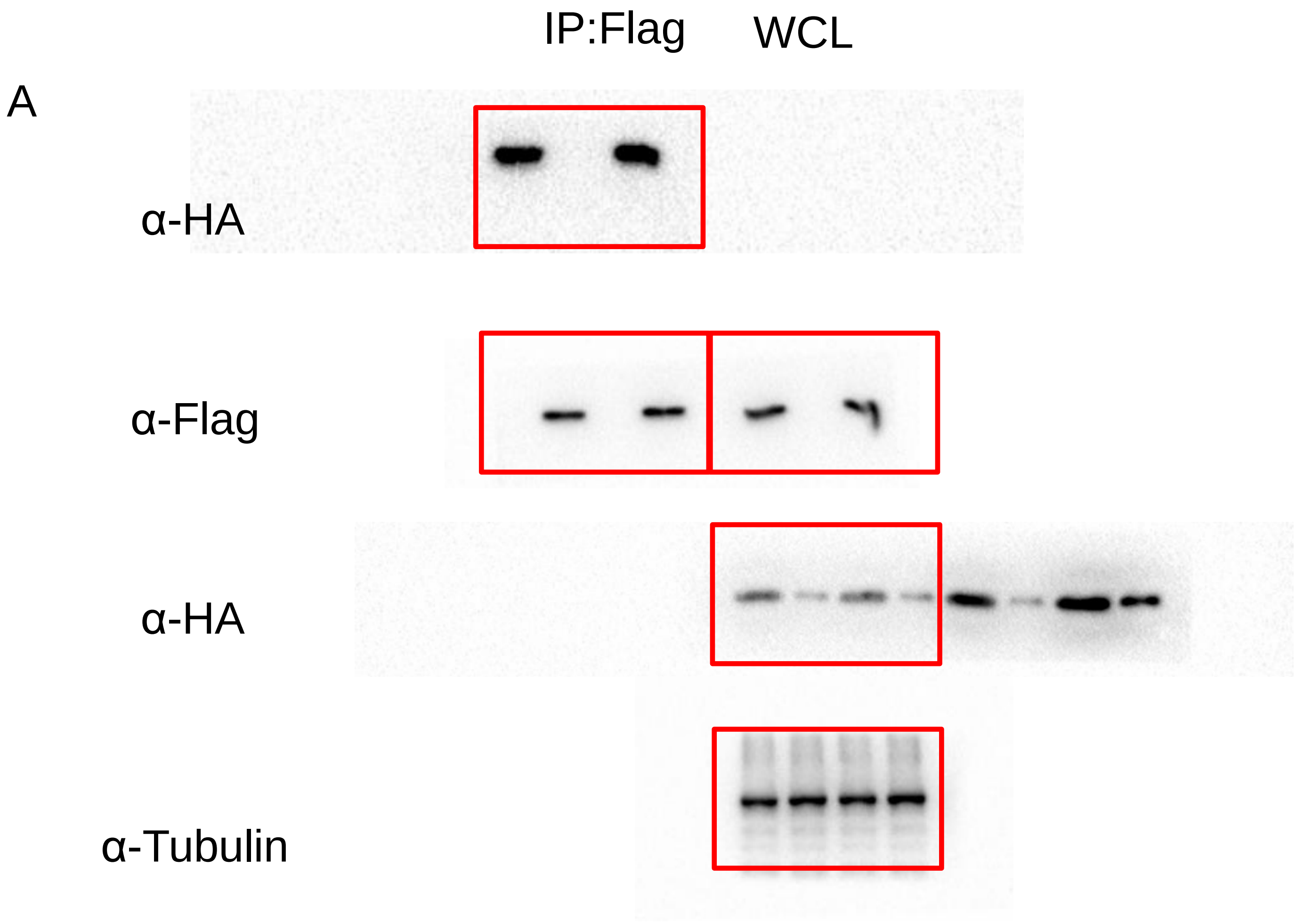

Figure 5-figure supplement 5

C

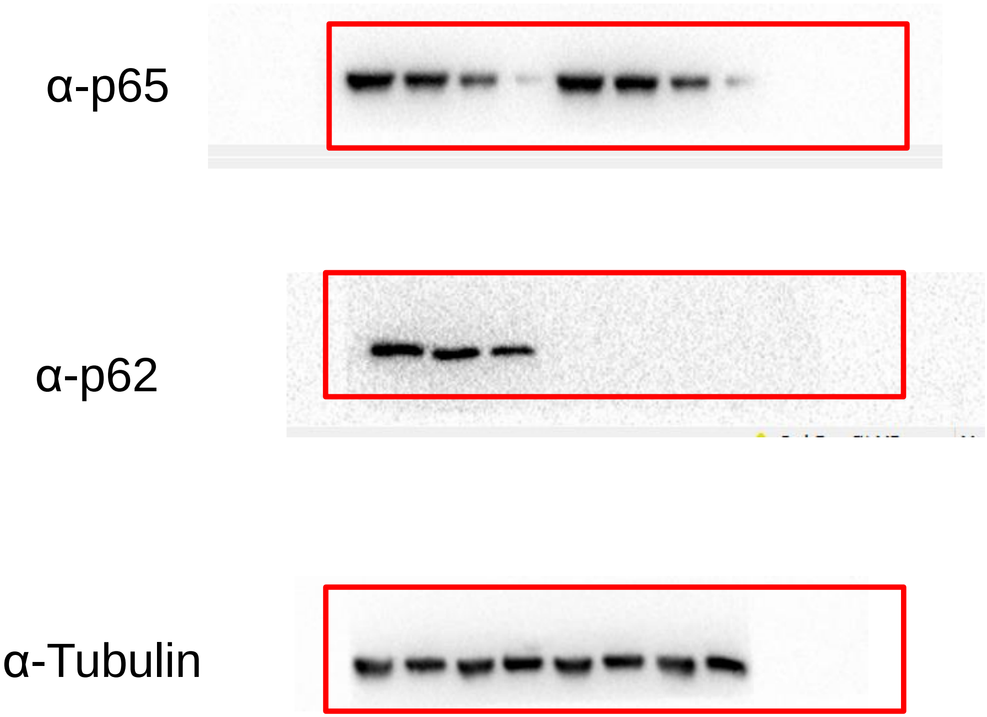

Supplement: Figure 5—source data 1. [file elife-87935-fig5-data1.pdf]
