# Supplementary material for: Vangl2 suppresses NF-κB signaling and ameliorates sepsis by targeting p65 for NDP52-mediated autophagic degradation
Source: eLife. 2024 Sep 13;12:RP87935. doi: 10.7554/eLife.87935 (PMC11398866; doi:10.7554/eLife.87935)
Supplement: Supplementary file 1. [file elife-87935-supp1.docx]

**Table S1. Reagents and antibodies used in this study.**

| **Antibody Name** | **Source** | **Catalog number** |
| --- | --- | --- |
| Vangl2 | Santa Cruz | #sc-515187 |
| phosphor-IKK-α/β (Ser^178/180^) | Cell Signaling Technology | #2697 |
| IKK-α/β | Santa Cruz | #52932 |
| Pro-IL-1β | Cell Signaling Technology | #12507 |
| IL-1β (p17) | Cell Signaling Technology | #12242 |
| phosphor-p65 (Ser^536^) | Cell Signaling Technology | #3033 |
| p65 | Cell Signaling Technology | #8242 |
| Anti-mouse CD8a | eBioscience | 17-0081-82 |
| Beclin1 | proteintech | 11306-1-AP |
| Ndp52 | proteintech | 12229-1-AP |
| Atg5 | proteintech | 10181-2-AP |
| p62 | proteintech | 18420-1-AP |
| Anti-Flag | Sigma | A8592 |
| Anti-HA | Sigma | 12013819001 |
| Anti-Myc | Beijing Ray | RM1003 |
| Anti-mouse CD11b | eBioscience | 48-0112-82 |
| Anti-mouse F4/80 | eBioscience | 17-4801-82 |
| Anti-mouse Ly6C | eBioscience | 128003 |
| Anti-mouse Ly6G | eBioscience | 127603 |
