# Supplementary material for: Vangl2 suppresses NF-κB signaling and ameliorates sepsis by targeting p65 for NDP52-mediated autophagic degradation
Source: eLife. 2024 Sep 13;12:RP87935. doi: 10.7554/eLife.87935 (PMC11398866; doi:10.7554/eLife.87935)
Supplement: Supplementary file 2. [file elife-87935-supp2.docx]

**Table S2. Primers sequences for quantitative RT-PCR**

| **Description gene/protein** | **Forward Primer Sequence** | **Reverse Primer Sequence** |
| --- | --- | --- |
| Mouse-*Gapdh* | AGGTCGGTGTGAACGGATTTG | TGTAGACCATGTAGTTGAGGTCA |
| Mouse-*Vangl2* | TGAGGGCCTCTTCATCTCC | GCCCGTGGAGTTAATTGGT |
| Mouse-*Il1b* | CACAGCAGCACATCAACAAG | GTGCTCATGTCCTCATCCTG |
| Mouse-*Il6* | CCAGTTTGGTAGCATCCATC | CTCTGGGAAATCGTGGAAAT |
| Mouse-*Tnfa* | GACGTGGAACTGGCAGAAGAG | TTGGTGGTTTGTGAGTGTGAG |
| Mouse-*Pdlim2* | TGGGGCTTCCGAATTAGCG | CGCGTGTAGCATGTTCTCTG |
| Mouse-*Usp7* | TCGTCGCACATTGAGACGG | CTTGTCGGCATGGTTGGGAAT |
| Mouse-*Trim21* | GGGAGGAGGTCACCTGTTCTA | GGCACTCGGGACATGAACTG |
| Human-*Gapdh* | GGCTGTTGTCATACTTCTCATGG | CCCTATTCCCCACAACACAC |
| Human-*Vangl2* | AATCCCGAAAAGAAGGCTGT | CCCTATTCCCCACAACACAC |
| Human-*p65* | CCCACGAGCTTGTAGGAAAGG | GGATTCCCAGGTTCTGGAAAC |
| Human-*Pdlim2* | GCCCATCATGGTGACTAAGG | ATGGCCACGATTATGTCTCC |
| Human-*Usp7* | CCAGTGCAATGCTGAATCTGA | ACGACGACTGAACGACTTTTCAT |
| Human-*Trim21* | TCAGCAGCACGCTTGACAAT | GGCCACACTCGATGCTCAC |
