# Supplementary material for: Vangl2 suppresses NF-κB signaling and ameliorates sepsis by targeting p65 for NDP52-mediated autophagic degradation
Source: eLife. 2024 Sep 13;12:RP87935. doi: 10.7554/eLife.87935 (PMC11398866; doi:10.7554/eLife.87935)
Supplement: Supplementary file 3. [file elife-87935-supp3.docx]

**Table S3. Primers sequences for siRNA transfection**

| **Description gene/protein** | **Primer Sequence** |
| --- | --- |
| Human-*Pdlim2* siRNA | 5′-GUACCAGCAUCGCGAACCATT-3′ |
| Human-*Usp7* siRNA | 5′-CCCAAAUUAUUCCGCGGCAAA-3′ |
| Human-*Trim21* siRNA | 5′-GGACAATTTGGTTGTGGAA-3′ |
| Human-*Scramble* siRNA | 5′-UUCUCCGAACGUGUCACGUTT-3′-3′ |
| Mouse-*Pdlim2* siRNA | 5′-CAGAGAUUUCCACACACCCAUCAUU-3′ |
| Mouse-*Usp7* siRNA | 5′-AGAUUUCGCACAAAACACGGA-3′ |
| Mouse-*Trim21* siRNA | 5′-CCUCUAAAGCUCUGUCCACUA-3′ |
