# Supplementary material for: Vangl2 suppresses NF-κB signaling and ameliorates sepsis by targeting p65 for NDP52-mediated autophagic degradation
Source: eLife. 2024 Sep 13;12:RP87935. doi: 10.7554/eLife.87935 (PMC11398866; doi:10.7554/eLife.87935)
Supplement: Supplementary file 4. [file elife-87935-supp4.docx]

**Table S4. The Sepsis patients’ information was shown in this study.**

| ID | Sex | Age | Admission diagnosis | CRP (mg/mL) | WBC (10^9^/L) | Heart rate (beats/min) |
| --- | --- | --- | --- | --- | --- | --- |
| 1# | F | 68 | COPD | 82.61 | 13.86 | 112 |
| 2# | F | 61 | Cholangitis | 42.58 | 23.99 | 121 |
| 3# | F | 72 | Femoral fracture | 60.31 | 18.23 | 127 |
| 4# | M | 57 | Diabetes | 55.32 | 14.33 | 122 |
| 5# | M | 68 | COPD | 62.61 | 13.99 | 124 |
| 6# | M | 72 | COPD | 52.58 | 28.23 | 140 |
| 7# | M | 78 | COPD | 70.32 | 24.33 | 128 |
| 8# | F | 82 | COPD | 45.32 | 15.34 | 110 |
| 9# | M | 25 | Health checkup | 10.23 | 6.55 | 65 |
| 10# | M | 28 | Health checkup | 5.84 | 7.56 | 68 |
| 11# | M | 27 | Health checkup | 15.32 | 5.57 | 72 |
| 12# | F | 29 | Health checkup | 14.21 | 6.85 | 75 |
| 13# | F | 26 | Health checkup | 8.22 | 4.56 | 67 |
| 14# | F | 27 | Health checkup | 5.82 | 6.02 | 66 |
| 15# | M | 30 | Health checkup | 12.32 | 5.81 | 73 |
| 16# | M | 31 | Health checkup | 9.81 | 5.73 | 77 |
